# In vivo base editing rescues liver pathophysiology and peroxisome dysfunction in a mouse model of Zellweger spectrum disorder

**DOI:** 10.1038/s41551-026-01651-5

**Published:** 2026-04-14

**Authors:** Xin D. Gao, Maximiliano Presa, Jordyn E. Duby, Jennifer Ryan, Pierre-Alexandre Piec, Alvin Hsu, Samagya Banskota, Allen Yujie Jiang, Lingxiao Chen, Gregory A. Newby, Erminia Di Pietro, Jonathan M. Levy, Bradford H. Steele, Sarah Lecordier, Fangfei Qin, Ann B. Moser, Jun Xie, Guangping Gao, Nancy E. Braverman, Aamir R. Zuberi, Joseph G. Hacia, Cathleen M. Lutz, David R. Liu

**Affiliations:** 1 Merkin Institute of Transformative Technologies in Healthcare, Broad Institute of MIT and Harvard, Cambridge, MA, USA.; 2 Department of Chemistry and Chemical Biology, Harvard University, Cambridge, MA, USA.; 3 Howard Hughes Medical Institute, Harvard University, Cambridge, MA, USA.; 4 Department of Ophthalmology, University of Pittsburgh School of Medicine, Pittsburgh, PA, USA.; 5 Rare Disease Translational Center, The Jackson Laboratory, Bar Harbor, ME, USA.; 6 The Research Institute of the McGill University Health Centre, Montreal, Quebec, Canada.; 7 Department of Cancer Biology, Keck School of Medicine of the University of Southern California, Los Angeles, CA, USA.; 8 Whitehead Institute for Biomedical Research/Massachusetts Institute of Technology, Cambridge, MA, USA.; 9 Peroxisomal Disease Laboratory, Hugo W Moser Research Institute at Kennedy Krieger, Baltimore, MD, USA.; 10 Department of Genetic and Cellular Medicine, University of Massachusetts Chan Medical School, Worcester, MA, USA.; 11 Technology Evaluation and Development Research Laboratory, The Jackson Laboratory, Bar Harbor, ME, USA.; 12 These authors contributed equally: Xin D. Gao, Maximiliano Presa.

## Abstract

Zellweger spectrum disorder (ZSD) is caused by biallelic loss-of-function variants in *PEX* genes required for peroxisome biogenesis, which is critical for normal cellular metabolism and signalling. The *PEX1*-p.G843D (c.2528G>A) allele, present in approximately 30% of individuals with ZSD, frequently results in chronic liver disease that can progress to cirrhosis, hepatocellular carcinoma and degraded neurological health. Here we report the development and application of an adenine base editing strategy to correct an established homozygous *Pex1*-p.G844D ZSD mouse model that manifests liver pathologies and metabolic dysfunction found in patients. Through intravenous delivery of AAV9 encoding ABE8e-V106W into both neonatal and 4-week-old mice, we achieved up to 60% pathogenic allele correction in the bulk liver. By restoring peroxisome function, base editing eliminated bulk accumulation of very long-chain and branched-chain fatty acids, and toxic C27-bile acid intermediates. Increased levels of phytanic acid, a branched-chain fatty acid that becomes harmful when accumulated, were normalized in blood, liver and brain tissue. Treatment of homozygous *Pex1*-p.G844D mice resulted in the progressive, dose-dependent normalization of liver transcriptomes and histopathology, accompanied by gains in body weight. Non-viral lipid nanoparticle delivery of ABE8e-V106W mRNA to 4-week-old mice also yielded correction of the *Pex1*-p.G844D allele in 27% of bulk liver cells. In patient-derived fibroblasts, base editing corrected >80% of *PEX1*-p.G843D alleles and restored peroxisome homeostasis. Genome-wide experimental and computational off-target analyses found minimal off-target editing in the mouse or human genome. Collectively, these findings suggest that liver base editing over a range of ages may benefit individuals with ZSD and provides a foundation for developing precision gene correction treatments that address the root cause of a wide range of peroxisomal disorders.

Peroxisomes are metabolic membrane-bound organelles that support eukaryotic cell signalling^1–3^ and physiological homeostasis in mammals^1,4^. They are required for biosynthesis of mature bile acids, platelet-activating factor, docosahexaenoic acid and ether lipids (for example, plasmalogens), major constituents of myelin^5^. They are involved in the catabolism of branched-chain and very long-chain (≥22 carbons) fatty acids (BCFAs and VLCFAs) and hydrogen peroxide by-products of lipid oxidation^6–8^. Peroxisomes are also involved in the aetiology of rare and common diseases^9^ and play essential roles in the development and function of mammalian hepatic, nervous and sensory systems^3,10^.

ZSD is a disease spectrum with an overall incidence of approximately 1 in 50,000–90,000 births in North America^10,11^. ZSD is caused by biallelic loss-of-function variants in any of 13 *PEX* genes encoding peroxins required for normal peroxisome homeostasis^10^. PEX1 is a member of the AAA ATPase protein family and an essential component of the PEX1–PEX6–PEX26 exportomer complex required for peroxisome assembly^12–14^. The partial loss-of-function *PEX1*-p.G843D (c.2528G>A) variant represents approximately 30% of all ZSD alleles^10^ (Fig. 1a). Consistent with residual activity, patients with at least one *PEX1*-p.G843D allele typically show milder phenotypes and a more degenerative course of disease than patients with two null *PEX1* gene alleles, who often do not survive beyond the first year of life^10,15^. Individuals who are compound heterozygous for the *PEX1*-p.G843D allele and a null *PEX1* allele typically present with moderate disease severity. Although precise genotype information is often limited, individuals with moderate ZSD frequently display hepatomegaly, coagulation defects and progressive liver cholestasis that can progress to cirrhosis, hepatocellular carcinoma and shortened lifespan^15–17^. In addition, they can show progressive vision and hearing loss, intellectual disabilities, osteopenia, kidney stones, adrenal insufficiency and amelogenesis imperfecta^15,17–19^. Individuals homozygous for the *PEX1*-p.G843D allele commonly show a milder course of degenerative disease that typically results in progressive vision and hearing loss and amelogenesis imperfecta with hepatomegaly, coagulation defects and adrenal insufficiency in some individuals^15,18,20^.

ZSD can be detected before substantial irreversible damage to the liver and other organs in individuals with less severe disease, providing therapeutic windows for intervention. Newborn screening for adrenoleukodystrophy, which measures C26:0-lysophosphatidylcholine (LPC) levels in dried blood spots, can also detect ZSD, because LPC is increased in both conditions^21,22^. Early diagnosis highlights the urgent unmet need for developing effective therapies for ZSD since current treatments do not address the root cause of disease^23^. Cholic acid supplementation can suppress bile acid synthesis and reduce the accumulation of toxic C27-bile acid intermediates caused by impaired peroxisomal beta-oxidation; however, its clinical benefit remains uncertain^24–26^. Although transplantation has been used to address liver disease, these are invasive procedures that require long-term immunosuppression, and few cases of individuals with ZSD receiving transplants have been reported^27–30^.

Base editing is a precision genome editing tool that typically uses a DNA deaminase tethered to a Cas9 nickase to enable targeted, single-nucleotide conversion. Adenine base editors (ABEs), as one class of base editors, can convert A•T-to-G•C and have demonstrated potential in the treatment of genetic diseases in ex vivo and in vivo, such as in recent examples of progeria^31^, sickle cell disease^32^, CD3δ severe combined immunodeficiency^33^ and spinal muscular atrophy^34,35^. Here we developed an adenine base editing strategy to directly correct the *Pex1*-p.G844D (c.2531G>A) allele, the murine equivalent of the human *PEX1*-p.G843D allele, in a preclinical ZSD mouse model. This model is homozygous for the *Pex1*-p.G844D allele and recapitulates key disease features such as fatty liver with cholestasis and progressive vision loss^36–40^. We delivered dual adeno-associated virus (AAV) vectors (encoding ABE8e-V106W and a guide RNA (gRNA) that targets the ABE to *Pex1*-p.G844D) to neonatal and 4-week-old mice and achieved robust correction of disease alleles in bulk liver in a highly specific dose- and time-dependent manner. Lipid nanoparticle (LNP) delivery of the ABE8e-V106W mRNA and *Pex1* gRNA also yielded substantial correction of the pathogenic allele in bulk liver, demonstrating the possibility of a non-viral in vivo base editing approach.

AAV9-ABE-mediated correction of the murine *Pex1* pathogenic allele restored normal hepatic metabolic functions, reduced circulating and/or tissue levels of toxic peroxisome metabolites, rescued liver pathologies and improved growth. In cultured patient-derived skin fibroblasts, ABE editing resulted in the robust correction of *PEX1*-p.G843D alleles and recovered peroxisome homeostasis. Our results establish that a precision gene editing strategy can correct the root cause of ZSD liver disease and rescue its phenotypic consequences in animal models and patient-derived cells.

## Results

### The *PEX1*-p.G843D allele is associated with liver disease in individuals with ZSD

**Fig. 1 |:**
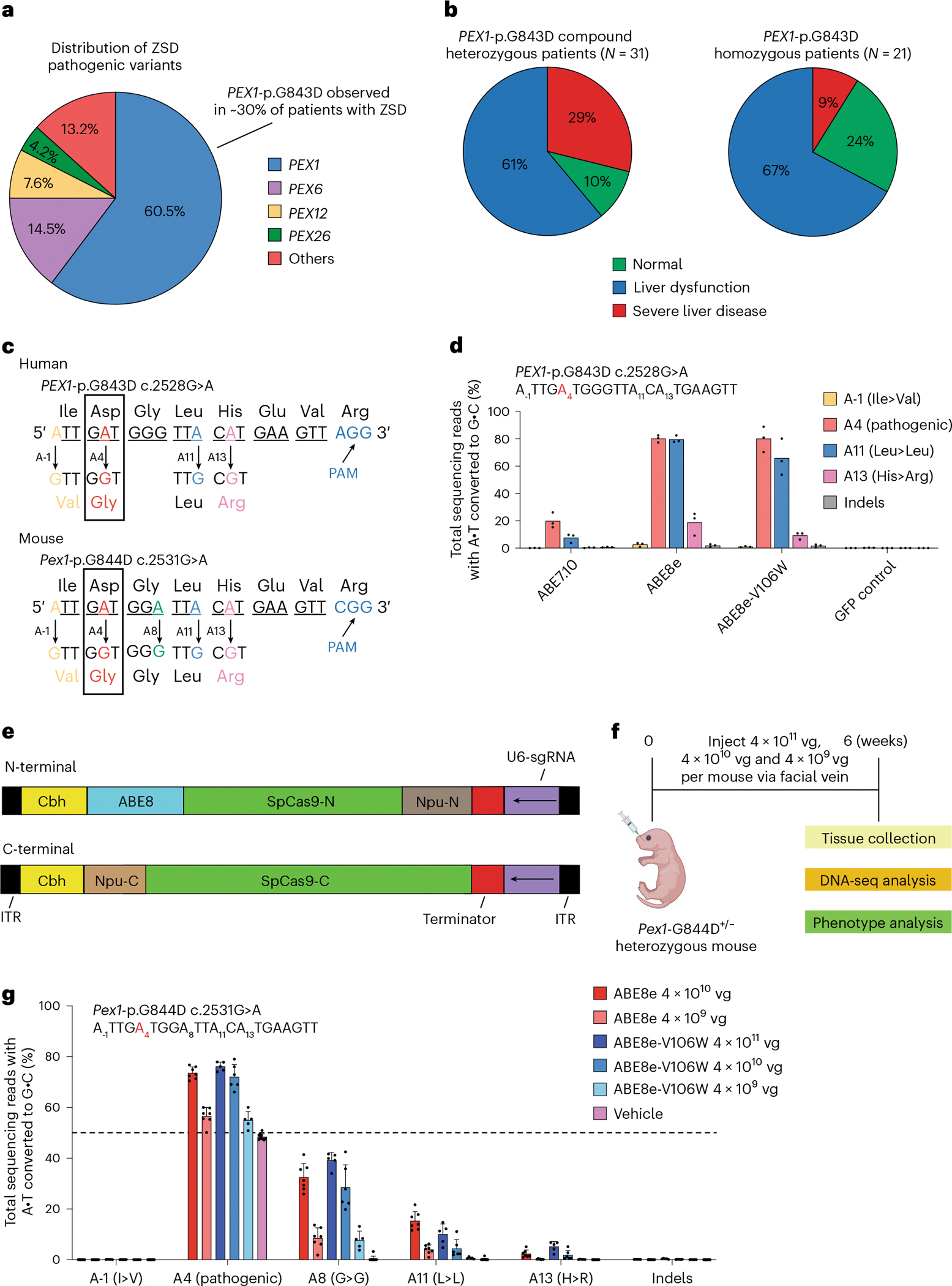
Assessment of adenine base editing strategies to correct *PEX1*-p.G843D in cultured patient fibroblasts and *Pex1*-p.G844D in heterozygous mice. **a**, Distribution of ZSD pathogenic variants among *PEX* genes^10^. **b**, Clinical analysis of liver disease in individuals with *PEX1*-p.G843D ZSD. Patients were categorized into three groups: normal liver, liver dysfunction and severe liver disease. Of the 52 patients analysed, 21 are *PEX1*-p.G843D homozygous, and 31 are *PEX1*-p.G843D compound heterozygous. Sex, disease variant genotypes and age of the patients are listed in Source data. **c**, Strategy for using SpCas9 ABEs to correct the human *PEX1*-p.G843D c.2528G>A and the mouse *Pex1*-p.G844D c.2531G>A pathogenic variants. Synonymous and non-synonymous bystander edits are shown, and protospacer sequences are underlined. **d**, Assessment of adenine base editing in patient-derived human *PEX1*^G843D/G843D^ fibroblasts. Editing efficiency was measured 3 days after electroporation via HTS. **e**, Schematic of dual-AAV9 vectors for in vivo delivery of ABE8 variants. ITR, inverted terminal repeats. **f**, Schematic of facial vein injection of P_1_ *Pex1*^G844D/+^ heterozygous mice with AAV9-ABE vectors to correct the *Pex1*-p.G844D pathogenic allele. **g**, Base editing efficiencies quantified via HTS and CRISPResso2^99^ analysis of liver samples from vehicle (saline, *n* = 10) or ABE-treated *Pex1*^G844D/+^ heterozygous mice (ABE8e 4 × 10^10^ vg *n* = 7; ABE8e 4 × 10^9^ vg *n* = 7; ABE8e-V106W 4 × 10^11^ vg *n* = 5; ABE8e-V106W 4 × 10^10^ vg *n* = 6; ABE8e-V106W 4 × 10^9^ vg *n* = 5). The adenines within and near the protospacer are shown. Dots indicate values from *n* = 3 independent replicates (**d**) and for individual mice (**g**). Bars represent the mean of individual values. Base editing efficiencies shown reflect the frequency of the intended base editing outcome with no indels. Error bars indicate standard deviation unless otherwise specified. Mouse diagram in **f** created in BioRender; Piec, M. https://biorender.com/n8hnab4 (2026).

To better understand the natural course of ZSD liver disease caused by the *PEX1*-p.G843D allele(s), we analysed clinical data from 52 individuals (age of assessment 0.04–49.9 years) in a longitudinal ZSD natural history study (ClinicalTrials.gov ID NCT01668186) who were either homozygous or compound heterozygous for this allele. Evidence of liver disease was subclassified into liver dysfunction (including increased liver transaminases and hepatomegaly) and severe liver disease (including cirrhosis, portal hypertension, esophageal varices, gastrointestinal bleeding, ascites or hepatic cancer). There was frequent evidence of liver dysfunction or severe liver disease in patients homozygous for the *PEX1*-p.G843D allele (67%, liver dysfunction; 9%, severe disease) or compound heterozygous for the *PEX1*-p.G843D allele (61%, liver dysfunction; 29%, severe disease) (Fig. 1b). On average, *PEX1*-p.G843D homozygote patients, who have two *PEX1* alleles with residual activity, showed less severe liver disease than compound heterozygous patients, who could also carry a *PEX1* null allele (indeed, frameshift or nonsense variants were found in 24 such patients). Overall, these data support the unmet need for therapies for ZSD liver disease caused by *PEX1*-p.G843D allele(s).

### Development of a base editing strategy to correct the *PEX1*-p. G843D allele in patient-derived fibroblasts

To correct the *PEX1*-p.G843D (c.2528G>A) variant, we designed a *Streptococcus pyogenes* Cas9 (SpCas9) gRNA that places the c.2528G>A adenine variant at the fourth position (A4) of the protospacer 5′-TTG**A**TGGGTTACATGAAGTT-3′, located within the 5-nt base editing window (5′-N_4_NNNN_8_-3′) for most base editors that use SpCas9^41^ (Fig. 1c). Next, we tested three different ABEs that use SpCas9 domains to recognize NGG protospacer-adjacent motif (PAM) sequences: ABE7.10^42^, ABE8e^43^ and ABE8e-V106W^43^. Laboratory-evolved deoxyadenosine deaminases ABE8e and ABE8e-V106W offer higher editing efficiencies than ABE7.10. ABE8e-V106W is a high-fidelity version of ABE8e that results in efficient on-target editing but reduced RNA editing and Cas-independent off-target DNA editing owing to a V106W substitution in the deaminase^43^. After nucleofecting patient-derived homozygous *PEX1*-p.G843D skin fibroblasts with ABE7.10 mRNA and synthetic gRNA, 20% of total sequencing reads contained the corrected base edit (from the pathogenic A4 to wild-type (WT) G4) and 7.6% of sequencing reads showed bystander editing (synonymous mutation) at protospacer position A11 (Fig. 1d). Using ABE8e and ABE-V106W to target this protospacer yielded a 4-fold improvement in editing efficiency in patient-derived fibroblasts compared with ABE7.10, achieving 80% correction with ABE8e and ABE8e-V106W. This large improvement in editing efficiency was accompanied by increased bystander editing at positions A11 and A13. Given the enhanced adenine deaminase activity, we expected some bystander editing at these neighbouring bases^43^. Bystander A•T-to-G•C editing at A11 results in a synonymous mutation (p.L845L, TTA>TTG) predicted not to impact *PEX1* RNA splicing^44^ (Fig. 1d). Bystander editing at position A13 introduces a missense mutation (p.H846R) predicted to be benign by the variant classification algorithms tested^44,45^ and gnomAD^46^, and has not been reported in ClinVar^47^. We observed bystander editing (A13) at a frequency of 19% with ABE8e and 9.3% with ABE8e-V106W, respectively, and low levels of indels (<2%) within 20 bp of the protospacer in all ABE-treated fibroblasts. Further assessment of recently reported compact ABE editors in the homozygous *PEX1*-p.G843D skin fibroblasts showed largely reduced editing efficiency for this pathogenic allele compared with ABE8e (Supplementary Note 1).

These results demonstrate that adenine base editing can efficiently correct the *PEX1*-p.G843D variant in patient-derived cells while minimizing indels. We, therefore, chose to apply these ABE8e and ABE8e-V106W *PEX1*-p.G843D correction strategies in vivo using mouse models of ZSD.

### In vivo adenine base editing efficiently corrects the mouse *Pex1*-p.G844D allele

To investigate the potential of an ABE-mediated therapeutic strategy for ZSD, we designed a SpCas9 gRNA to target the *Pex1*-p.G844D murine equivalent of the *PEX1*-p.G843D allele. The mouse allele differs from the corresponding *PEX1*-p.G843D human allele at the target protospacer by a single nucleotide at the eighth position (5′-TTGATGG**A**TTACATGAAGTT-3′). We designed a dual-AAV9 vector system that encodes the ABE as two halves, each linked to a fast-splicing intein^48^ (Fig. 1e). Following the co-transduction of AAV encoding both ABE halves, the complete base editor can form upon protein splicing or association of the two halves^48^.

To determine the ability of ABE8 variants to correct the *Pex1*-p.G844D allele in the liver of mice, we treated P_1_ *Pex1*^G844D/+^ heterozygous mice with one of three doses of AAV9 (4 × 10^11^ vg, 4 × 10^10^ vg or 4 × 10^9^ vg per mouse) encoding ABE8e or ABE8e-V106W and the above gRNA by facial vein injection (Fig. 1f). Heterozygous mice did not tolerate the highest dose of ABE8e (4 × 10^11^ vg per mouse, equivalent to 2.8 × 10^14^ vg kg^−1^); all mice in this cohort died by day 15 after injection. By contrast, all five mice successfully injected with the ABE8e-V106W base editor at the same dose of 4 × 10^11^ vg per mouse survived. Six weeks after injection, the mice were killed, and liver tissue analysis revealed 52% bulk correction of *Pex1*-p.G844D pathogenic alleles, the highest editing efficiency among all surviving cohorts (Fig. 1g). At 4 × 10^10^ vg and 4 × 10^9^ vg doses, ABE8e did not lead to any lethality and yielded 47% and 13% correction of the *Pex1*-p.G844D alleles. We observed 44% and 10% correction in ABE8e-V106W injected P_1_ mice dosed with 4 × 10^10^ vg and 4 × 10^9^ vg, respectively. We also observed very low levels of undesired bystander editing (<0.1% for A at the minus one position relative to the protospacer, p.I843V) and indels (<0.55%) across all mice treated with either ABE variant.

Collectively, these data in heterozygous mice establish that AAV-mediated in vivo delivery of ABE can correct the *Pex1*-p.G844D allele in animals. Moreover, these results suggested an AAV9-ABE8e-V106W dose range to evaluate potential phenotypic rescue in the homozygous *Pex1*-p.G844D mouse model. As we observed high editing efficiency and no evident toxicity when using AAV9 as a delivery vehicle, we advanced the ABE8e-V106W base editor to in vivo phenotypic rescue studies with homozygous *Pex1*-p.G844D mice.

### In vivo base editing in homozygous *Pex1*-p.G844D mice

Homozygous *Pex1*-p.G844D mice have a severe neonatal mortality when bred in the B6/J (B6.Cg-*Pex1*^*tm1.1Sjms*^/Mmjax, catalogue 25065)^39^ or B6/N^37^ genetic background strains. By contrast, mixed genetic background homozygous *Pex1*-p.G844D mice have been previously shown to have a more prolonged survival^36,39^. Here we used a more defined breeding strategy by crossing B6.Cg-*Pex1*^*tm1.1Sjms*^/Mmjax with 129S6. Cg-*Pex1*^*tm1.1Sjms*^/Mmjax. The use of F_1_ hybrid mice improves reproducibility and reduces phenotypic variation owing to segregating genetic modifiers. The resulting homozygous mice for the *Pex1*-p.G844D allele have normal survival, but show growth deficits, retinal degeneration^38,49^ and liver pathologies resulting from impaired peroxisomal function.

**Extended Data Fig. 1 |:**
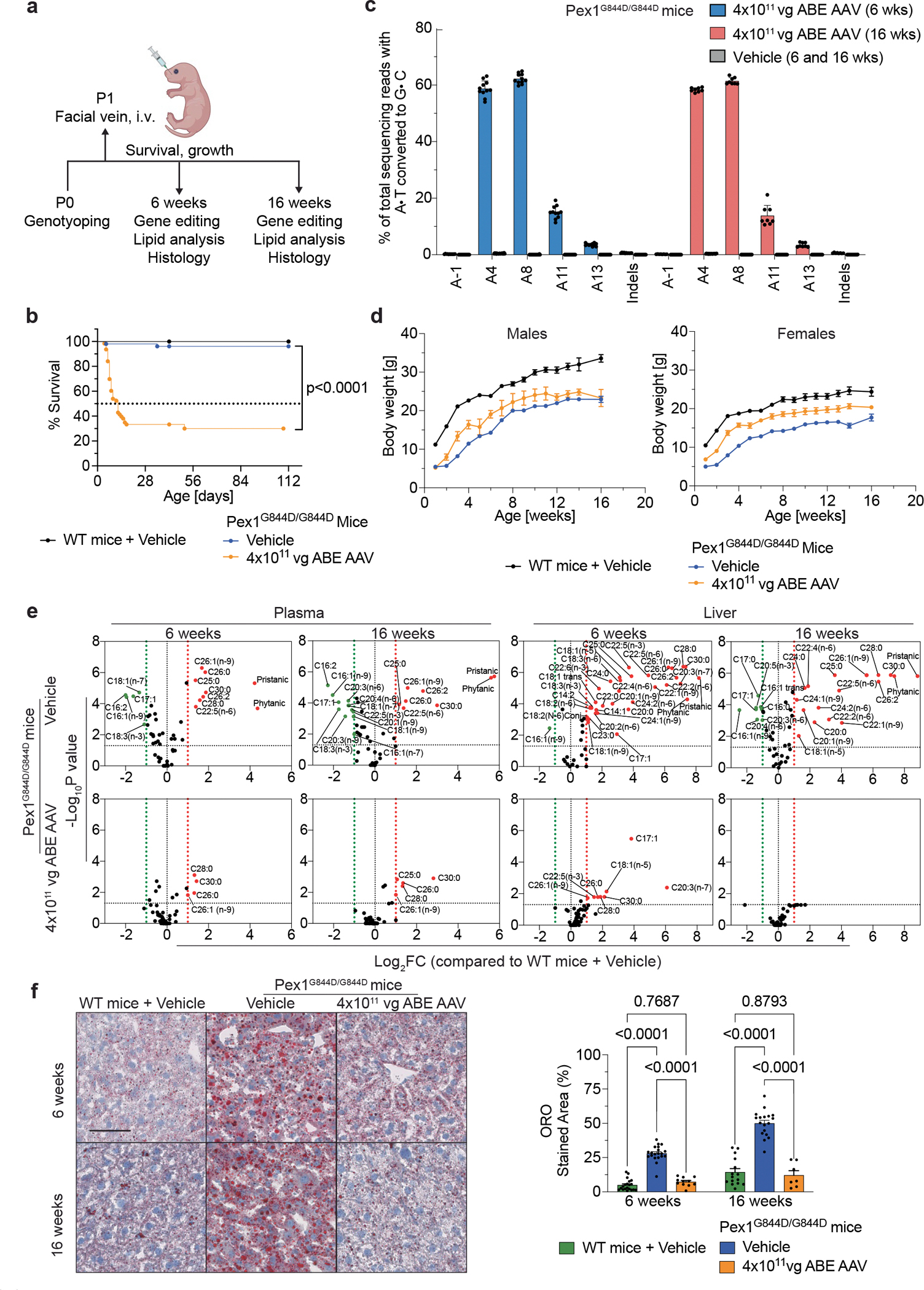
Characterization of *Pex1*^G844D/G844D^ neonatal mice treated with AAV9-ABE8e-V106W. **a**, AAV9-ABE8e-V106W (4×10^11^ vg per mouse) was delivered via facial vein injection into P_1_ *Pex1*^G844D/G844D^ mice (n = 63). Vehicle-injected WT (*Pex1*^+/+^) mice (n = 40) and *Pex1*^G844D/G844D^ mice (n = 52) served as controls. Animals were randomized from multiple litters. **b**, Kaplan-Meier survival curves for AAV9-ABE-treated and vehicle-treated mice (n as in **a**). **c**, Editing efficiencies in liver at 6–16 weeks post-treatment, measured by high-throughput sequencing (HTS) in vehicle-treated mice (6 weeks n = 20; 16 weeks n = 19) and AAV9-ABE -treated mice (6 weeks n = 11; 16 weeks n = 8). Adenines within and near the protospacer are plotted. Bars indicate mean ± s.d.; dots represent individual mice. **d**, Growth curves for treated and control mice shown as mean ± s.e.m. of body weight for females and males (n as in **a**). **e**, Lipid profile of plasma and liver at 6–16 weeks, analyzed by gas chromatography-mass spectrometry (GC-MS). Lipids fold-change relative to vehicle-treated WT mice is shown in volcano plots. Green and red dotted lines indicate two-fold decrease and increase; black dashed line marks P = 0.05. P values were calculated using Dunn’s nonparametric many-to-one comparison test for Kruskal-type ranked data. **f**, Liver lipid content by Oil-Red O (ORO) staining at 6–16 weeks in vehicle-treated WT mice (6 weeks n = 20; 16 weeks n = 15), *Pex1*^G844D/G844D^ mice (6 weeks n = 20; 16 weeks n = 18), and AAV9-ABE-treated *Pex1*^G844D/G844D^ mice (6 weeks n = 11; 16 weeks n = 7). Images captured at 20x magnification; scale bar, 50 μm. Statistical analysis by two-way ANOVA with Sidak’s test; significance was set at P < 0.05. Bars represent mean ± s.e.m; dots represent individual mice. Numbers above bars indicate P-values for specified comparisons. Mouse diagram in **a** created in BioRender; Piec, M. https://biorender.com/n8hnab4 (2026).

To test the potential of base editing to restore peroxisome and liver function, we delivered dual-AAV9-ABE8e-V106W to P_1_ *Pex1*^G844D/G844D^ pups systemically, via facial vein injection (Extended Data Fig. 1a). All vehicle (saline)-treated homozygous mice survived until 16 weeks post-injection, the endpoint of the study, confirming that the B6129S6 F_1_ hybrid background addressed the neonatal lethality observed in pure B6 or pure 129S6 mouse strains (Extended Data Fig. 1b). While the 4 × 10^11^ vg AAV9-ABE8e-V106W dose did not affect the viability of healthy heterozygous *Pex1*^G844D/+^ mice, *Pex1*^G844D/G844D^ mice were less tolerant of this dose, most likely owing to their reduced weight at P_1_ (1.4 g homozygous versus 1.6 g heterozygous on average) and pronounced liver disease. Among the 19 (44 out of 63 mice died) surviving mice, we observed an average of 59% bulk liver correction of the G844D allele. Since hepatocytes account for the majority (~60%) of mouse liver cells^50^, this degree of bulk liver editing is consistent with robust hepatocyte editing (Extended Data Fig. 1c). The body weight of *Pex1*^G844D/G844D^ mice treated with ABE8e-V106W improved compared with untreated controls (Extended Data Fig. 1d). All 19 mice showed a normalization of hepatic lipid profiles with decreased levels of free and esterified VLCFAs and BCFAs by gas chromatography/mass spectrometry (GC/MS) (Extended Data Fig. 1e). Further histopathology analysis showed a reduction of lipid accumulation in the liver (Extended Data Fig. 1f), suggesting restored peroxisome function and rescued liver steatosis.

**Extended Data Fig. 2 |:**
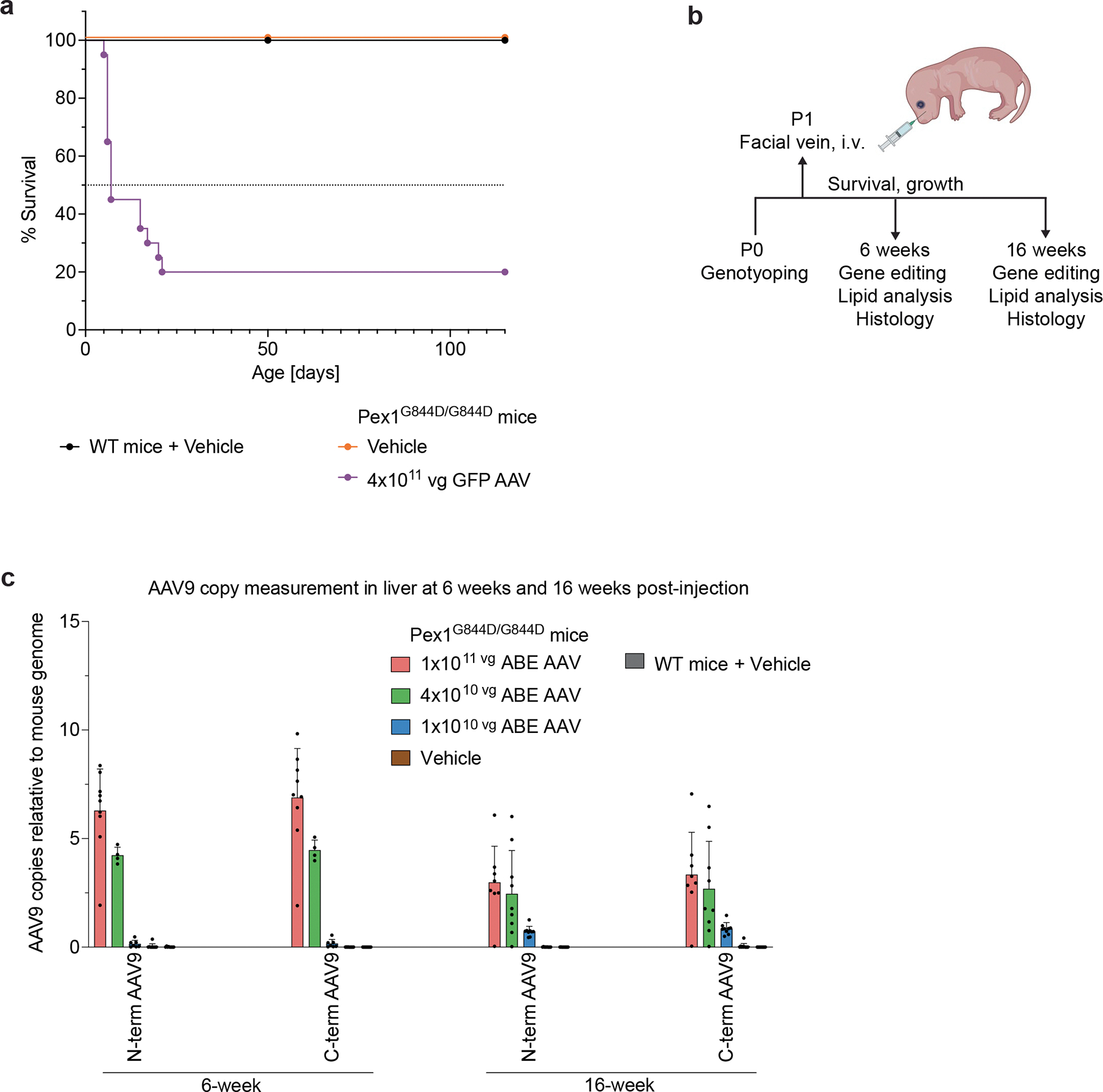
AAV9-GFP *in vivo* study and AAV9-ABE8e-V106W treatment of *Pex1*^G844D/G844D^ neonatal mice rescues ZSD phenotypes. **a**, Kaplan-Meier survival curves for WT (n = 20), and *Pex1*^G844D/G844D^ vehicle-treated (n = 21); and *Pex1*^G844D/G844D^ mice treated with AAV9-GFP (4×10^11^ vg per mouse; n = 20). **b**, Schematic of ABE treatment in *Pex1*^G844D/G844D^ mice. Homozygous mice were injected at P1 via facial vein with AAV9-ABE8e-V106W at total doses of 1×10^11^ vg (n = 18; 9 females, 9 males), 4×10^10^ vg (n = 13; 7 females, 6 males), or 1×10^10^ vg per mouse (n = 18; 8 females, 10 males). WT (n = 19; 9 females, 10 males) and *Pex1*^G844D/G844D^ mice (n = 21; 12 females, 9 males) injected with vehicle served as control. Animals were randomized from multiple litters. **c**, Quantification of N- and C-terminal components of dual AAV9 viral vectors carrying the ABE8e-V106W editor by droplet digital PCR (ddPCR) at 6 and 16 weeks using vector-specific fluorescent probes. Liver gDNA was extracted from vehicle-treated WT mice (6 weeks n = 9; 16 weeks n = 10), vehicle-treated *Pex1*^*G844D/G844D*^ mice (6 and 16 weeks n = 10), and AAV9-ABE8e-V106W-treated *Pex1*^*G844D/G844D*^ mice at doses of 1×10^*11*^ vg (6 weeks n = 9; 16 weeks n = 8), 4×10^*10*^ vg (6 weeks n = 4; 16 weeks n = 9), or 1×10^*10*^ vg (6 weeks n = 7; 16 weeks n = 9) per mouse. Bars represent mean ± s.e.m.; dots indicate individual mice. Mouse diagram in **b** created in BioRender; Piec, M. https://biorender.com/n8hnab4 (2026).

**Extended Data Fig. 3 |:**
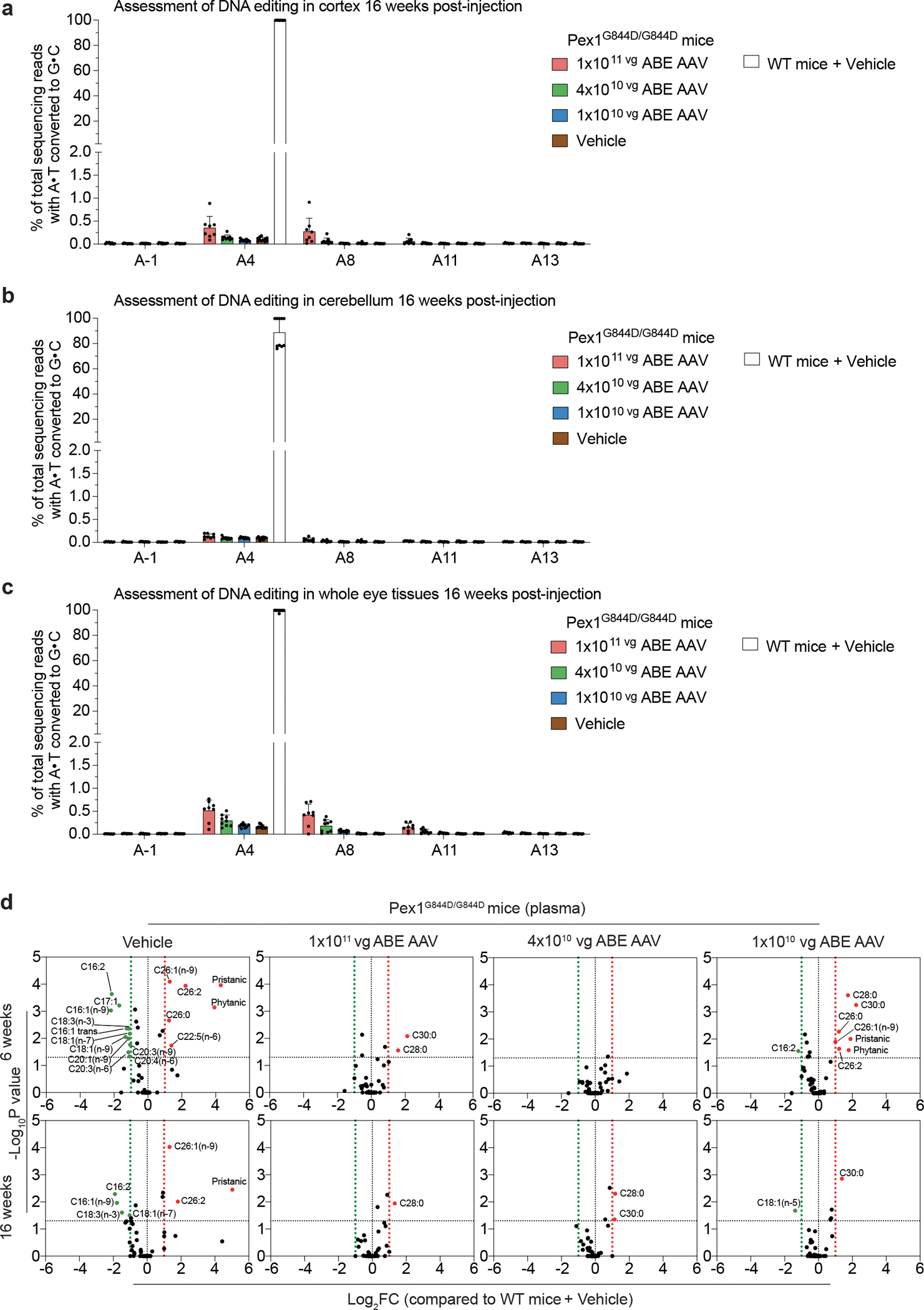
Assessment of ABE8e-V106W editing in CNS tissues from *Pex1*^G844D/G844D^ neonatal mice treated with three AAV9 doses at P1. **a**, Base editing efficiencies were quantified via HTS and analyzed with CRISPResso2. Brain cortex tissues were collected from vehicle-treated WT and *Pex1*^G844D/G844D^ mice, or AAV9-ABE8e-V106W-treated *Pex1*^G844D/G844D^ mice (1×10^11^ vg, 4×10^10^ vg, or 1×10^10^ vg per mouse) at 16 weeks. Adenines within and near the protospacer are shown. **b**, Quantification of base editing efficiencies in the cerebellum. **c**, Quantification of base editing efficiencies in whole eye tissue. For AAV9-ABE-V106W dosing groups, n = 8 mice (1×10^11^ vg), n = 9 mice (4×10^10^ vg), n = 9 mice (1×10^10^ vg). Vehicle-treated *Pex1*^G844D/G844D^ mice, n = 11; vehicle-treated WT mice, n = 10. **d**, Lipid profile of plasma samples collected at 6–16 weeks (n as in **b**), analyzed by GC-MS. Fold-change in lipid abundance was calculated relative to vehicle-treated WT mice and shown in volcano plots. Green and red dotted lines represent twofold decrease and increase in lipid abundance, respectively; black dashed line marks P = 0.05. P values were calculated relative to vehicle-treated WT samples using Dunn’s nonparametric many-to-one comparison test for Kruskal-type ranked data.

**Fig. 2 |:**
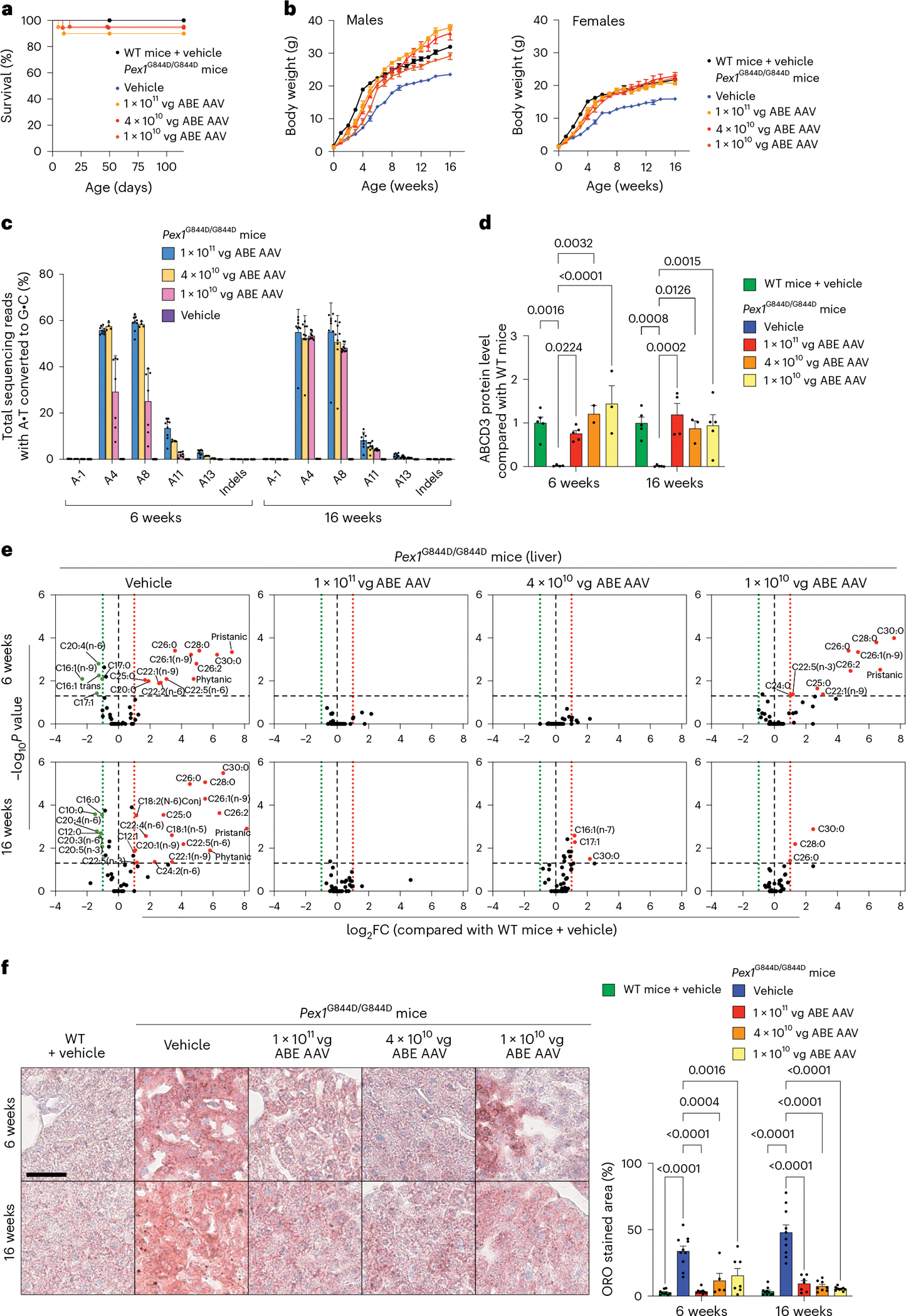
AAV9-ABE8e-V106W treatment rescues growth, peroxisome function and liver steatosis in *Pex1*^G844D/G844D^ neonatal mice. **a**, Kaplan–Meier survival curves for WT mice (*n* = 19; 9 females, 10 males) and *Pex1*^G844D/G844D^ mice treated with vehicle (*n* = 21; 12 females, 9 males) or AAV9-ABE8e-V106W at 1 × 10^11^ vg (*n* = 18; 9 females, 9 males), 4 × 10^10^ vg (*n* = 13; 7 females, 6 males) or 1 × 10^10^ vg (*n* = 18; 8 females, 10 males) per mouse. **b**, Growth curves showing mean ± s.e.m. body weight for males and females (*n* as in **a**). **c**, Editing efficiencies in liver by HTS for vehicle-treated (*n* = 4) and ABE AAV-treated mice at 1 × 10^11^ vg (*n* = 5), 4 × 10^10^ vg (*n* = 3) and 1 × 10^10^ vg (*n* = 4). Adenines within and near the protospacer are plotted. **d**, Peroxisome biogenesis assessed by automated western blot for ABCD3 in liver at 6 weeks (WT *n* = 5; and *Pex1*^G844D/G844D^ treated with vehicle *n* = 4; 1 × 10^11^ vg *n* = 5; 4 × 10^10^ vg *n* = 2; 1 × 10^10^ vg *n* = 3) and 16 weeks (WT *n* = 5; and *Pex1*^G844D/G844D^ treated with vehicle *n* = 5; 1 × 10^11^ vg *n* = 4; 4 × 10^10^ vg *n* = 3; 1 × 10^10^ vg *n* = 5). FC relative to WT. **e**, Liver lipid profiles at 6 and 16 weeks measured by GC-MS; volcano plots show FC relative to WT (*n* as in **a**). Green and red dotted lines represent twofold decrease and increase; dashed line marks *P* = 0.05. *P* values were calculated by Dunn’s nonparametric many-to-one comparison test for Kruskal-type ranked data. **f**, Hepatic lipid analysis by ORO staining at 6 weeks (WT *n* = 9; and *Pex1*^G844D/G844D^ treated with vehicle *n* = 10; 1 × 10^11^ vg *n* = 9; 4 × 10^10^ vg *n* = 5; 1 × 10^10^ vg *n* = 7) and 16 weeks (WT *n* = 10; and *Pex1*^G844D/G844D^ treated with vehicle *n* = 10; 1 × 10^11^ vg *n* = 7; 4 × 10^10^ vg *n* = 8; 1 × 10^10^ vg *n* = 9). The percentage of stained area is shown. Images captured at 20× magnification; scale bar, 50 μm. Dots represent individual mice in **c**, **d** and **f**, and individual lipids in **e**. Bars indicate mean ± s.d. (**c**) or s.e.m. (**d**,**f**). Statistical analyses used two-way analysis of variance (ANOVA) with Sidak’s multiple-comparison test unless otherwise noted, with significance set at *P* < 0.05.

To assess the relative contributions of viral dose or editor cargo to toxicity, we injected P_1_ *Pex1*^G844D/G844D^ mice with a 4 × 10^11^ vg dose of AAV9-GFP. AAV9-GFP-treated *Pex1*^G844D/G844D^ mice survived at a similar rate (~20%; Extended Data Fig. 2a) compared with *Pex1*^G844D/G844D^ mice treated with AAV9-ABE at the same dose (~30%; Extended Data Fig. 1b). These results suggest that a high viral dose is the major contributor to the observed toxicity, rather than the ABE8e-V106W cargo. Because the 4 × 10^11^ vg dose of AAV9-ABE8e-V106W achieved high editing but reduced survival, we lowered the AAV9-ABE dose to identify a safe yet effective level. We performed facial vein injection at P_1_ of 1 × 10^11^ vg, 4 × 10^10^ vg or 1 × 10^10^ vg (Extended Data Fig. 2b). These doses were chosen based on the dose-dependent editing that we observed in the heterozygous cohort. After injection, all three dosing conditions were well tolerated (more than 90% of treated mice survived) (Fig. 2a). The growth deficit in the *Pex1*^G844D/G844D^ mice was effectively rescued at all doses of ABE8e-V106W in both males and females (Fig. 2b). In addition, high-throughput sequencing (HTS) analysis at 6 weeks of age showed 56% and 57% average correction in the bulk liver of the *Pex1*-p.G844D allele at the 1 × 10^11^ vg and 4 × 10^10^ vg doses, respectively, with average indel frequencies below 0.12%. Although the 1 × 10^10^ vg dose yielded 29% editing of pathogenic alleles at week 6, the frequency of corrected alleles increased 1.8-fold by week 16, resulting in an average of 53% editing efficiency, similar to what we observed in mice treated with the 1 × 10^11^ vg and 4 × 10^10^ vg doses (Fig. 2c). Higher doses led to faster correction, consistent with the durable expression of ABE (Extended Data Fig. 2c) from AAV among transduced cells. We also assessed base editing of the *Pex1*-p.G844D variant in central nervous system tissues such as cortex, cerebellum and retina 16 weeks post-injection. As expected^37,39^, we observed low editing efficiency of 0.35%, 0.14% and 0.51%, respectively, likely owing to the inefficiency of AAV9 transduction of mouse central nervous system (CNS) following systemic administration^51^ (Extended Data Fig. 3a–c).

### ABE rescues liver pathologies and peroxisome metabolites in homozygous *Pex1*-p.G844D mice

To assess the impact of gene editing on peroxisome homeostasis, we measured ABCD3 (also called PMP70) peroxisomal membrane protein levels, a previously reported biomarker of hepatic peroxisome content^52^ that can be influenced by biogenesis or pexophagy activity. Consistent with the known peroxisome deficit in this mouse model, western blot analysis revealed little to no detected ABCD3 protein in *Pex1*^G844D/G844D^ vehicle-treated mouse liver tissues (Fig. 2d). By contrast, liver tissue from ABE8e-V106W-treated *Pex1*^G844D/G844D^ mice revealed a dramatic increase in ABCD3 protein levels at all tested doses (Fig. 2d). ABCD3 protein levels at all doses were similar to those in WT mice, suggesting that even the lowest tested dose (1 × 10^10^ vg) of AAV encoding ABE8e-V106W could begin to restore peroxisome homeostasis.

Next, we analysed levels of free and esterified fatty acids in plasma and liver and found a dose-dependent rescue of hepatic peroxisomal metabolic activities in *Pex1*^G844D/G844D^ mice at early time points (6 weeks after injection). The 1 × 10^11^ vg and 4 × 10^10^ vg ABE doses normalized increased phytanic and pristanic acid (BCFA) in liver and plasma in *Pex1*^G844D/G844D^ mice, consistent with rescue of peroxisomal alpha- and beta-oxidation activity, while the lowest dose tested (1 × 10^10^ vg) resulted in a partial reduction (Fig. 2e and Extended Data Fig. 3d). Likewise, at 6 weeks, the 1 × 10^11^ vg and 4 × 10^10^ vg doses resulted in near-complete normalization of increased hepatic VLCFA (including C26:0 and C26:1 n-9) levels in *Pex1*^G844D/G844D^ mice, consistent with rescue of the hepatic peroxisomal beta-oxidation pathway, while the lowest dose tested resulted in partial reduction (Fig. 2e). Increased hepatic levels of the membrane lipid C26:0-LPC in *Pex1*^G844D/G844D^ mice were unchanged after ABE-AAV9 treatment, suggesting heterogeneity in the rates of metabolism of specific C26:0-containing lipids (Supplementary Note 2). At 6 weeks, increased plasma C26:1 n-9 levels were partially reduced at the 1 × 10^11^ vg and 4 × 10^10^ vg doses, but increased plasma C26:0 levels were unchanged. Increased plasma C26:0-LPC levels were not reduced in treated *Pex1*^G844D/G844D^ mice (Extended Data Fig. 3d and Supplementary Note 2).

At 16 weeks after injection, more robust rescue of hepatic peroxisome lipid metabolism in *Pex1*^G844D/G844D^ mice was observed. Phytanic and pristanic (BCFA) levels in liver and plasma (Fig. 2e and Extended Data Fig. 3d) were lowered to WT levels for all doses tested. Hepatic VLCFA levels in *Pex1*^G844D/G844D^ mice were near WT for all doses. Plasma C26:1 n-9 levels in *Pex1*^G844D/G844D^ mice were substantially reduced for all doses tested, but plasma C26:0 levels were mildly reduced. No reduction in liver or plasma C26:0-LPC levels was observed for any dose tested in these mice (Extended Data Fig. 3d and Supplementary Note 2). Overall, although varying the ABE-AAV dose changes the kinetics of gene correction, the major outcome after 16 weeks—robust correction of free and esterified BCFA and VLCFA levels in liver—were achieved across all doses tested.

Histological analysis of lipid content in liver sections also revealed a dose-dependent efficacy at an early age. The liver tissue in the low-dose-treated neonatal mice was characterized by irregular hepatic Oil Red O (ORO) staining (Fig. 2f, top right). By contrast, by 16 weeks after injection, hepatic lipid content was reduced to near-WT levels (Fig. 2f, bottom right). These results demonstrate that ABE8e-V106W-mediated correction of pathogenic *Pex1*-p.G844D alleles rescues clinically relevant peroxisomal liver disease phenotypes when delivered to neonatal mice at all tested doses.

### Transcriptome analysis on ABE8e-V106W-treated *Pex1*^G844D/G844D^ neonatal mice

**Extended Data Fig. 4 |:**
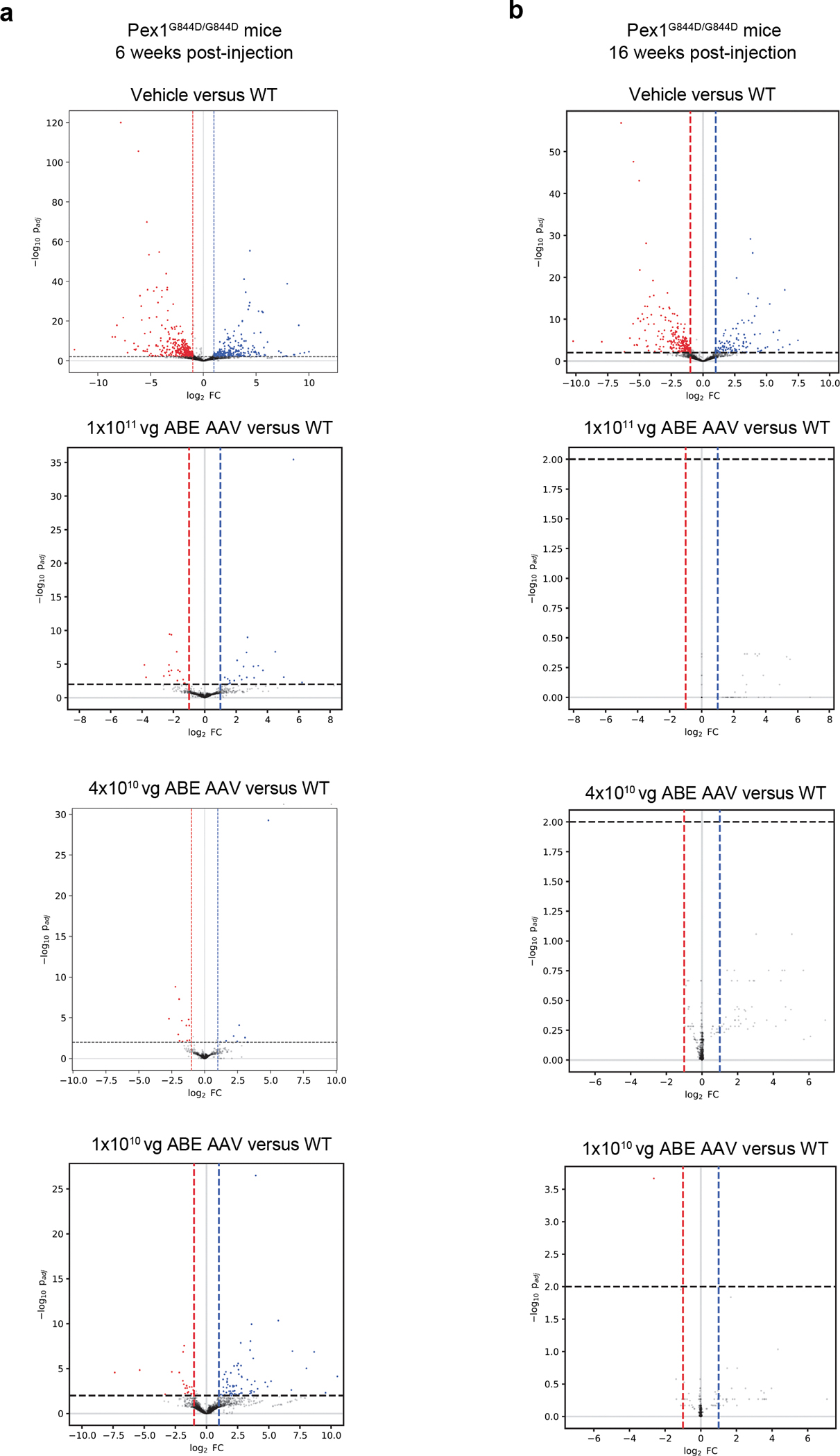
Analysis of differentially expressed genes in vehicle- or AAV9-ABE8e-V106W-treated *Pex1*^G844D/G844D^ neonatal mice compared to WT controls, related to Fig. 3. **a**, RNA-seq was performed and analyzed as indicated in the Methods. A false discovery rate (FDR) < 1% was applied to identify differentially expressed genes (DEGs) among vehicle-treated WT mice, vehicle-treated *Pex1*^G844D/G844D^ mice, and AAV9-ABE8e-V106W-treated *Pex1*^G844D/G844D^ mice. Volcano plots display DEGs for the following comparisons (from top to bottom): *Pex1*^G844D/G844D^ vehicle versus WT vehicle; *Pex1*^G844D/G844D^ treated with 1×10^11^ vg AAV9-ABE versus WT vehicle; *Pex1*^G844D/G844D^ treated with 4×10^10^ vg AAV9-ABE versus WT vehicle; and *Pex1*^G844D/G844D^ treated with 1×10^10^ vg AAV9-ABE versus WT vehicle. Cutoff thresholds are indicated by black dashed lines. RNA-seq was performed using liver tissue samples: vehicle-treated WT mice (6 weeks n = 4; 16 weeks n = 3), vehicle-treated *Pex1*^G844D/G844D^ mice (6 weeks n = 3; 16 weeks n = 4), and AAV9-ABE8e-V106W treated *Pex1*^G844D/G844D^ mice (1×10^11^ vg: 6 weeks n = 4, 16 weeks n = 4; 4×10^10^ vg: 6 weeks n = 4, 16 weeks n = 4; 1×10^10^ vg: 6 weeks n = 7, 16 weeks n = 4). Analyses of samples collected at 6 weeks post-injection are shown. **b**, Samples at 16 weeks post injection were analyzed and plotted as in **a**.

To further understand the mechanisms underlying the phenotypic correction in mice treated with AAV9-ABE8e-V106W, we performed RNA sequencing (RNA-seq) analysis of liver tissues from mice injected with 1 × 10^11^ vg, 4 × 10^10^ vg or 1 × 10^10^ vg at 6 and 16 weeks after injection (Supplementary Table 1). We applied a stringent false discovery rate (FDR < 1%) to identify differentially expressed genes (DEGs) among different treatment conditions. At 6 weeks, 691 DEGs were identified in livers from the homozygous *Pex1*^G844D/G844D^ and WT mice. Even at this early time point, there were dramatic decreases in the numbers of DEGs in the base-edited *Pex1*^G844D/G844D^ compared with saline-treated WT mice (105 DEGs at the 1 × 10^10^ vg dose, 13 at the 4 × 10^10^ vg dose and 38 at the 1 × 10^11^ vg dose) (Extended Data Fig. 4a and Supplementary Table 2). By the 16-week time point, a total of 352 DEGs were identified in livers from the *Pex1*^G844D/G844D^ and WT mice. Remarkably, there were no DEGs detected between livers of *Pex1*^G844D/G844D^ mice treated with the ABE8e-V106W at the 4 × 10^10^ vg and 1 × 10^11^ vg doses and WT mice at 16 weeks. Only one DEG was detected in the liver of the *Pex1*^G844D/G844D^ mice treated with the ABE8e-V106W at the lower 1 × 10^10^ dose (Extended Data Fig. 4b and Supplementary Table 2). These data indicate that in vivo base editing correction of *Pex1*-p.G844D normalizes the liver transcriptomic profile 16 weeks after treatment.

**Fig. 3 |:**
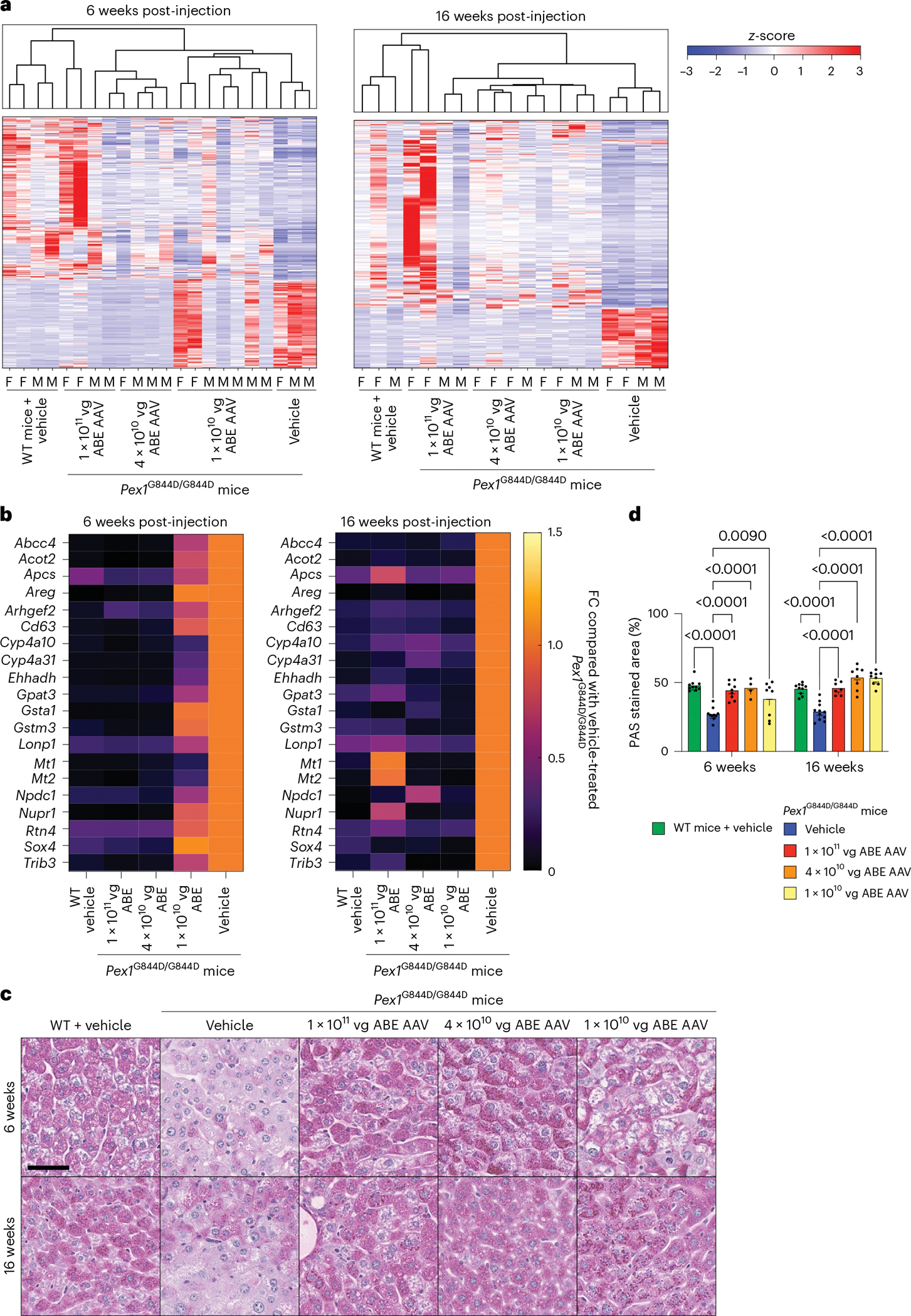
Transcriptome and pathway analysis of ABE8e-V106W-treated *Pex1*^G844D/G844D^ neonatal mouse liver. **a**, Hierarchical clustering of DEGs among vehicle-treated WT mice (6 weeks *n* = 4; 16 weeks *n* = 3), vehicle-treated *Pex1*^G844D/G844D^ mice (6 weeks *n* = 3; 16 weeks *n* = 4) and AAV9-ABE8e-V106W-treated *Pex1*^G844D/G844D^ mice (1 × 10^11^ vg, 6 weeks *n* = 4, 16 weeks *n* = 4; 4 × 10^10^ vg, 6 weeks *n* = 4, 16 weeks *n* = 4; 1 × 10^10^ vg, 6 weeks *n* = 7, 16 weeks *n* = 4). Liver samples were collected at 6 and 16 weeks post-injection. F, female; M, male. **b**, Heat map showing FC for 20 (*Abcc4*, *Acot2*, *Apcs*, *Areg*, *Arhgef2*, *Cd63*, *Cyp4a10*, *Cyp4a31*, *Ehhadh*, *Gpat3*, *Gsta1*, *Gstm3*, *Lonp1*, *Mt1*, *Mt2*, *Npdc1*, *Nupr1*, *Rtn4*, *Sox4* and *Trib3*) DEGs associated with liver injury, fibrosis and carcinoma. FC was calculated as mean expression for each condition divided by that in vehicle-treated *Pex1*^G844D/G844D^ mice. Conditions analysed include vehicle-treated WT mice, vehicle-treated *Pex1*^G844D/G844D^ mice and AAV9-ABE8e-V106W-treated *Pex1*^G844D/G844D^ mice (1 × 10^11^ vg, 4 × 10^10^ vg and 1 × 10^10^ vg). Adjusted *P* values from statistical tests (PyDESeq2) for all the comparisons are provided in Source Data Fig. 3b. **c**, Histological analysis of glycogen in liver sections by PAS staining at 6 and 16 weeks in vehicle-treated WT mice (*n* = 10), vehicle-treated *Pex1*^G844D/G844D^ mice (6 weeks *n* = 10; 16 weeks *n* = 11) and ABE8e-V106W-treated *Pex1*^G844D/G844D^ mice (1 × 10^11^ vg, 6 weeks *n* = 9, 16 weeks *n* = 7; 4 × 10^10^ vg, 6 weeks *n* = 4, 16 weeks *n* = 8; 1 × 10^10^ vg, 6 weeks *n* = 8, 16 weeks *n* = 9). **d**, Quantification of PAS staining as percentage of stained area averaged over three random regions of interest per section (images captured at 10× magnification). Representative images captured at 20× magnification; scale bar, 50 μm. Data points represent individual mice (**a**–**d**). In **d**, error bars represent the s.e.m. Numbers above bars indicate *P* values for specified comparisons. Statistical analysis by two-way ANOVA with Sidack’s multiple-comparison test unless otherwise noted; significance set at *P* < 0.05.

To further explore, we performed hierarchical clustering analysis on all DEGs from ABE-treated versus vehicle-treated *Pex1*^G844D/G844D^ mouse livers at 6 weeks (363 genes) and 16 weeks (250 genes) after injection (Fig. 3a and Supplementary Table 3). The lowest AAV9-ABE dose, 1 × 10^10^ vg (equivalent to 7 × 10^12^ vg kg^−1^), resulted in a partially rescued transcriptome state between WT and homozygous disease mice at 6 weeks after injection. In comparison, by 16 weeks, the mice showed near-complete reversal of transcriptome changes, suggesting that the increased correction of *Pex1*-G844D pathogenic variants from 29% to 53% during this time contributed to transcriptome normalization.

**Extended Data Fig. 5 |:**
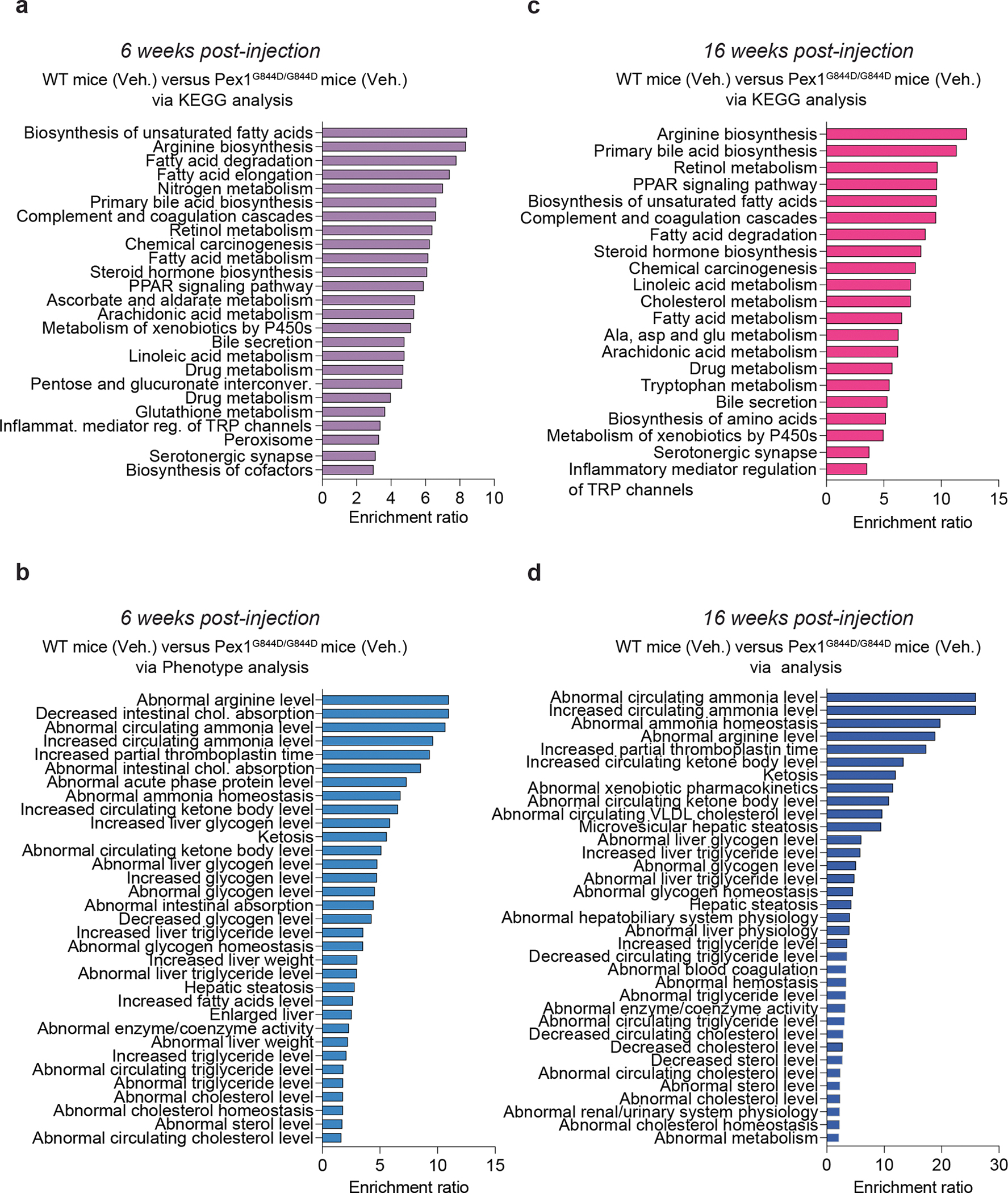
KEGG pathway and phenotype analyses of differentially expressed genes in treated neonatal mouse liver tissues. **a**, Differentially expressed genes between vehicle-treated WT and vehicle-treated *Pex1*^G844D/G844D^ mice at 6 weeks post-injection were analyzed using WebGestalt for gene set analysis. All KEGG categories with FDR < 0.01 (Benjamini-Hochberg method) are shown. Categories were restricted to those defined by 10–1000 genes, to exclude overly specialized (< 10 genes) or overly broad (> 1000 genes) sets. **b, d** Phenotype enrichment analyses, with enrichment ratio indicated on the x-axis. **c**, Differentially expressed genes between vehicle-treated WT and vehicle-treated *Pex1*^G844D/G844D^ mice at 16 weeks post injection were analyzed using the same criteria as in **a**. KEGG pathway analysis is shown (The number of tissue samples is the same as in Extended Data Fig. 4).

Gene-set-based KEGG and phenotype pathway analysis of DEGs between livers of saline-treated *Pex1*^G844D/G844D^ and WT mice at 6 and 16 weeks revealed DEGs related to physiological responses to liver injury, fibrosis and hepatocellular carcinoma (Supplementary Table 4). Twenty severe liver disease-associated DEGs were more highly expressed in *Pex1*^G844D/G844D^ mice compared with WT controls (Fig. 3b). Consistent with a rescue of liver health, these genes were downregulated to at or near-WT levels in the ABE-treated *Pex1*^G844D/G844D^ mice (Fig. 3b). In addition, alterations in hepatic carbohydrate metabolism have been reported in mouse models with ZSD and *Drosophila* models with other peroxisomal biogenesis disorders^39,53^. As expected^37,39^, many DEGs found between mutant and WT mice linked to peroxisomal/mitochondrial lipid metabolic dysfunctions and carbohydrate metabolism were rescued upon ABE treatment (Fig. 3a, Extended Data Fig. 5 and Supplementary Table 5).

Furthermore, histologic analysis of liver tissues via periodic acid–Schiff (PAS) staining revealed restoration of normal glycogen levels upon ABE treatment (Fig. 3c,d), supporting the conclusion that phenotypic rescue is the result of ABE-mediated correction. We found that *Cyp4a10*, *Cyp4a14* and *Cyp4a31*—genes associated with liver ω-oxidation^54–56^—were more highly expressed in *Pex1*^G844D/G844D^ compared with WT mice, potentially reflecting PPAR-mediated activation of a compensatory mechanism to catabolize BCFAs. These same genes were downregulated upon ABE treatment at 16 weeks after injection compared with the vehicle-treated *Pex1*^G844D/G844D^ mice, suggesting that ABE-enabled rescue minimized dependence on the compensatory ω-oxidation pathway owing to the restoration of peroxisomal α-oxidation activity. Together, the transcriptome analyses above support therapeutic benefit of AAV9-ABE-V106W for rescuing liver pathogenicity in neonatal *Pex1*^G844D/G844D^ mice.

### Therapeutic base editing in 4-week-old *Pex1*^G844D/G844D^ mice

**Extended Data Fig. 6 |:**
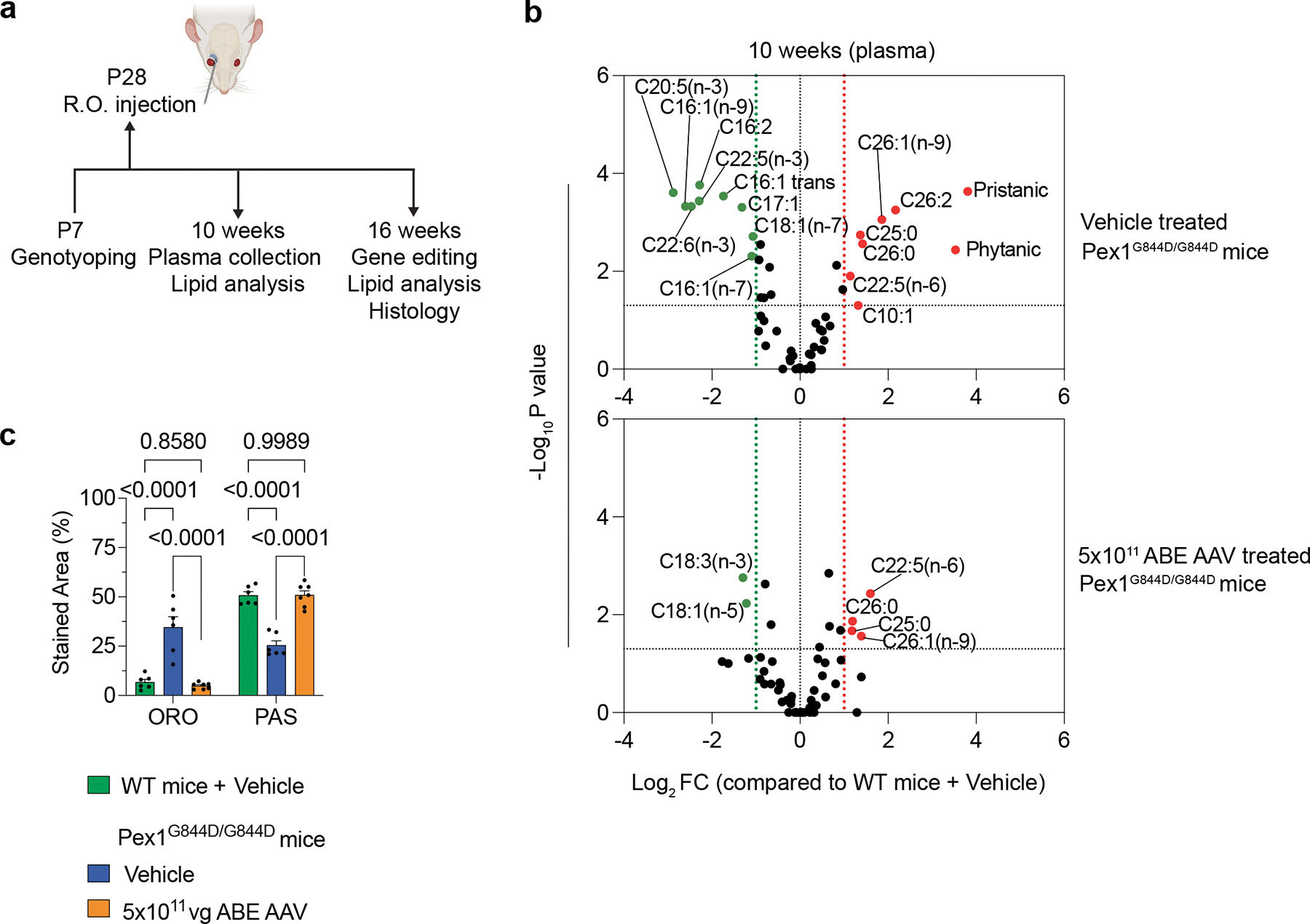
Analysis of ABE-AAV-treated 4-week-old *Pex1*^G844D/G844D^ mice, related to Fig. 4. **a**, Schematic of retro-orbital injection of 4-week-old *Pex1*^G844D/G844D^ mice (n = 7) with AAV9-ABE8e-V106W (5×10^11^ vg per mouse) to correct the *Pex1*-p.G844D pathogenic allele. WT (n = 6) and *Pex1*^G844D/G844D^ (n = 5) injected with vehicle, served as controls. Mice randomized from multiple litters (mixed sex). **b**, Lipid profile of plasma at 10 weeks by GC-MS; volcano plots show fold-change relative to WT. Green and red dotted lines indicates twofold decrease and increase; black dashed line marks P = 0.05. P values calculated using Dunn’s non-parametric many-to-one comparison test for Kruskal-type ranked data. **c**, Histological analysis of hepatic lipid content by Oil Red O (ORO) staining and glycogen content by Periodic Acid Schiff (PAS) staining at 16 weeks in vehicle-treated WT mice, vehicle-treated *Pex1*^G844D/G844D^ mice, and ABE8e-V106W-treated *Pex1*^G844D/G844D^ with 5×10^11^dew per mouse. Bars represent mean ± s.e.m.; dots indicate individual mice, n as in a. Mouse diagram in **a** created in BioRender; Piec, M. https://biorender.com/l4insv3 (2026).

**Fig. 4 |:**
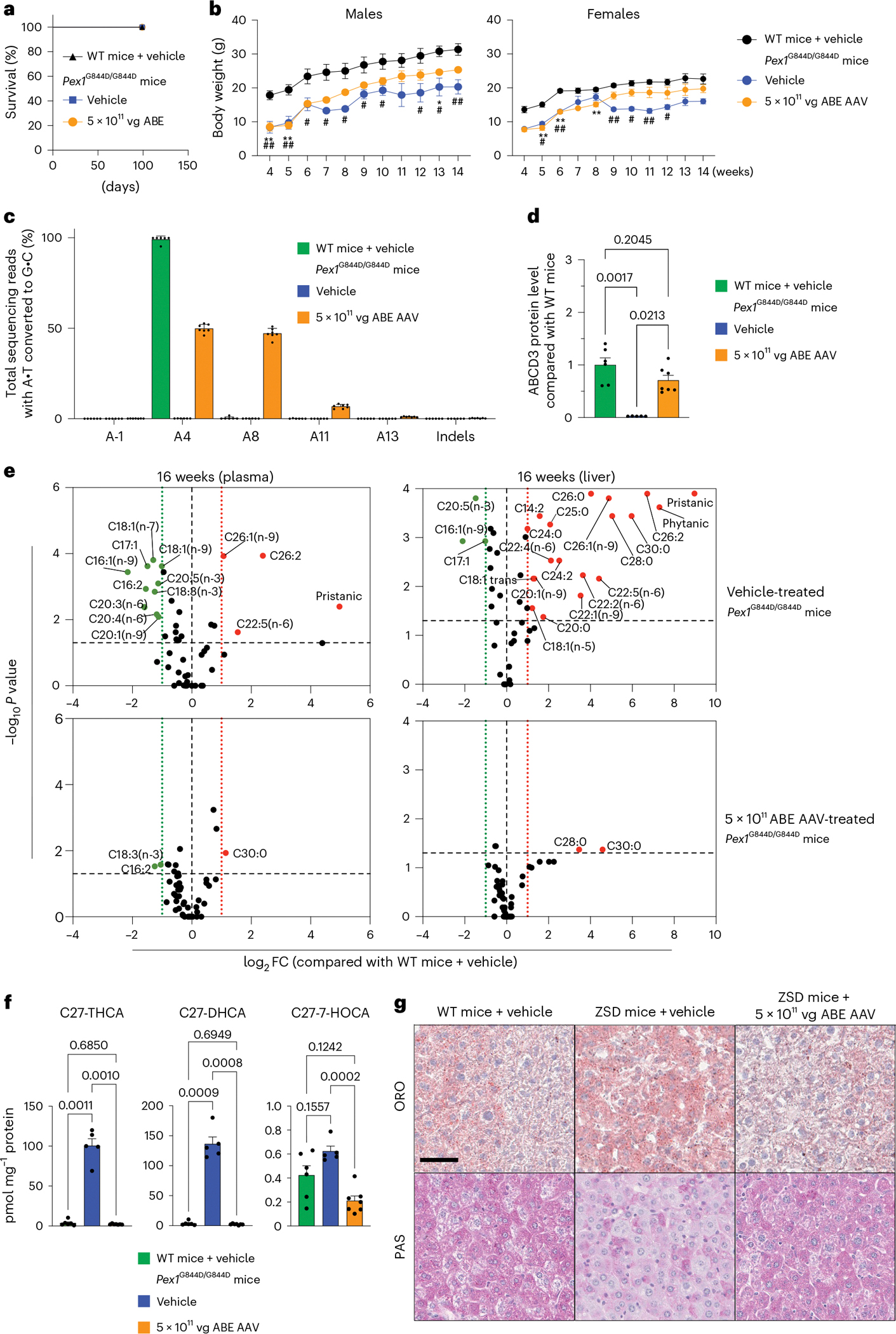
Therapeutic base editing in young *Pex1*^G844D/G844D^ mice. **a**, Kaplan–Meier survival curves following injection of 4-week-old *Pex1*^G844D/G844D^ mice (*n* = 7; 4 females, 3 males) with AAV9-ABE8e-V106W (5 × 10^11^ vg per mouse) to correct the *Pex1*-p.G844D pathogenic allele and WT (*n* = 6; 3 females, 3 males) and *Pex1*^G844D/G844D^ (*n* = 6, 3 females, 3 males) with vehicle served as controls. **b**, Growth curves for females and males (*n* as in **a**). Statistical analysis by two-way ANOVA and Sidak’s multiple-comparison test: *P < 0.05, **P < 0.01 for AAV9-ABE8e-V106W-treated *Pex1*^G844D/G844D^ mice versus WT; #*P* < 0.05, ##*P* < 0.01 for vehicle-treated *Pex1*^G844D/G844D^ versus WT. Gel diet supplementation provided during neonatal stage, removed at 8 weeks (males) and 6 weeks (females). **c**, Editing efficiencies in liver by HTS and CRISPResso2 analysis; edited adenines within and near the protospacer are shown (*n* as in **a**). **d**, Peroxisome assembly assessed by automated western blot for ABCD3 at 16 weeks in liver of WT vehicle-treated (*n* = 6) or *Pex1*^G844D/G844D^ mice treated with ABE-AAV (*n* = 7) or vehicle (*n* = 5). **e**, Plasma and liver lipid profiles of at 16 weeks by GC-MS; volcano plots show FC relative to WT. Green and red dotted lines indicate twofold decrease and increase; black dashed line marks *P* = 0.05. *P* values calculated using Dunn’s nonparametric many-to-one comparison test for Kruskal-type ranked data (*n* as in **a**). **f**, Abundance of bile acid intermediates (THCA, DHCA and 7-HOCA) in liver of AAV9-ABE8e-V106W-treated *Pex1*^G844D/G844D^ mice (*n* = 7) and controls WT (*n* = 6) and *Pex1*^G844D/G844D^ (*n* = 5) vehicle-treated. **g**, Histological analysis of hepatic glycogen (PAS) and lipid (ORO) at 16 weeks. Images captured at 20× magnification; scale bar, 50 μm. Dots represent individual mice in **c**, **d** and **f**, individual lipids in **e** and mean body weight in **b**. Bars indicate the mean; error bars represent s.e.m. Statistical analysis (**d**,**f**) by two-way ANOVA with Sidak’s multiple-comparison test; significance set at *P* < 0.05.

While neonatal treatment with ABE8e-V106W resulted in an almost complete rescue of the ZSD hepatic phenotypes, testing the effect of in vivo base editing in animals at a more advanced disease stage would inform the potential of treatment for patients of different ages. Therefore, we injected AAV9-ABE8e-V106W at a dose of 5 × 10^11^ vg per mouse (equivalent to the neonatal dose of 1 × 10^11^ vg per mouse or 7 × 10^13^ vg kg^−1^) via retro-orbital injection into 4-week-old *Pex1*^G844D/G844D^ mice (Extended Data Fig. 6a). We tested the lipid profile in plasma at 6 and 12 weeks after injection and in the liver at 12 weeks after injection. The treatment was well tolerated, and all animals survived until the 16-week endpoint (Fig. 4a). The body weight of *Pex1*^G844D/G844D^ mice treated with ABE8e-V106W improved compared with vehicle-treated controls from 8 weeks of age onwards (Fig. 4b). We assessed base editing efficiency at 16 weeks of age, revealing 50% bulk liver correction of the *Pex1*-p.G844D allele with no detected non-synonymous bystander editing (Fig. 4c). ABCD3 protein levels were restored in base-edited mice to levels similar to WT controls, consistent with improved peroxisome function (Fig. 4d). The levels of phytanic and pristanic BCFAs and most VLCFAs in plasma and liver were also restored to those comparable to WT controls (Fig. 4e and Extended Data Fig. 6b).

Consistent with the cholestatic liver disease present in people with moderate and severe ZSD, by 16 weeks *Pex1*^G844D/G844D^ mice showed an accumulation of C27-bile acid intermediates (3α,7α-dihydroxycholestanoic acid (DHCA) and 3α,7α,12α-trihydroxycholestanoic acid (THCA)) thought to be involved in its aetiology^16^ (Fig. 4f). To determine whether restoration of *PEX1* activity could address the accumulation of these C27-bile acid intermediates, we analysed DHCA, THCA and 7α-hydroxy-3-oxo-4-cholestenoic acid (7-HOCA) in the liver and found that they were restored to WT levels, reflecting the rescue of a biosynthetic pathway requiring peroxis omal beta-oxidation (Fig. 4f). Mature bile acid levels did not differ significantly in WT and mutant mice or in mice treated with the ABE (Supplementary Note 3). Histologic analysis of the liver suggested rescue of hepatic lipids and carbohydrate metabolism (Fig. 4g and Extended Data Fig. 6c). These findings indicate that base editing resulted in physiologically relevant rescue of liver function when administered to 4-week-old *Pex1*^G844D/G844D^ mice.

**Extended Data Fig. 7 |:**
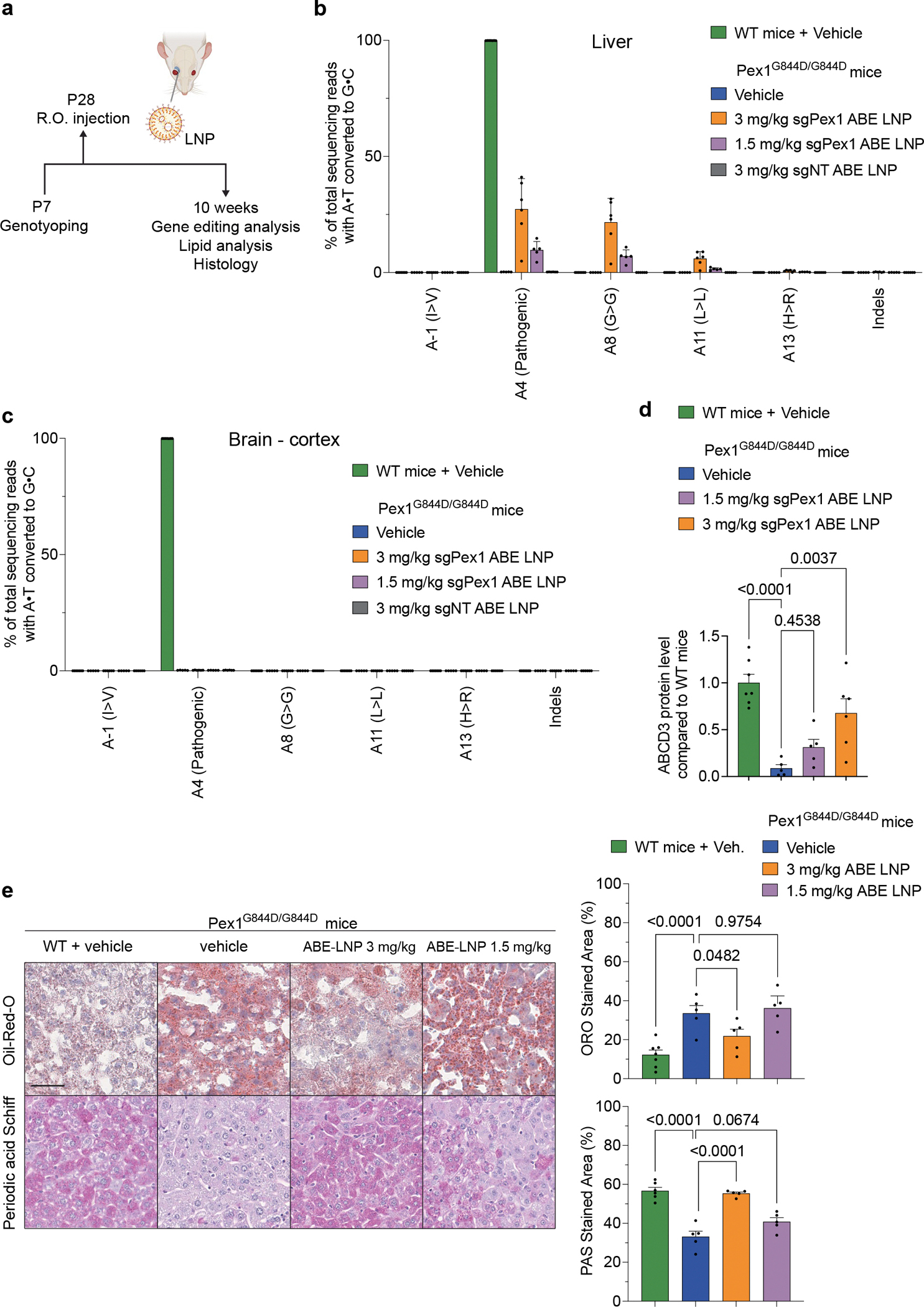
Analysis of ABE-LNP-treated 4-week-old mice. **a**, Schematic of retro-orbital (RO) injection of ABE-LNP into 4-week-old mice. **b**, Base editing efficiencies in liver quantified via HTS and CRISPResso2^99^ analysis in vehicle-treated mice (WT n = 7; *Pex1*^G844D/G844D^ n = 5), sgPex1-ABE-LNP-treated *Pex1*^G844D/G844D^ mice (3 mg/kg n = 6; 1.5 mg/kg n = 5) or sgNT-ABE-LNP-treated mice (non-targeting guide, 3 mg/kg n = 6). Adenines within and near the protospacer are shown. **c**, Base editing efficiencies in brain cortex tissue (n as in **b**). Bars represent the mean ± s.d.; dots indicate individual mice (**b**, **c**). **d**, Automated Western blot analysis of ABCD3 levels in liver homogenates from vehicle- or ABE-LNP-treated mice (1.5 mg/kg or 3 mg/kg; n as in **b**). Peak area for ABCD3 was normalized to total protein, and fold-change was calculated relative to WT. Statistical analysis was performed by two-way ANOVA and Sidak’s multiple comparison test. **e**, Histological analysis of hepatic lipid content by Oil Red O (ORO) staining and glycogen content by Periodic Acid Schiff (PAS) staining. Representative liver images at 10 weeks of age for vehicle-treated WT mice (n = 7), *Pex1*^G844D/G844D^ mice (n = 5), or ABE8e-V106W-treated *Pex1*^G844D/G844D^ mice injected with 3 mg/kg (n = 5) or 1.5 mg/kg (n = 5) LNP per mouse. Images captured at 20x magnification; scale bar, 50 μm. Bars represent mean ± s.e.m.; dots indicate individual mice (**d, e**). Statistical analysis was performed using two-way ANOVA and Sidak’s multiple comparison test. P-values above bars indicate comparisons using the statistical method specified above. Mouse and LNP diagram in **a** created in BioRender; Piec, M. https://biorender.com/1kdiodh (2026).

**Extended Data Fig. 8 |:**
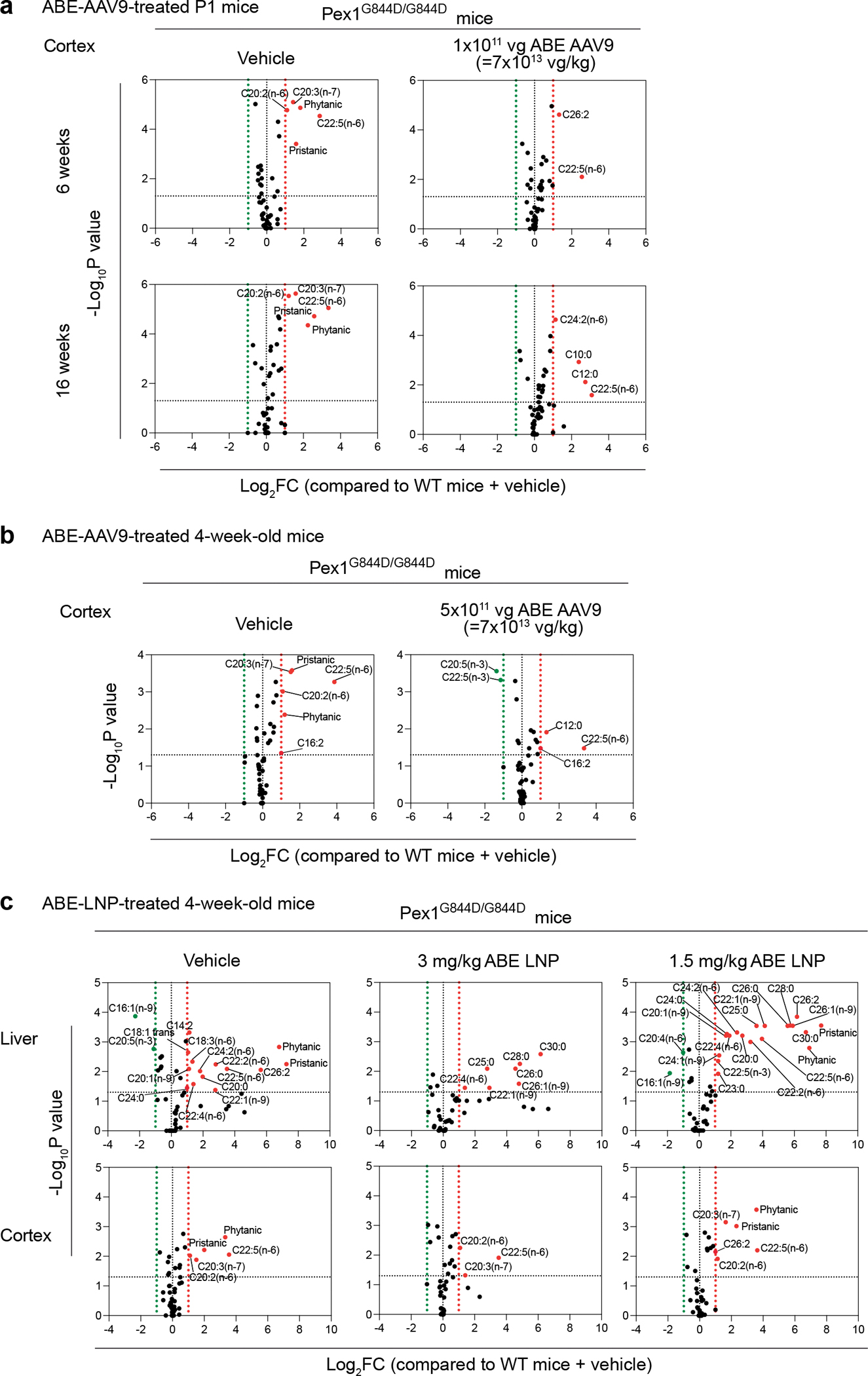
Lipid profiles in ABE-AAV9-treated or ABE-LNP-treated mouse brain cortex and liver tissues. **a**, Lipid profile of ABE-AAV9-treated P1 neonatal mice based on GC-MS analysis of brain cortex samples collected at 6 and 16 weeks. Cortex of vehicle-treated WT mice (6 weeks n = 9, 16 weeks n = 10) Vehicle-treated *Pex1*^G844D/G844D^ mice (6 weeks n = 9, 16 weeks n = 11), AAV9-ABE8e-V106W-treated *Pex1*^G844D/G844D^ mice at doses 1×10^11^ vg (6 weeks n = 9, 16 weeks n = 7). **b**, Lipid profiles of ABE-AAV9-treated 4-week-old mice based on GC-MS analysis of brain cortex samples collected at 16 weeks. **c**, Lipid profiles of ABE-LNP-treated 4-week-old mice based on GC-MS analysis of liver and brain cortex samples collected at 16 weeks. Vehicle-treated WT mice (n = 6) Vehicle-treated *Pex1*^G844D/G844D^ mice (n = 6), AAV9-ABE8e-V106W-treated *Pex1*^G844D/G844D^ mice at 5×10^11^ vg (n = 7), **c**. Liver of vehicle-treated mice (WT n = 8; *Pex1*^G844D/G844D^ n = 5), sgPex1-ABE-LNP-treated *Pex1*^G844D/G844D^ mice (3 mg/kg n = 5; 1.5 mg/kg n = 4). Cortex of vehicle-treated mice (WT n = 8, *Pex1*^G844D/G844D^n = 5, sgPex1-ABE-LNP-treated *Pex1*^G844D/G844D^ mice (3 mg/kg n = 5; 1.5 mg/kg n = 5). For **a-c**, fold-change in signal for each lipid was calculated relative to the average for vehicle-treated WT mice. Volcano plots show fold-change in lipid abundance. Green and red dotted lines indicate twofold decrease and increase, respectively; black dashed line marks P = 0.05. P values were calculated relative to vehicle-treated WT samples using Dunn’s nonparametric many-to-one comparison test for Kruskal-type ranked data.

LNPs offer a more transient, non-viral delivery modality for gene editing agents. LNP-mediated base editor delivery has been used effectively in non-human primates and in human patients^57,58^. In addition to being amenable to well-established, clinically accepted manufacturing methods, the transience of LNP-mediated base editor mRNA delivery minimizes the potential for off-target editing after the targeted edits are made^59,60^. To test the feasibility of LNP-mediated ABE mRNA delivery in *Pex1*^G844D/G844D^ mice, we formulated OF-02 LNPs^61^ that have previously demonstrated robust and highly specific RNA delivery to the mouse liver following intravenous administration^61^ and delivered ABE8e-V106W mRNAs and modified sgRNA into 4-week-old mice (Extended Data Fig. 7a). Encouragingly, we observed, in the liver, up to 27% allele correction in a dose-dependent manner, with minimal unwanted bystander editing or indels (Extended Data Fig. 7b,c). Average ABCD3 protein levels increased 7.6-fold in *Pex1*^G844D/G844D^ mice treated with the highest dose of ABE-LNP (3 mg kg^−1^) compared with saline-treated mice, indicating improved peroxisome function (Extended Data Fig. 7d). ORO staining showed that increased hepatic lipids in the *Pex1*^G844D/G844D^ mice were reduced by 33% 6 weeks after treatment with 3 mg kg^−1^ ABE-LNP, approaching lipid levels in WT mice (Extended Data Fig. 7e). Furthermore, PAS staining revealed restoration of normal hepatic glycogen levels upon 3 mg kg^−1^ ABE-LNP treatment (Extended Data Fig. 7e). These findings demonstrate the compatibility of the base editing strategy developed in this work with clinically validated non-viral in vivo base editor delivery methods. Although systemic delivery of ABE8e-V106W resulted in low genome editing in the CNS of treated mice as expected, we observed a significant reduction in BCFAs (phytanic and pristanic acids), but not VLCFAs, in the cortex of AAV- and LNP-treated mice after injection (Extended Data Fig. 8). This observation suggests that restoring hepatic alpha-oxidation reduces the pool of circulating phytanic acid that would otherwise accumulate in the cortex.

Collectively, AAV9-ABE8e-V106W-mediated correction of the pathogenic *Pex1*-p.G844D allele in 4-week-old mice resulted in near-complete rescue at genomic, lipidomic and phenotypic levels. Importantly, our findings indicate that the therapeutic benefits of ABE-V106W observed in neonates can be extended to young mice with a more advanced stage of liver disease.

### In vivo and in vitro off-target analysis of ABE8e-V106W targeting *Pex1* and *PEX1* pathogenic variants

**Extended Data Fig. 9 |:**
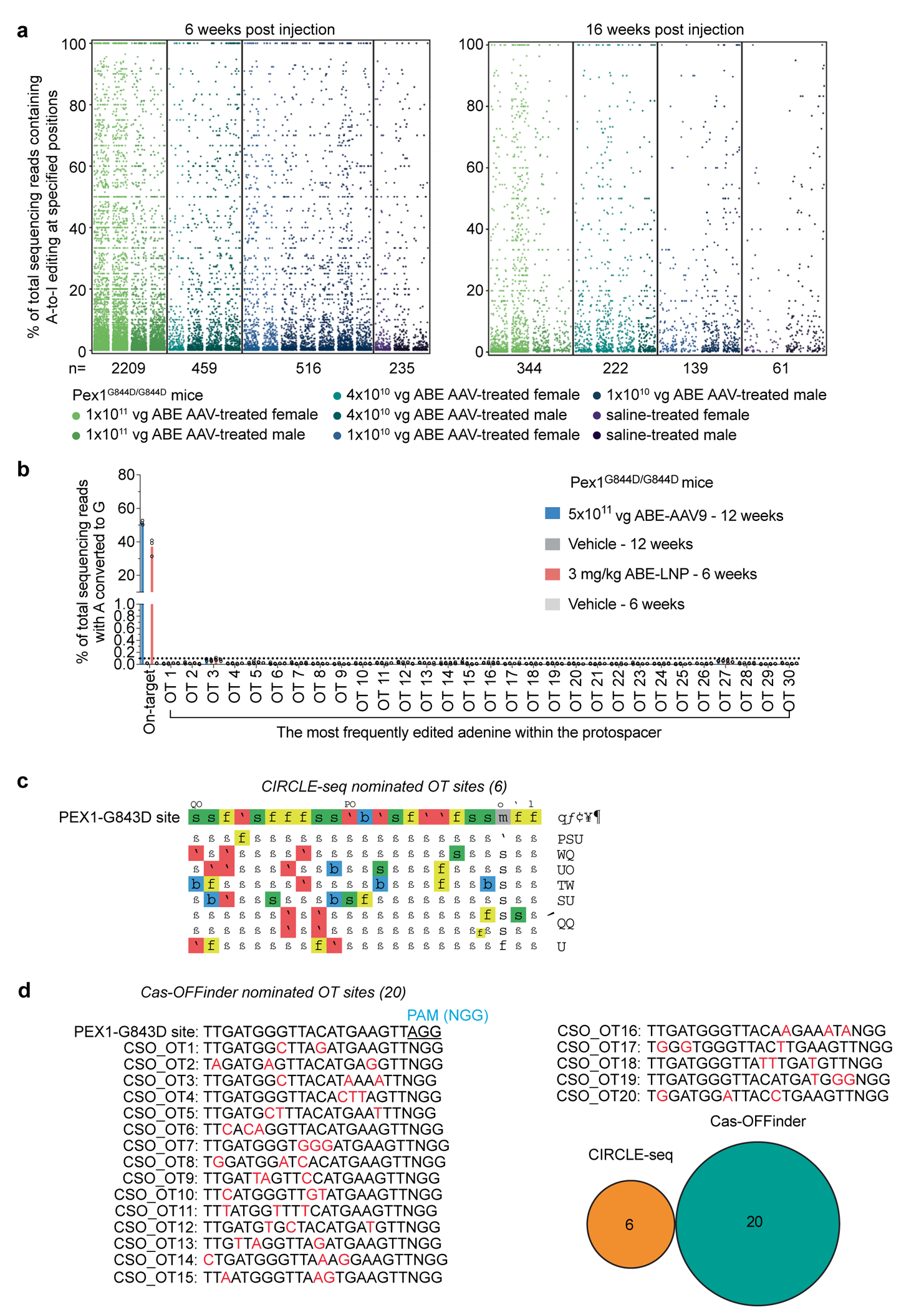
Off-target analyses of ABE8e-V106W-treated mouse tissues and human cells, related to Fig. 5. **a**, Jitter plots derived from RNA-seq experiments showing RNA adenines modified in ABE8e-V106W-AAV9-treated or saline-treated *Pex1*^G844D/G844D^ mouse liver tissues. Each dot represents an A-to-G change detected and n indicates the average of total number of modified adenines per liver sample at the same dose. **b**, Amplicon sequencing analysis of CIRCLE-seq-nominated off-target (OT) sites. The top 30 OT sites and *Pex1* on-target site were amplified and sequenced from liver genomic DNA (gDNA) of four-week-old *Pex1*^G844D/G844D^ mice treated AAV9-ABE8e-V106W (5×10^11^ vg), ABE-LNP, or vehicle. Editing efficiencies were measured by HTS and analyzed with CRISPResso2. n = 3 mice with the highest on-target editing efficiency were used. **c**, CIRCLE-seq analysis of ABE8e-V106W targeting the human *PEX1*-p.G843D pathogenic variant was performed using patient-derived fibroblast (*PEX1*^G843D/I700fs^) gDNA. Six potential OT editing sites were nominated. **d**, Schematic diagram showing nominated OT editing sites from CIRCLE-seq and Cas-OFFinder, including 20 potential OT sites nominated by Cas-OFFinder. Mismatched nucleotides are shown in red. No overlapping OT sites were observed between CIRCLE-seq- and Cas-OFFinder-nominated sites, as illustrated in the Venn diagram. Twenty-six potential OT sites were further examined by amplicon sequencing of liver gDNA from AAV9-ABE8e-V106W-treated mice (1X10^11^ vg).

**Fig. 5 |:**
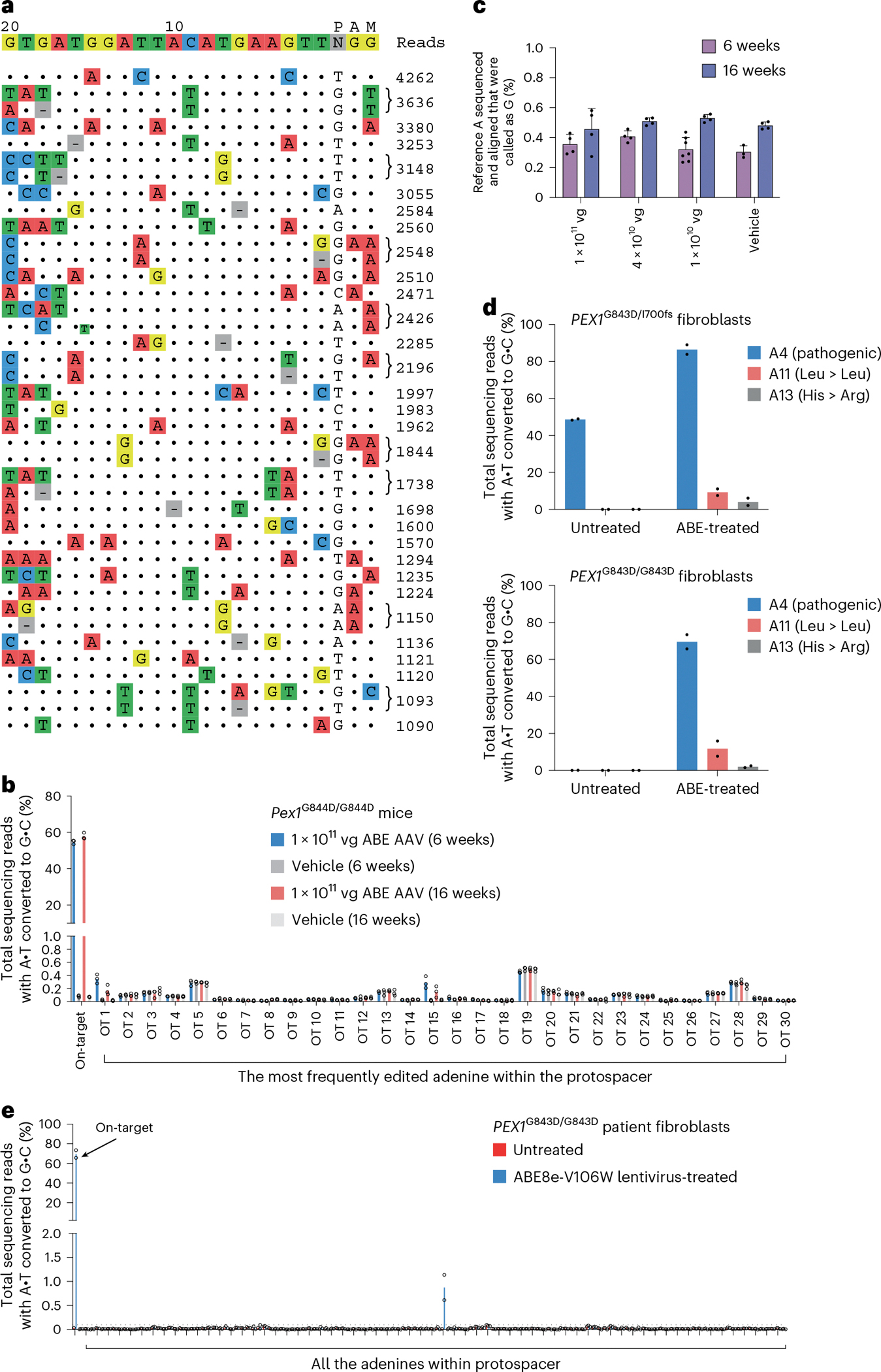
In vivo and in vitro off-target analysis of ABE8e-V106W targeting *Pex1*/*PEX1* pathogenic variants. **a**, Candidate off-target (OT) sites nominated by CIRCLE-seq using the *Pex1*-p.G844D-targeting gRNA, ranked by sequencing read count. **b**, Amplicon sequencing of the top 30 CIRCLE-seq-nominated OT sites at 6 and 16 weeks after treatment in liver genomic DNA from *Pex1*^G844D/G844D^ mice treated with AAV9-ABE8e-V106W (1 × 10^11^ vg) or vehicle (*n* = 3 per time point). Editing efficiencies were measured by HTS and analysed with CRISPResso2. **c**, Off-target RNA editing in liver transcriptomes at 6 and 16 weeks after treatment in *Pex1*^G844D/G844D^ mice treated with AAV9-ABE8e-V106W at 1 × 10^11^ vg (*n* = 4 per time point), 4 × 10^10^ vg (*n* = 4 per time point) or 1 × 10^10^ vg (6 weeks *n* = 7; 16 weeks *n* = 4), and vehicle-treated controls (6 weeks *n* = 3; 16 weeks *n* = 4). The percentage of total A-to-G changes in the transcriptome is shown. **d**, Correction of pathogenic *PEX1*-p.G843D variant in patient-derived fibroblasts following lentiviral delivery of ABE8e-V106W. Editing efficiencies were quantified by HTS and analysed with CRISPResso2. **e**, Amplicon sequencing of CIRCLE-seq and Cas-OFFinder-nominated OT sites in lentiviral ABE8e-V106W-transduced or untreated *PEX1*^G843D/G843D^ fibroblasts. The percentage of A•T-to-G•C changes for all the adenines within the protospacer is shown. Dots represent individual mice in **b** and **c**; in **d** and **e**, dots represent individual replicates. Bars indicate the mean allele frequency for the specified change. Two replicates were performed for **d** and **e**.

To assess Cas-dependent DNA and Cas-independent RNA off-target editing in ABE-V106W-treated mice, we performed CIRCLE-seq^62^, an unbiased and sensitive genome-wide off-target detection method that nominates genomic DNA sites engaged by SpCas9 nuclease complexed with the *Pex1*-p.G844D-targeting gRNA in vitro. When applied to mouse genomic DNA, CIRCLE-seq nominated 631 ranked candidate off-target sites. We evaluated the top 30 sites using the liver genomic DNA samples collected from the homozygous mice treated with 1 × 10^11^ vg of ABE-AAV at 6 weeks and 16 weeks after injection (Fig. 5a and Supplementary Table 6). Despite high dose of AAV9-ABE8e-V106W exposure, we observed minimal off-target editing, with only two sites showing editing above background levels from vehicle-treated controls: OT1 (≤0.35%) and OT15 (≤0.29%). Off-target editing did not increase between 6 and 16 weeks. Both off-target sites reside in intergenic regions and are not annotated as regulatory elements in the mouse genome. These data suggest a very low off-target editing rate obtained in the mice treated with even a high dose of AAV9-ABE8e-V106W (Fig. 5b). To identify Cas-independent RNA off-target editing in ABE-treated *Pex1*^G844D/G844D^ mice, we analysed liver RNA-seq datasets from mice treated with 1 × 10^11^ vg, 4 × 10^10^ vg or 1 × 10^10^ vg of ABE-AAV. At 6 weeks, but not 16 weeks after injection, we observed a modest 1.2-fold increase in transcriptome-wide A-to-G RNA changes relative to vehicle-treated controls (Fig. 5c and Extended Data Fig. 9a), consistent with higher AAV9-ABE8e-V106W expression level at earlier time points and with the transient nature of cellular RNA. These findings further support that ABE8e-V106W base editing of the *Pex1*-p.G844D allele is highly site specific, with minimal detected genetic or transcriptomic changes. In juvenile mice treated with ABE-AAV9 or ABE-LNPs, we likewise detected no significant increases in OT1 and OT15 editing relative to saline-treated controls. It is possible that differences in viral vector transduction efficiency between the neonatal and juvenile cohort may influence ABE expression levels and/or target site accessibility could vary with age (Extended Data Fig. 9b).

To identify potential off-target editing associated with the ABE8e-V106W base editor and a gRNA targeting human *PEX1*-p.G843D, we performed CIRCLE-seq analysis using patient-derived compound heterozygous *PEX1*^G843D/I700fs^ cultured skin fibroblasts, resulting in the nomination of six candidate off-target sites in total. In addition, we also performed in silico analysis, Cas-OFFinder^63^, and predicted 20 potential off-target sites with 3 or fewer mismatched nucleotides compared with the targeted protospacer (Extended Data Fig. 9c). To assess off-target editing associated with high and prolonged expression of ABE8e-V106W, we incubated two sets of patient-derived cultured fibroblasts (homozygous *PEX1*^G843D/G843D^ and compound heterozygous *PEX1*^G843D/I700fs^, respectively) with lentivirus to deliver ABE-V106W and gRNA. We confirmed efficient on-target editing with up to 73% correction of the target *PEX1*-p.G843D alleles 2 weeks after transduction (Fig. 5d). Next, we amplified the lentivirus-treated genomic DNA (gDNA) from *PEX1*^G843D/G843D^ cells at 26 nominated off-target sites (the 6 from CIRCLE-seq and 20 from Cas-OFFinder) and examined all possible A•T-to-G•C changes within the protospacers. We did not detect sequence modifications above the 0.1% detection threshold for HTS or above levels of genetic variation present in vehicle-treated samples for 25 of the 26 tested sites. One off-target site in an intron of *FBXO38* showed 0.88% A•T-to-G•C substitution, which could potentially affect a splicing acceptor sequence (Fig. 5e).

Overall, these analyses suggest that the ABE8e-V106W editing strategies both in vivo in mice and in vitro in patient-derived cells result in high-specificity editing, with minimal observed DNA or RNA off-target editing, even following a high-dose AAV treatment that leads to prolonged expression of ABE.

### Correction of *PEX1*-p.G843D variant in patient-derived fibroblasts restores peroxisome homeostasis

**Extended Data Fig. 10 |:**
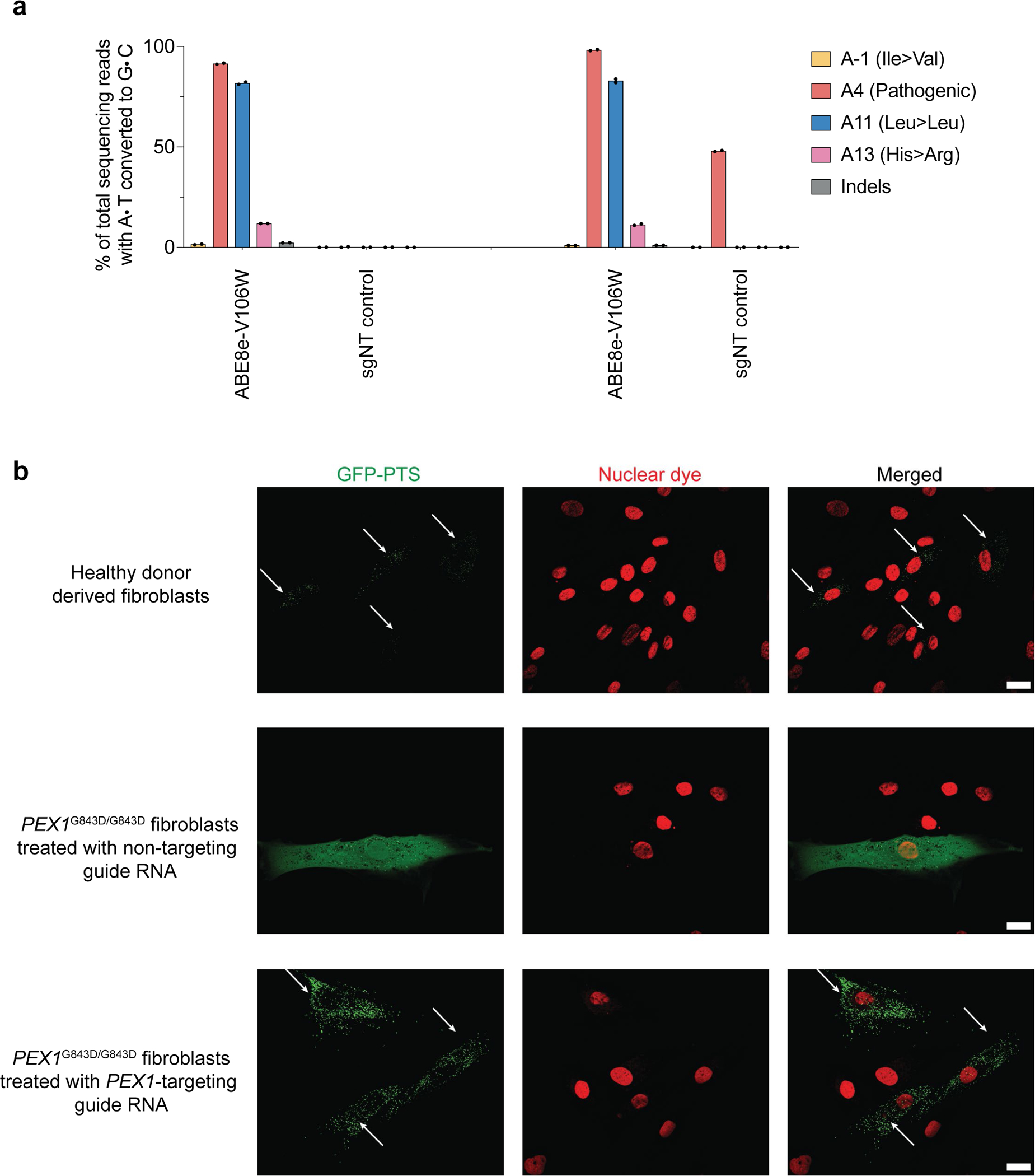
Assessment of ABE8e-V106W editing of patient-derived fibroblasts and live-cell imaging of peroxisomal assembly, related to Fig. 6. **a**, Two patient-derived fibroblast cell lines—homozygous *PEX1*^G843D/G843D^ and compound heterozygous *PEX1*^G843D/I700fs^—were nucleofected with ABE8e-V106W mRNA and a synthetic guide RNA targeting the *PEX1*-p.G843D (c.2528 G > A) pathogenic variant or a non-targeting control guide RNA (sgNT) that does not match any human genome sequence. Editing efficiencies were measured 72 h post-nucleofection by HTS. The indels and adenines modified near or within the protospacer were analyzed and plotted. n = 2 replicates were performed. **b**, Edited cells were confirmed by HTS, passaged, and seeded onto a 24-well plate. Cells were transduced with baculoviral vectors expressing a modified GFP fused to a C-terminal peroxisomal targeting signal 1 (PTS1). Live-cell imaging was performed using a MICA confocal microscope. Imaging was conducted under blinded conditions for sgNT and *PEX1*-p.G843D-targeting guide identities. Two biological replicates were performed and > 30 cells were imaged and analyzed. Scale bar, 20 μm.

We assessed whether correction of *PEX1*-p.G843D variant restores peroxisome homeostasis in patient-derived fibroblasts. We nucleofected two patient-derived fibroblasts: homozygous *PEX1*^G843D/G843D^ and compound heterozygous *PEX1*^G843D/I700fs^ with ABE8e-V106W mRNA and a guide RNA targeting the *PEX1*-p.G843D (c.2528G>A) allele or a non-targeting guide RNA (sgNT) that does not match any human genome sequence^64^. We observed robust disease mutation correction efficiency exceeding 80% following treatment with ABE and the *PEX1*-p.G843D-targeting guide RNA (Extended Data Fig. 10a).

**Fig. 6 |:**
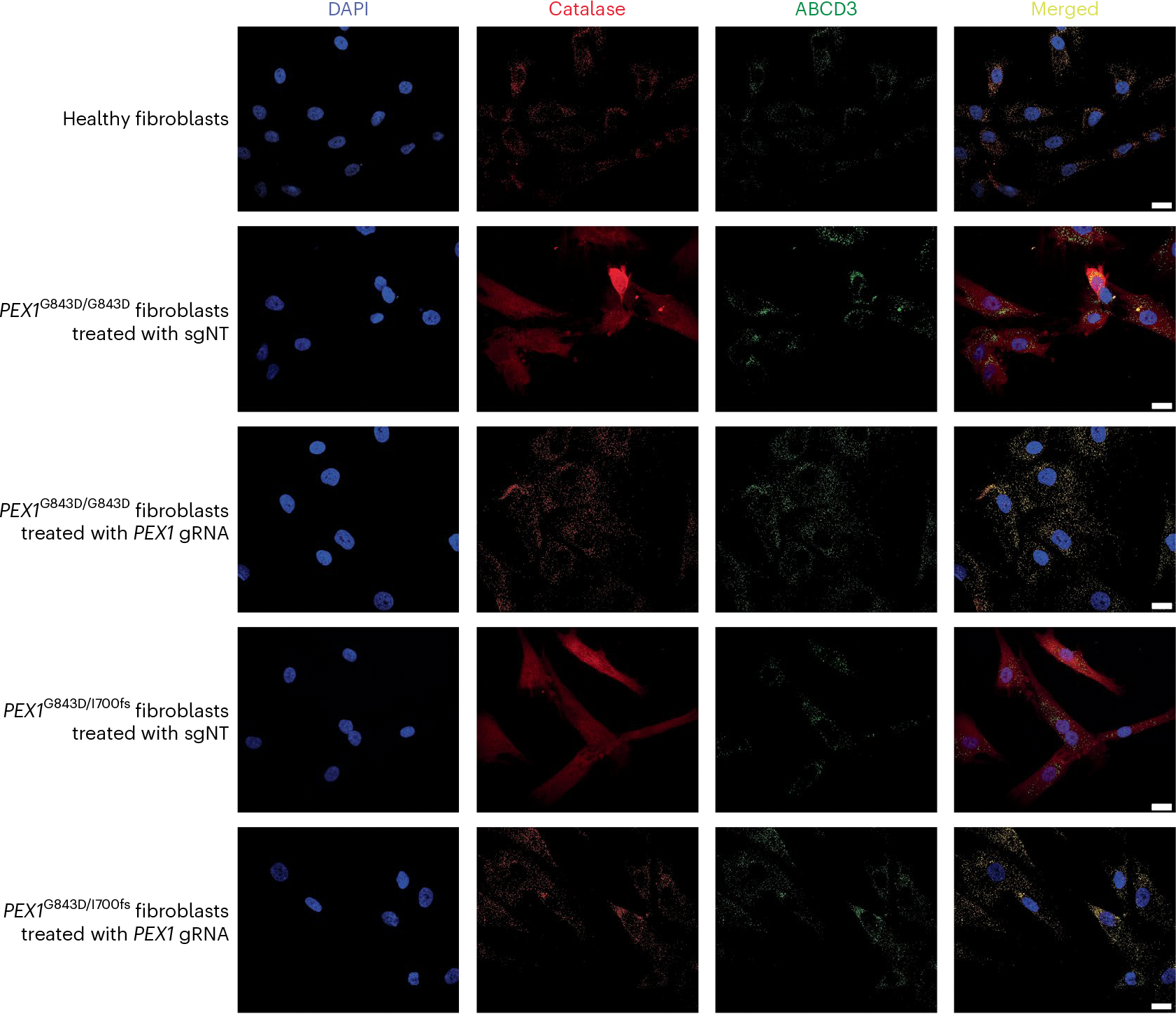
Correction of the *PEX1*-p.G843D allele in patient-derived fibroblasts restores peroxisomal homeostasis. Immunofluorescence analysis of peroxisomes in patient-derived fibroblasts treated with ABE8e-V106W. Catalase and ABCD3 were detected using primary antibodies and fluorophore-conjugated secondary antibodies to assess the subcellular localization of peroxisomal proteins in fibroblasts treated with a *PEX1*-p.G843D-targeting gRNA or non-targeting gRNA (sgNT), along with editor mRNA. Catalase is shown in red, ABCD3 in green, and nuclei stained with DAPI (blue). Co-localization of ABCD3 (green foci) and catalase (red foci) appears as yellow foci, consistent with normal peroxisomal assembly. More than 50 cells were imaged and analysed from randomly sampled slides areas from 3 biological replicates. Representative images are shown; scale bars, 20 μm.

The *PEX1*-p.G843D allele, like other pathogenic *PEX1* variants, impairs peroxisome biogenesis and results in mislocalization of peroxisome matrix proteins such as catalase^5^. To evaluate peroxisomal integrity, we stained catalase and the peroxisomal membrane protein ABCD3 using specific primary and fluorophore-conjugated secondary antibodies. Confocal microscopy revealed that sgNT-treated cells show diffuse cytosolic catalase signals alongside distinct ABCD3-positive foci, consistent with the known defects previously reported in ZSD patient-derived cells^5,65^ (Fig. 6, rows 2 and 4). By contrast, fibroblasts treated with the *PEX1*-p.G843D-targeting gRNA displayed catalase concentrated in punctate structures, co-localizing with ABCD3 foci, closely resembling healthy donor fibroblasts (Fig. 6, rows 1, 3 and 5). These findings indicate that correction of *PEX1*-p.G843D restores peroxisomal homeostasis.

To further assess peroxisomal assembly via live cell imaging, we transduced corrected and control *PEX1*^G843D/G843D^ fibroblasts with a baculovirus vector expressing a GFP reporter protein fused to an N-terminal peroxisome targeting signal 1 (GFP–PTS1)^66,67^. The sgNT-treated cells showed diffuse cytosolic distribution of GFP fluorescence or background GFP signals, indicating impaired import. By contrast, *PEX1*-p.G843D-corrected fibroblasts displayed robust punctate GFP–PTS1 structures similar to those observed in healthy control fibroblasts consistent with restored peroxisomal import and assembly (Extended Data Fig. 10b). Altogether, these results demonstrate that targeted correction of *PEX1*-p.G843D variant with ABE8e-V106W re-establishes peroxisomal matrix protein import and peroxisomal assembly in patient-derived cells.

## Discussion

Current treatments for ZSD are largely supportive and do not address the root cause that impairs the function of *PEX* genes required for normal peroxisome homeostasis^17,23^. Patients who are homozygous or compound heterozygous for the common *PEX1*-p.G843D allele frequently develop progressive liver disease associated with frequent hospitalizations and a shortened lifespan. Here we demonstrate that a *Pex1*-targeted base editing strategy to rescue liver functions in a dose- and time-dependent manner in a ZSD mouse model. Although further studies are needed to comprehensively assess safety and long-term efficacy, the translation of this approach to patients with liver disease has the potential to markedly improve quality of life and longevity^16^. Importantly, international newborn screening for ZSD^21,22^ provides an opportunity for deploying genetic therapies before irreversible liver and CNS damage occurs.

We identified ABE8e-V106W as a promising base editor to correct the common *PEX1*-p.G843D pathogenic variant in patient-derived fibroblasts and in homozygous *Pex1*-p.G844D ZSD mice^68,69^ (Fig. 1d and Supplementary Note 1). In patient-derived fibroblasts, mRNA delivery corrected >80% *PEX1*-p.G843D alleles, and in vivo AAV9 delivery produced up to 60% correction in bulk liver following a single treatment, enabling permanent and precise correction of the root cause of peroxisomal dysfunction. Since hepatocytes, the primary liver cell type transduced by AAV9, make up ~60% of the mouse liver^50^, these results suggest efficient base editing in the vast majority of hepatocytes^70^.

Dose–response studies in the neonatal ZSD mouse showed that even the lowest dose tested achieved near-maximal editing by 16 weeks after injection. Editing efficiency nearly doubled (1.8-fold increase) between weeks 6 and 16. Consistent with the increased efficiency in DNA correction over time, transcriptomic, lipidomic and histological analysis showed a robust rescue of hepatic gene expression and most fatty acid profiles normalizing to WT or near-WT levels by week 16. Despite these improvements, hepatic C26:0-LPC levels remained increased. This is because phospholipid and lysophospholipid levels reflect contributions from de novo synthesis in the endoplasmic reticulum and mitochondrial membranes^71^, remodelling via the Lands cycle^72^, degradation by lysophospholipases^72^ and biliary clearance^73,74^. Further investigation is needed to dissect how these processes contribute to a persistent C26:0-LPC increased. Moreover, the activities of extrahepatic tissues could influence circulating and hepatic C26:0-LPC levels. For example, hematopoietic stem cell transplantation reduces increased circulating C26:0-LPC levels in patients with cerebral adrenoleukodystrophy^75^, raising the possibility that hematopoietic-derived cells from healthy donors also could influence plasma C26:0-LPC levels. Overall, our results highlight heterogeneity in the metabolism of specific VLCFA-containing lipid species, such as those present in membranes, which should be considered when selecting biomarkers for future studies.

Regardless, this lipid heterogeneity did not prevent base editor-mediated rescue of animal growth, liver histology or liver transcriptome abnormalities in *Pex1*^G844D/G844D^ mice. Indeed, base editing in *Pex1*^G844D/G844D^ mice resulted in rescue of hepatic transcriptomic signatures relevant to lipid metabolism and peroxisome functions, as well as physiological responses to liver injury, fibrosis and fatty liver disease (Fig. 3b and Supplementary Table 5). The progressive increase in editing and near-complete phenotypic rescue between weeks 6 and 16 likely reflects sustained ABE expression from AAV9 (Extended Data Fig. 2c), hepatocyte proliferation and/or selective advantage among edited cells. Our findings collectively support the potential of ABE8e-V106W-mediated gene correction as a therapeutic strategy for ZSD-associated liver disease, even at comparatively low vector doses.

Despite the anticipated low to minimal gene editing in the CNS following systemic ABE-AAV9 delivery, we observed a significant reduction in brain BCFAs (phytanic and pristanic acids), but not VLCFAs (Extended Data Fig. 8). Unlike VLCFAs that are primarily made endogenously^76,77^, phytanic acid is exclusively diet-derived in mammals^78^. BCFA accumulation in the *Pex1*^G844D/G844D^ mouse cortex most likely results from circulating phytanic acid^77,78^ that crosses the blood–brain barrier, but is poorly metabolized by peroxisomal alpha-oxidation in the brain. Restoring hepatic alpha-oxidation reduces the circulating pool of phytanic acid available to enter the brain. This finding has translational implications, since it suggests that an effective gene editing correction of the peroxisomal alpha-oxidation pathway in liver could help protect the CNS and other tissues from toxic accumulation of diet-derived BCFAs. This observation also has implications for the treatment of adult Refsum disease, in which impaired *PHYH*-mediated alpha-oxidation can lead to toxic accumulation of phytanic acid, resulting in neurological and sensory deficits^79^.

Finally, ABE8e-V106W treatment of 4-week-old ZSD mice, representing a more advanced liver disease stage, also demonstrated near-complete rescue of liver genomic, lipidomic and histological abnormalities. Given the progressive course of liver disease in our preclinical mouse model and the patient population, this result suggests that the therapeutic window for treating ZSD liver disease by gene correction strategies may be long enough to benefit a substantial proportion of patients. Collectively, these results support the potential of using a base editing therapeutic strategy to address chronic liver disease found in many patients with ZSD. In our mouse model, additional ABE dose-escalation studies could further elucidate how varying levels of hepatic editing influence the rescue of liver functions. Such findings could guide the design of future human clinical trials. Non-viral LNP delivery of base editor mRNA demonstrated the potential to correct pathogenic *PEX1* alleles and restore peroxisomal function. We also expect that chemical modification of the mRNA^80^ and sgRNA^81^ will further improve RNA stability and editing efficiency. Although re-dosing remains a challenge for AAV delivery systems owing to neutralizing antibodies^82^, transient delivery systems such as LNPs^59,83^ or engineered virus-like particles^84,85^ in conjunction with adeno-associated vehicles that have brain and retina tropism could open new therapeutic opportunities for treating multisystemic genetic disorders such as ZSD in a variety of other tissues and organs, including the retina, brain and cochlea.

## Methods

### Clinical analysis of liver disease in individuals with ZSD

Patients with a diagnosis of a peroxisome biogenesis disorder were enrolled in a longitudinal, retrospective natural history study at the Research Institute of the McGill University Health Center with informed consent. Patients were recruited internationally, 87% from North America, 2.7% Australia, 1.3% Europe and 0.7% South America. Medical records were requested from birth to study entry, and annually thereafter from each participant’s healthcare institutions following authorization from the patient or parent/legal representative. De-identified longitudinal clinical data were collected from participants with at least one *PEX1*-c.2528G>A (p.G843D) allele. Patients without any liver-related abnormalities were classified as no liver disease (normal); patients reported with any of the following—hepatomegaly, jaundice, increased liver enzymes in blood or coagulopathy—were considered to have some hepatic dysfunction; patients diagnosed with cirrhosis, portal hypertension, esophageal varices, gastrointestinal bleeding, ascites or hepatic cancer were classified as severe liver disease. The study was registered on ClinicalTrials.gov (NCT01668186) where the study protocol and analysis plan are provided (also see ‘Reporting summary’). Sex, genotypes, age and the liver disease category are provided in Source Data Fig. 1b.

### Mammalian cell culture conditions

ZSD patient-derived *PEX1*^G843D/I700fs^ (catalogue ID GM16510) and healthy donor-derived primary skin fibroblasts (catalogue number GM03348) were purchased from the Coriell Institute for Medical Research. ZSD patient-derived homozygous *PEX1*^G843D/G843D^ primary skin fibroblasts were obtained from the Peroxisomal Disease Laboratory at the Kennedy Krieger Institute. Fibroblasts were cultured in Dulbecco’s modified Eagle medium (DMEM) supplemented with GlutaMAX (Thermo Fisher Scientific) and 15% (v/v) fetal bovine serum (FBS) (Thermo Fisher Scientific). HEK293T cells were purchased from the American Type Culture Collection (ATCC). All cells were cultured at 37 °C with 5% CO_2_. Cell lines were authenticated by their respective suppliers and tested negative for mycoplasma.

### Molecular cloning

Plasmids were constructed using Gibson assembly or restriction enzyme cloning. PCR amplification was performed with PhusionU Green Multiplex PCR Master Mix (Thermo Fisher Scientific). Gene fragments and plasmid backbones were generated by PCR or restriction digestion, or purchased from IDT. Recombinant plasmids were assembled using NEBuilder HiFi DNA Assembly Master Mix or T4 DNA ligase (NEB). Mach1 (Thermo Fisher Scientific) and NEB Stable (High Efficiency) chemically competent *Escherichia coli* cells were used for plasmid propagation.

### Electroporation of patient-derived fibroblasts

Patient-derived fibroblasts at 80–90% confluence in T-75 flask were washed with phosphate-buffered saline (PBS) (Thermo Fisher Scientific), trypsinized using TrypLE Express (Thermo Fisher Scientific) and resuspended in 10 ml of media. Cells were centrifuged at 110*g* for 5 min. During centrifugation, RNA reagents were prepared: for each sample, 1 μg base editor mRNA and 50 pmol of synthetic gRNA were mixed (2 μl total) and combined with 20 μl of P2 Primary Cell Nucleofector Solution and Supplement Solution mixture (Lonza, P2 Primary Cell 96-well Nucleofector Kit) per the manufacturer’s protocol. Pelleted cells were washed with PBS and resuspended in Lonza buffer. Twenty microlitres of cell suspension (~200,000 cells) was added to each editor/gRNA mixture, transferred to Nucleocuvette plates (Lonza) and electroporated using programme DS150 on a Lonza 4D nucleofector with X unit. After electroporation, 80 μl of media was added and incubated for 10 min at room temperature. Cells were then seeded into pre-equilibrated 24-well plates containing 1 ml medium per well and cultured for 3 days before lysis and sequencing.

### HTS of genomic DNA

Seventy-two hours after nucleofection, cells were washed with PBS and lysed for 1 h at 37 °C in lysis buffer (10 mM Tris-HCl pH 8, 0.05% SDS and 25 μg ml^−1^ proteinase K (Thermo Fisher Scientific)). Lysate was then heat-inactivated at 80 °C for 30 min. Lysate (1 μl) was used as an input for PCR1. PCR1 reactions (25 μl total volume) used Phusion Hot Start II kit (Thermo Fisher Scientific) or PhusionU Green Multiplex PCR Master Mix, and 0.125 μl of each 100 μM primer (sequences listed in Supplementary Table 7). PCR1 was performed under the following cycle conditions: 98 °C for 2 min, (98 °C for 10 s, 61 °C for 20 s, 72 °C for 30 s) × 28 cycles, 72 °C for 2 min.

Samples were barcoded by a second PCR reaction (PCR2). PCR2 reactions were 25 μl total, using the PhusionU Green Multiplex PCR Master Mix, 1.25 μl each of 10 μM Illumina barcoding primers, and 1 μl of PCR1. All PCR2 reactions were performed using the following cycling conditions: 98 °C for 2 min, (98 °C for 10 s, 61 °C for 20 s, 72 °C for 30 s) × 10 cycles, 72 °C for 2 min. After PCR2, samples of similar lengths were pooled and gel-extracted from a 1.5% agarose gel using a QIAquick gel extraction kit (Qiagen). Concentrations of purified libraries were determined using a Qubit Double-Stranded DNA High Sensitivity kit (Thermo Fisher Scientific) according to the manufacturer’s instructions. Libraries were diluted to 4 nM and sequenced on a MiSeq (Illumina) using single-read cycles.

### HTS data analysis

Samples were demultiplexed with MiSeq Reporter (Illumina). CRISPResso2 was used to analyse demultiplexed reads. Samples were aligned to the WT amplicon in batch mode, using the following parameters: ‘-q 30’ and ‘-qwc’. The value of the qwc parameter defined the portion of the sequence to be analysed for indels. The qwc interval covered the entire protospacer and adjacent nucleotides (26–30 bp quantification window). Percent indels were calculated as the sum of the reads containing insertions and deletions divided by ‘Reads_aligned’ in the CRISPResso_quantification_of_editing_frequency.txt output file. Percent editing was calculated from the specified base conversion in the genomic sequence using the Nucleotide_percentage_summary.txt output file.

### In vitro transcription of editor mRNA

In vitro transcription (IVT) of editor mRNA was performed as previously described^86^ with minor modifications. Editor sequences were cloned into pT7 expression plasmids (example Addgene number 178113). Linear DNA templates were generated by PCR using the Phusion U Green Multiplex Master Mix (NEB) and purified using the QIAquick PCR Purification kit (Qiagen). IVT reactions used a T7 High Yield RNA Synthesis kit (NEB), following the manufacturer’s directions with two exceptions: CleanCap Reagent AG (TriLink BioTechnologies) was added and the uridine-5′-triphosphate in the kit was replaced with *N*^1^-methylpseudouridine 5′ triphosphate (TriLink BioTechnologies). Reactions were incubated at 37 °C for 2 h and RNA was purified using lithium chloride (Thermo Fisher Scientific) precipitation, followed by 70% ethanol wash, and resuspended in nuclease-free water. RNA quality was verified by 2% agarose gel electrophoresis, diluted to 2 μg μl^−1^ and stored at −80 °C.

### Lentivirus production and transduction of patient-derived fibroblasts

Lentiviral particles encoding ABE8e-V106W and gRNA were generated in HEK293T using standard three-plasmid packaging (lentiviral transfer plasmid; psPAX2, Addgene number 12260; and pMD2.G, Addgene number 12259). Cells were transfected with Lipofectamine 2000 (Thermo Fisher Scientific) following the manufacturer’s recommended protocol, and 48 h later, viral supernatant was centrifuged at 3,000*g* for 15 min, filtered (0.45 μm) and stored at −80 °C. Lentiviral transduction of ZSD patient-derived fibroblasts was performed as reported previously^87^ with minor modifications. Fibroblasts were plated in 6-well culture plates at a density of 1 × 10^5^ cells per well and incubated for 18 h. The lentiviral supernatant was added at high multiplicity of infection (MOI) in the presence of 6 μg ml^−1^ polybrene and switched to puromycin-containing medium (2 μg ml^−1^) after 8 h for selection. Cells were expanded under puromycin-selection media for 2 weeks and then lysed for gene-editing and off-target analysis.

### AAV production

AAV9 vectors were produced by triple transfection method^48^ with minor modifications or obtained from the University of Massachusetts Chan Medical School Viral Vector Core. In brief, HEK293T/17 cells were transfected with PEI containing 5.7 μg of AAV genome, 11.4 μg of pHelper (Clontech) and 22.8 μg of AAV9 rep-cap plasmid per plate. The following day, media was exchanged for DMEM (5% FBS). Three days after, cells were collected and resuspended in 500 μl hypertonic lysis buffer (40 mM Tris base, 2 mM MgCl_2_, 500 mM NaCl and 100 U ml^−1^ salt-active nuclease, ArcticZymes Technologies) per plate and incubated at 37 °C for 1 h. The media was decanted and combined with 5× solution of polyethylene glycol (PEG) 8000 (Sigma-Aldrich) and NaCl to achieve a final concentration of 8% PEG and 500 mM NaCl. This solution was incubated on ice for 2 h or overnight to facilitate PEG precipitation and then centrifuged (3,200*g*, 30 min). The pellet was resuspended in 500 μl hypertonic lysis buffer per plate. This was added to the cell lysate, which was either immediately ultracentrifuged or stored at 4 °C overnight. Cell lysates were first clarified by centrifugation at 3,400*g* for 10 min and viral particles were purified by ultracentrifugation at 465,800*g* for 2 h 15 min at 4 °C with an iodixanol step gradient, followed by buffer exchange and concentration using 100 kDa MWCO columns (EMD Millipore). The concentrated viral solution was sterile-filtered using a 0.22 μm filter and stored at 4 °C until use. All viruses were titered via quantitative PCR using the AAVpro Titration Kit v.2 following the manufacturer’s protocol (Takara Bio).

### LNP formulation and characterization

LNPs were prepared in a microfluidic chip device by mixing an aqueous phase containing the mRNA with an ethanol phase containing the lipids^88^. The aqueous phase was prepared in a 10 mM citrate buffer with either ABE8e mRNA or sgRNA targeting *PEX1* or a non-targeting sgRNA control (5′-GAUCUCGCUUAUAUAACGAG-3′). The ethanol phase was prepared by solubilizing a mixture of OF-02 (Cayman Chemical), 1,2-dioleoyl-sn-glycero-3-phosphoethanolamine (DOPE; Avanti Polar Lipids), cholesterol (Sigma-Aldrich) and 1,2-dimyristoyl-sn-glycero-3-p hosphoethanolamine-*N*-[methoxy(polyethylene glycol)-2000] (C_14_-PEG_2000_; Avanti Polar Lipids) at a molar ratio of 35:16:46.5:2.5 (OF-02/DOPE/cholesterol/C_14_-PEG_2000_) with an ionizable lipid/mRNA weight ratio of 10:1. The aqueous and ethanol phases were mixed in a microfluidic device at a 3:1 ratio by syringe pumps to a final mRNA concentration of 0.15 mg ml^−1^. The resultant formulation was dialysed overnight against PBS in a 20 kDa molecular weight cut-off dialysis cassette (Thermo Fisher Scientific) at 4 °C. Following dialysis, LNPs were concentrated using Amicon 100 kDa molecular weight cut-off centrifugation filters (Sigma-Aldrich) at 4 °C. Total RNA concentration in the LNP solution was measured with a Stunner spectrophotometer (Unchained Labs). LNPs were stored in a PBS solution with 10% (w/v) sucrose at −80 °C until further use.

### ZSD mouse models

All animals were *Mus musculus*. Mixed genetic background mice (B6129F1-*Pex1*^<tm1.1Sjms>/Lutzy^/Mmjax) were generated by crossing heterozygous B6.Cg-*Pex1*^tm1.1Sjms/^Mmjax (JAX, stock number 25065) with heterozygous 129S6.Cg-*Pex1*^tm1.1Sjms^/Mmjax (JAX, stock number 30931). All mice (3–5 per cage) were housed in individually high-efficiency particulate air-filtered polysulfonate cages, under controlled conditions of 12 h/12 h light/dark cycles (6 am to 6 pm), a room temperature of 22 ± 4 °C and 50 ± 15% humidity, with 15 air exchanges per hour. Animals had ad libitum access to acidified water (pH 2.5–3.0) and standard chow. Animals were monitored daily for welfare and survival. All experiments followed National Institutes of Health (NIH) guidelines and The Jackson Laboratory IACUC (protocol 20029–1).

### Animal treatments

For neonatal treatments, P_1_ mice were cryo-anaesthetized and injected intravenously via the facial vein with 5 μl of gene editors or vehicle (PBS). Pups were returned to their dam after recovery. For young adult treatments, P28 mice were anaesthetized with isoflurane and injected retro-orbitally with up to 200 μl of gene editors or vehicle. Mice were returned to their home cage after recovery and monitored daily for survival and adverse effects; those with >20% loss of maximum body weight or body conditioning score <2 were humanely euthanized. For LNP intravenous injections, a 1:1 RNA weight ratio of mRNA-LNP/sgRNA-LNP was administered via retro-orbital injection. Experimental groups were sex-balanced and randomly assigned at dosing, with exact *n* sizes reported in figure legends. Investigators were blinded to treatment groups during outcome assessment.

### Mouse tissue sample preparation

All mice were euthanized at 6 or 16 weeks of age by CO_2_ asphyxiation and perfused with PBS. The liver was divided into three portions. The upper fifth was fixed in 10% neutral buffered formalin (NBF) for 24 h, rinsed in PBS, paraffin embedded and sectioned at 5 μm for histology. The largest lobe was fixed in 4% paraformaldehyde for 24 h, rinsed in PBS for 15 min and placed in 30% sucrose for 24 h before embedding in optimal cutting temperature solution and cryosectioning at 10 μm for histological staining. Remaining tissue was snap-frozen in liquid nitrogen and stored at −80 °C until use.

### Blood collection

Mice were anaesthetized with 2% isoflurane in oxygen at 6 or 16 weeks of age. Blood was collected retro-orbitally into a K_2_EDTA tube (Thermo Fisher Scientific). Samples were centrifuged at 4 °C at 15,000*g* for 5 min to separate plasma from blood cells. Plasma and the cell fractions were stored at −80 °C until use.

### Histology analysis

ORO staining was used to detect lipids in liver cryosections. Sections were stained with ORO stock solution saturated with isopropanol (Rowley Biochemical Institute), followed by 10% NBF, Mayer’s haematoxylin (Sigma-Aldrich) and lithium carbonate before coverslipping (Corning). PAS was used to detect glycogen in paraffin-embedded liver sections, treated with 0.5% periodic acid for 5 min, Schiff’s reagent (Poly Scientific) for 30 min, and counterstained with Mayer’s haematoxylin (Fisher Scientific). To minimize batch variation, slides for each time point were processed together. Slides were scanned using a Nanozoomer S210 (Hamamatsu) at 40× magnification. Three regions of interest per section were selected at 10× magnification for analysis. The average stained area relative to the total tissue was quantified using CellProfiler (version 4.2.4; pipeline provided in Supplementary Information).

### Mouse tissue gDNA purification

Brain, eye and liver frozen tissues were processed using the DNAdvance kit (Beckman Coulter) according to the manufacturer’s instructions. Approximately 50 mg ground frozen tissue was placed in 1.5 ml tubes and lysed in 500–1,000 μl lysis buffer supplemented with proteinase K overnight at 55 °C in a benchtop shaker (800 rpm). Lysates were bead-purified and washed with 70% ethanol. Purified genomic DNA was quantified by spectrophotometry and used for HTS or droplet digital PCR.

### RNA-seq and data analysis

Total RNA was extracted from liver using the RNeasy kit (QIAGEN) and quantified using Qubit HS (Thermo Fisher Scientific). Libraries were prepared from 20 ng RNA using a template-switching protocol with Maxima RT (Thermo Fisher Scientific) and KAPA HiFi HotStart ReadyMix (Roche), followed by SPRI bead cleanup (Beckman Coulter). Libraries were indexed with Nextera adapters (Illumina) and sequenced on an Illumina NovaSeq 6000 using S2 chemistry. Samples with <25 million reads were excluded.

FASTQ files were generated with bcl2fastq v2.20, trimmed with Trim Galore v0.6.7, aligned to GENCODE mouse reference genome M31 (GRCm39) using STAR (v2.7.10a), quantified with kallisto and refined to canonical coding sequences using CCDS release 21.

Differential expression was analysed with PyDESeq2 using the model:

$$\text{log}\;q\;\sim \;\beta_{0}+\beta_{1}\times (\text{treatment})+\beta_{2}\times (\text{sex}).$$

Vehicle-treated WT or *Pex1*^G844D/G844D^ mice served as the reference level for the treatment variable (depending on the analysis) and male mice as the reference level for the sex variable. Inferred log_2_ fold-change (FC) values were shrunk with a heavy-tailed Cauchy prior, and *P* values were calculated according to Wald’s test. Reported effect sizes correspond to post-shrinkage log_2_FC values.

Volcano plots are shown in Extended Data Fig. 4, and DEGs were further analysed via WebGestalt^89^ (https://www.webgestalt.org/). KEGG, Phenotype and other related pathway analyses were conducted and shown in Extended Data Fig. 5 and Supplementary Table 5, focusing on the *Pex1*^G844D/G844D^ vehicle versus WT vehicle at 6 weeks and 16 weeks after injection. Enrichment analyses focused on categories meeting a 1% FDR and defined by 10–1,000 genes, thereby avoiding overly specialized or broad categories.

Analysis of the transcriptome-wide editing RNA-seq data was performed as follows. FASTQ files were generated using Bcl2fastq2, then trimmed using Cutadapt^90^ v.1.18 to remove adapter sequences, unpaired sequences and low-quality bases. We created sorted bam alignments using STAR v.2.7.11a to align paired reads from each of three biological replicates to the GRCm38/mm10 mouse reference genome (Ensembl). To calculate the average percentage of A-to-I editing among adenosines sequenced in transcriptome-wide sequencing analysis, we used REDItools2. We removed all nucleotides except adenosines from our analysis and then removed all adenosines with a read coverage less than 10 to avoid errors owing to low sampling; in addition, we removed positions with a mapping or read quality score below 25. Next, we calculated the number of adenosines that were converted to an inosine in each sample and divided this by the total number of adenosines in our dataset after filtering to obtain a percentage of adenosines that were edited to inosine in the transcriptome.

To investigate how transcriptome-wide A-to-I editing levels vary with AAV dose and time, we focused our investigation on a set of high-confidence A-to-I off-targets. This set of sites was determined by identifying adenosines with non-zero (≥0.1%) A-to-I editing in either both male high-dose mice at 6 weeks or both female high-dose mice at 6 weeks (22,131 As), then removing all overlap with sites that also exhibit non-zero (≥0.1%) A-to-I editing in at least 2 saline-treated control samples (96,097 As). This filtering procedure left 4,161 adenosines that we investigated as candidate off-target A-to-I editing sites. Jitter plots were generated for each sample by plotting each of the 4,161 off-target sites that showed non-zero editing.

### Lipid analysis

Plasma and tissue fatty acids were hydrolysed from triglycerides and phospholipids by acidification with 90:10 (v/v) acetonitrile/6 N HCl, followed by neutralization with 90:10 (v/v) methanol/10 N NaOH. Samples were incubated at 104 °C for 45 min and then reacidified in 6 N HCl. Total fatty acids were extracted in hexane and derivatized^91,92^ to pentafluorobenzyl esters. Samples were analysed by GC/MS in negative ion chemical ionization mode using ammonia as reagent gas (source pressure, 1.6 Torr) on a SP2560 capillary column (50 m × 0.25 mm × 0.2 μm) with helium carrier gas (column pressure, 20 psi)^93^. After a 1 μl 1:35 split injection, oven temperature was programmed: 60 °C for 2 min; ramped at 10 °C min^−1^ to 180 °C; then at 2.5 °C min^−1^ to 250 °C and held for 14 min (total run time, 55 min). The MSD transfer line was held at 240 °C for 33 min, then increased at 2.5 °C min^−1^ to 250 °C and held for 32 min. Data were acquired in select ion monitoring mode with dwell times of 50–100 ms per ion. Each analyte was matched to 1 of 13 stable-isotope internal standards. Quantification was based on calibration curves from 50 analyte standards; when pure standards were unavailable, the closest analogue was used. Individual fatty acids were expressed as μg ml^−1^ in plasma and as μg mg^−1^ of total lipid content. The lipid FC relative to WT controls for each lipid moiety was calculated using RStudio (version 2024.12.1_563; Supplementary Information) and *P* values calculated using Dunn’s nonparametric many-to-one comparison test for Kruskal-type ranked data.

### Bile acid analysis

Methanol, acetonitrile, water and formic acid (all Optima grade) were purchased from Fisher Scientific. d_4_-t-CA, d_4_-g-CA, d_4_-g-CDCA, d_4_-CA, d_4_-CDCA, t-CA, g-CA and CA were purchased from Cayman Chemical; 7-HOCA from Avanti Polar Lipids; and d_3_-THCA and d_3_-DHCA, THCA and DHCA from VUMC (Herman ten Brink). Liver tissue (25 mg) homogenized in 50 μl PBS was extracted with 0.5 ml methanol and sonicated for 30 min at room temperature (Branson 3510). Extracts were centrifuged at 600*g* for 10 min, and supernatants were dried under nitrogen and reconstituted in 240 μl of methanol/H_2_O (3:1 v/v) containing 100 ng internal standards. Samples were filtered through 0.22 μm nylon Spin-X filters (Costar) and transferred to injection vials (Phenomenex). Chromatographic separation used a Waters TQD with an Acquity UPLC system using an HSST3 column (1.8 μm, 50 × 2.1 mm) at 50 °C in negative-ion electrospray ionization mode. Flow rate was 0.5 ml min^−1^ with a gradient from 75% A (water/0.1% formic acid) and 25% B (acetonitrile/0.1% formic acid) to 10% A/90% B over 1 min, held for 1–2.25 min and then equilibrated to initial conditions by 3 min. Quantification used Masslynx 4.1 with TargetLynx integration; peak areas were exported to Excel; concentrations were expressed as pmol mg^−1^ protein and analysed in GraphPad Prism 10.

### Western blotting

Liver lysates were prepared in radioimmunoprecipitation assay buffer containing Halt Protease Inhibitor Cocktail (Thermo Fisher Scientific). Protein concentration was determined by detergent-compatible assay (Bio-Rad). Lysates were diluted to 0.75 mg ml^−1^ with 5× fluorescent master mix and heated at 95 °C for 5 min. ABCD3 protein levels were quantified using automated capillary western blotting (Simple Wes, ProteinSimple), with an anti-PMP70/ABCD3 antibody (Abcam, ab85550) at a 1:50 dilution. Chemiluminescent signals were analysed using Compass for Simple Western (version 6.2.0). ABCD3 peak area was normalized to total protein area^94,95^. FC values were calculated relative to WT controls.

### Droplet digital PCR

Genomic DNA from liver tissue was analysed by droplet digital PCR (ddPCR) using the Bio-Rad QX ONE platform. Reactions were prepared with 100 ng gDNA, target-specific primers and probes (Supplementary Table 2), 2× ddPCR Supermix (Bio-Rad), and processed according to the manufacturer’s guidelines. PCR cycling conditions were 95 °C for 10 min, 50 cycles of 94 °C for 30 s and 58 °C for 2 min, followed by 98 °C for 10 min. Data were analysed using QX ONE software (v1.3).

### Off-target editing analysis

CIRCLE-seq off-target analysis was performed as described previously^62,96^. In brief, gDNA from N2A mouse cells or ZSD patient-derived *PEX1*^G843D/I700fs^ fibroblasts was isolated using the Gentra Puregene Kit (Qiagen) according to the manufacturer’s instructions. Purified gDNA was sheared with a Covaris S2 instrument to an average length of 300 bp. The fragmented DNA was end repaired, poly-A tailed and ligated to a uracil-containing stem-loop adaptor using the KAPA HTP Library Preparation Kit, PCR Free (KAPA Biosystems). Adaptor-ligated DNA was treated with Lambda Exonuclease (NEB) and *E. coli* Exonuclease I (NEB), then with USER enzyme (NEB) and T4 polynucleotide kinase (NEB). Intramolecular circularization of the DNA was performed with T4 DNA ligase (NEB), and residual linear DNA was degraded by Plasmid-Safe ATP-dependent DNase (Lucigen). In vitro cleavage reactions were performed with 250 ng of Plasmid-Safe ATP-dependent DNase-treated circularized DNA, 90 nM of SpCas9 nuclease protein, Cas9 nuclease buffer (NEB) and 90 nM of synthetic chemically modified gRNA (Synthego) in 100 μl. Cleaved products were poly-A tailed, ligated with a hairpin adaptor (NEB), treated with USER enzyme (NEB) and amplified by PCR with barcoded universal primers (NEBNext Multiplex Oligos for Illumina, NEB) using Kapa HiFi Polymerase (KAPA Biosystems). Libraries were sequenced with 150-bp paired-end reads on an Illumina MiSeq instrument. CIRCLE-seq data were analysed using open-source CIRCLE-seq analysis software and default recommended parameters (https://github.com/tsailabSJ/circleseq).

Cas-OFFinder analysis was performed online (http://www.rgenome.net/cas-offinder/). Potential off-targets were filtered to ≤3 mismatches for SpCas9. Twenty predicted off-target sites were nominated.

The top 30 CIRCLE-seq-nominated off-target sites for the mouse genome and Cas-OFFinder nominated sites were examined via amplicon sequencing using the gDNA from editor-treated liver tissues or patient-derived fibroblasts, as specified in the texts and figures.

### Baculoviral peroxisome-GFP transduction of patient-derived fibroblasts

CellLight peroxisome-GFP, BacMam 2.0 kit was used (ThermoFisher Scientific, C10604). Healthy donor-derived fibroblasts, base editor-treated and non-target guide-treated control fibroblasts were plated onto 24-well plates (Greiner). On the second day, cells were transduced with baculoviral peroxisome-GFP vectors following the manufacture’s recommended protocol, incubated for 16 h and treated with far-red nuclear staining dye (MilliporeSigma) before imaging. Live-cell imaging used the MICA confocal microscope. Incubation temperature was set at 37 °C, and CO_2_ was set at 5%. The 24-well plate and imaging parameters (‘dynamics and living’) were set for live cell imaging. The absorbance and emission were set for the fluorophores specified in the product manual. A 63× water-immersion objective lens was used to capture images. Treated sample images for comparison were acquired using the same imaging settings. Images were processed further with ImageJ2 (version 2.14.0/1.54f).

### Immunofluorescence microscopy of ABE-treated patient-derived fibroblasts

Nucleofected patient-derived and healthy donor-derived fibroblasts were grown on sterile glass coverslips. Cells were washed with PBS and fixed using 3% formaldehyde in PBS for 20 min at room temperature, and permeabilized with 1% Triton X-100 in PBS (5 min). After washing, cells were incubated overnight at 4 °C with primary antibodies: anti-catalase (Thermo Fisher Scientific) and anti-PMP70 (ABCD3) (Abcam). The next day, cells were washed and incubated for 20 min at room temperature with Alexa Fluor 594 goat anti-rabbit IgG (Thermo Fisher Scientific) and Alexa Fluor 488 goat anti-mouse IgG (Thermo Fisher Scientific). Cells on coverslips were washed and mounted onto glass slides with a drop of Prolong Diamond Antifade Mountant (Thermo Fisher Scientific). Images were acquired using Leica MICA-recommended settings for fixed cells and processed with ImageJ2.

### Quantification and statistical analysis

The number of independent biological replicates and technical replicates for each experiment is provided in the figure legends or the ‘Methods’ section. Statistical tests were performed using GraphPad Prism version 10.1.1 for Windows and 10.3.0 for Mac (GraphPad Software).

## Extended Data

## Supplementary Material

Supp info

Reporting summary

Source data figs 1-6

Source data extended data

Source data fig 2d

Source data fig 4d

Supp tables

**Supplementary information** The online version contains supplementary material available at https://doi.org/10.1038/s41551-026-01651-5.

## Data Availability

The main data that support the results in this study are available within the paper and its Supplementary Information. High-throughput DNA sequencing FASTQ files have been deposited at the National Center of Biotechnology (NCBI) Information Sequence Read Archive under BioProject (PRJNA1162752). Plasmids are available through Addgene. Microscopy raw data are available via Figshare at https://doi.org/10.6084/m9.figshare.31129660 (ref. 97). Any additional information required to reanalyse the data reported in this paper is available from the lead contact upon request. Source data are provided with this paper.

## Code availability

All original code is available in Supplementary Notes 4–6. CellProfiler pipelines used to analyse the Oil Red O and periodic acid–Schiff-stained areas are available via Figshare at https://figshare.com/s/9eda20bcd55d6ce3511c (ref. 98).
