# Supplementary material for: In vivo base editing rescues liver pathophysiology and peroxisome dysfunction in a mouse model of Zellweger spectrum disorder
Source: Nat Biomed Eng. Author manuscript; Available in PMC 2026 Jun 12. (PMC13262281; doi:10.1038/s41551-026-01651-5)
Supplement: Supp info [file NIHMS2176319-supplement-Supp_info.pdf]

nature biomedical engineering

Supplementary information https://doi.org/10.1038/s41551-026-01651-5

In vivo base editing rescues liver pathophysiology and peroxisome dysfunction in a mouse model of Zellweger spectrum disorder

In the format provided by the authors and unedited

Supplementary Information

Supplementary Tables 1-7 | (Provided as a separate Excel spreadsheet)

Supplementary Note 1 | Assessment of compact ABEs in the PEX1$^{G843D/G843D}$ patient-derived fibroblasts

Supplementary Note 2 | Representative branched fatty acids, very long chain fatty acids, and plasmalogen in the lipid analysis

Supplementary Note 3 | Mature bile acids analysis of ABE-AAV9-treated 4-week-old mice

Supplementary Note 4 | Custom python script used for hierarchical cluster analysis of differentially expressed genes in RNA sequencing

Supplementary Note 5 | Custom python script used for lipids analysis

Supplementary Note 6 | Custom python script used for transcriptome off-target analysis

Supplementary Note 1 | Assessment of compact ABEs in the PEX1$^{G843D/G843D}$ patient-derived fibroblasts. In the Sup. Fig. 1 (shown below), editing efficiencies using compact ABEs were measured by High-throughput sequencing (HTS) and analyzed with CRISPResso2. The adenines within and near the protospacer are shown. Dots represent individual replicates (Sp-ABE8e-V106W n = 3; Sauri-ABE8e-V106W n = 4; SAKKH-ABE8e-V106W n = 4; eNme2-ABE8e-V106W n = 4; Cj-ABE8e-V106W n = 4; Non-targeting control n = 3). Bars indicate mean ± s.d.

Sup. Fig. 1

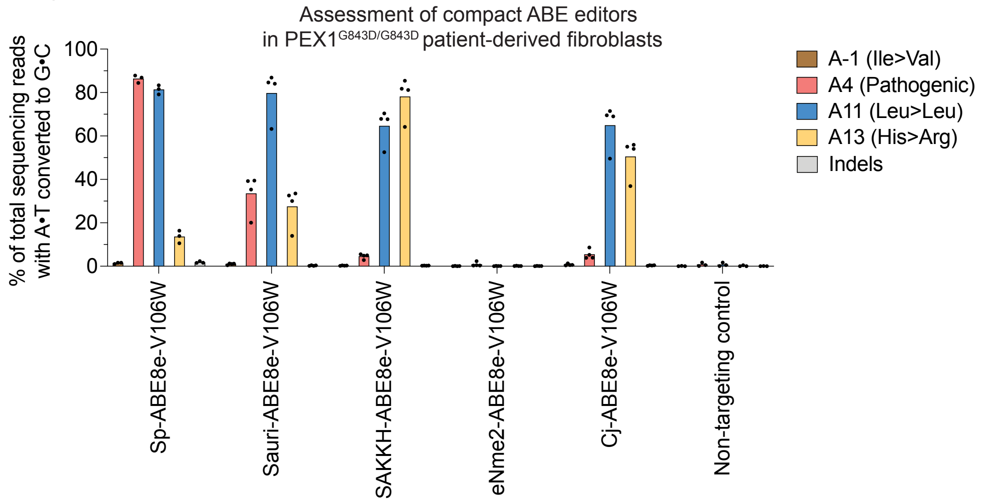

Supplementary Note 2 | Representative branched-chain fatty acids, very long-chain fatty acids, and plasmalogens in lipid analyses. In the Sup. Fig. 2 (shown below), we re-plotted data pertaining to the levels of the branched-chain fatty acid species, phytanic and pristanic acid, from volcano plots to individual bar graphs for improved clarity. Following ABE-AAV9 treatment, the previously elevated phytanic and pristanic acid in the *Pex1*$^{G844D/G844D}$ mice were fully restored to wild-type levels in both plasma and liver at 16 weeks post-injection. Experimental procedures are described in detail in the Methods section. Liver data were collected at 6 and 16 weeks. At 6 weeks, wild-type mice (n=6), *Pex1*$^{G844D/G844D}$ mice treated with vehicle (n=6), and *Pex1*$^{G844D/G844D}$ mice treated with ABE-AAV9 at doses of 1×10^{11} vg (n=5), 4×10^{10} vg (n=4), or 1×10^{10} vg (n=4) were analyzed. At 16 weeks, wild-type mice (n=6), *Pex1*$^{G844D/G844D}$ mice treated with vehicle (n=6), and *Pex1*$^{G844D/G844D}$ mice treated with AAV at doses of 1×10^{11} vg (n=7), 4×10^{10} vg (n=7), or 1×10^{10} vg (n=9) were analyzed using Kruskal-Wallis test corrected with the Dunn's multiple comparisons. Plasma data were also collected at 6 and 16 weeks. At 6 weeks, wild-type mice (n=5), Pex1$^{G844D/G844D}$ mice treated with vehicle (n=5), and *Pex1*$^{G844D/G844D}$ mice treated with AAV at doses of 1×10^{11}vg (n=6), 4×10^{10} vg (n=4), or 1×10^{10} vg (n=4) were analyzed. At 16 weeks, wild-type mice (n=5), *Pex1*$^{G844D/G844D}$ mice treated with vehicle (n=4), and *Pex1*$^{G844D/G844D}$ mice treated with AAV at doses of 1×10^{11} vg (n=7), 4×10^{10} vg (n=8), or 1×10^{10} vg (n=9) were analyzed using Kruskal-Wallis test corrected with the Dunn's multiple comparisons.

Sup. Fig. 2

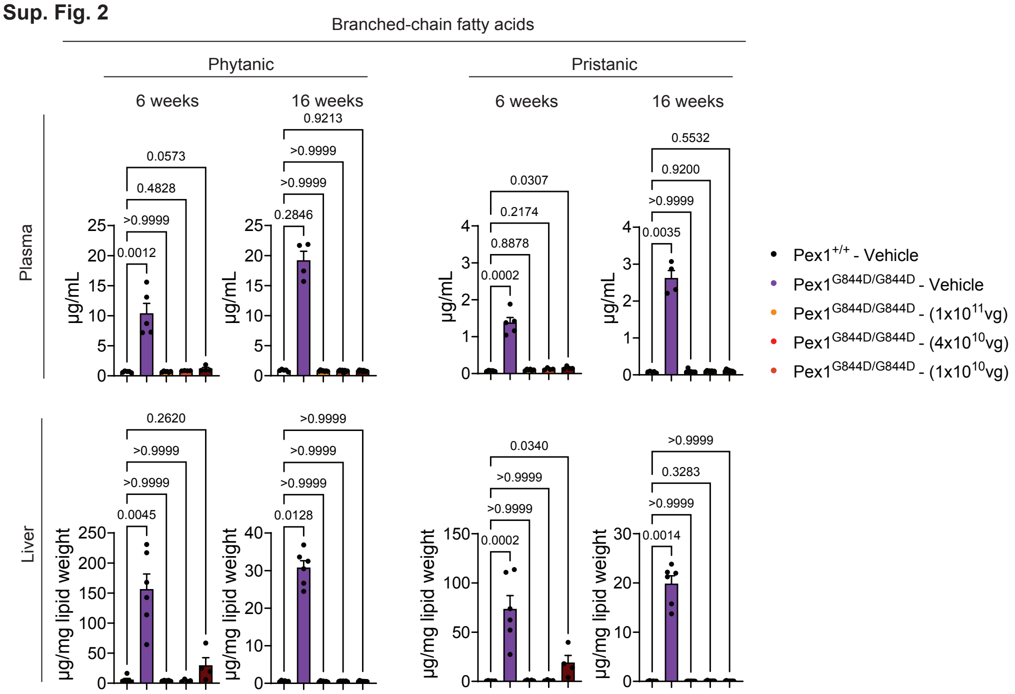

In the Sup. Fig. 3 and 4 (shown below), we plotted data pertaining to the levels of two very long-chain fatty acids (VLCFAs), C26:0 and C26:1, as well as C26:0-lysophosphatidylcholine (LPC) in bar graphs for improved clarity. Elevated C26:0 and C26:1 VCLFA levels in the *Pex1*$^{G844D/G844D}$ mice were restored to at or near wild-type level in the liver 16 weeks after ABE-AAV9 treatment. In contrast, elevated plasma C26:0 levels in the *Pex1*$^{G844D/G844D}$ mice were not substantially changed at 16 weeks after ABE-AAV9 treatment. However, elevated plasma C26:1 levels in the *Pex1*$^{G844D/G844D}$ mice were substantially reduced to near wild-type levels 16 weeks after ABE-AAV9 treatment.

For C26:0 and C26:1 analyses, liver and plasma samples were collected at 6 and 16 weeks. At 6 weeks, liver data included wild-type mice (n=6), *Pex1*$^{G844D/G844D}$ mice treated with vehicle (n=7 for C26:0; n=6 for C26:1), and *Pex1*$^{G844D/G844D}$ mice treated with AAV at doses of 1×10^{11} vg (n=6 for both), 4×10^{10} vg (n=4 for both), or 1×10^{10} vg (n=3 for C26:0; n=4 for C26:1). At 16 weeks, liver data included wild-type mice (n=6), *Pex1*$^{G844D/G844D}$ mice treated with vehicle (n=6 for both), and *Pex1*$^{G844D/G844D}$ mice treated with AAV at doses of 1×10^{11} vg (n=7 for both), 4×10^{10} vg (n=7 for both), or 1×10^{10} vg (n=9 for both). Plasma data at 6 weeks included wild-type mice (n=5), *Pex1*$^{G844D/G844D}$ mice treated with vehicle (n=5 for both), and *Pex1*$^{G844D/G844D}$ mice treated with AAV at doses of 1×10^{11} vg (n=6 for both), 4×10^{10} vg (n=4 for both), or 1×10^{10} vg (n=4 for both). At 16 weeks, plasma data included wild-type mice (n=5 for C26:0; n=6 for C26:1), *Pex1*$^{G844D/G844D}$ mice treated with vehicle (n=4 for C26:0; n=6 for C26:1), and *Pex1*$^{G844D/G844D}$ mice treated with AAV at doses of 1×10^{11} vg (n=7 for both), 4×10^{10} vg (n=8 for C26:0; n=7 for C26:1), or 1×10^{10} vg (n=9 for both). Data were analyzed using Kruskal-Wallis test corrected with the Dunn's multiple comparisons.

Sup. Fig. 3

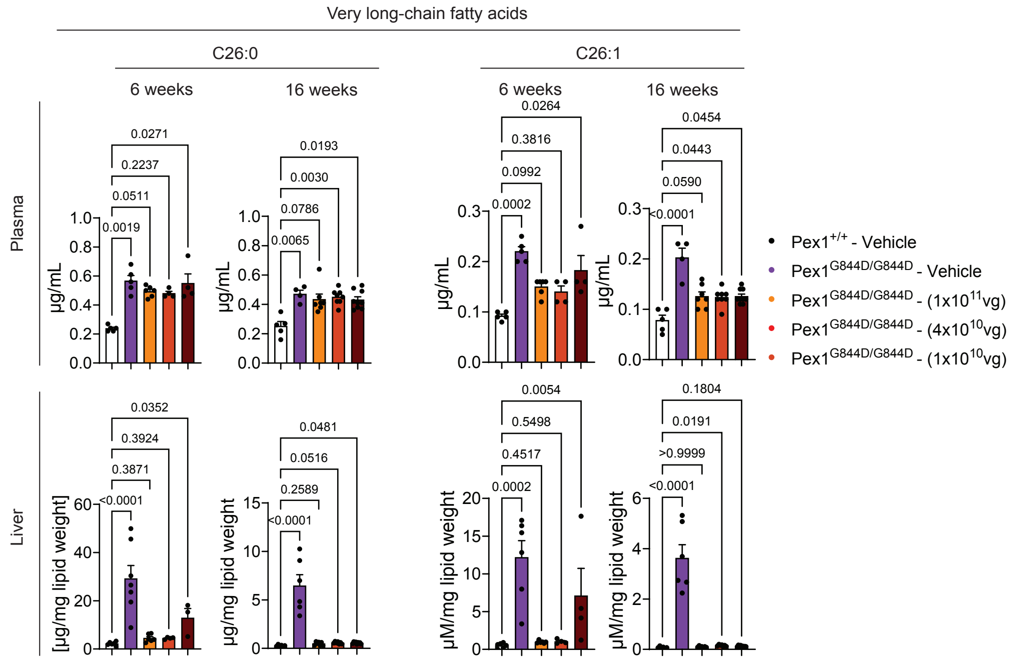

As shown in Sup. Fig. 4, ABE-AAV9 treatment did not rescue C26:0-LPC levels in plasma or liver which may reflect the turnover kinetics of VLCFAs in specific membrane lipids relative to those of total free and esterified VLCFAs present in all lipids, also including triglycerides[1], measured in **Fig. 2**. For C26 LPC analyses, liver and plasma samples were collected at 6 and 16 weeks. At 6 weeks, liver samples included wild-type mice (n=7), *Pex1*$^{G844D/G844D}$ mice treated with vehicle (n=5), and *Pex1*$^{G844D/G844D}$ mice treated with AAV at doses of 1×1^{11} vg (n=6), 4×10^{10} vg (n=4), or 1×10^{10} vg (n=5). Plasma samples at 6 weeks included wild-type mice (n=5), *Pex1*$^{G844D/G8448}$ mice treated with vehicle (n=5), and *Pex1*$^{G844D/G844D}$ mice treated with AAV at doses of 1×10^{11} vg (n=6), 4×10^{10} vg (n=4), or 1×10^{10} vg (n=6). At 16 weeks, liver samples included wild-type mice (n=6), *Pex1*$^{G844D/G844D}$ mice treated with vehicle (n=6), and *Pex1*$^{G844D/G844D}$ mice treated with AAV at doses of 1×10^{11} vg (n=7), 4×10^{10} vg (n=8), or 1×10^{10} vg (n=9). Plasma samples at 16 weeks included wild-type mice (n=6), *Pex1*$^{G844D/G844D}$ mice treated with vehicle (n=6), and *Pex1*$^{G844D/G844D}$ mice treated with AAV at doses of 1×10^{11} vg (n=7), 4×10^^{10}^ vg (n=9), or 1×10^{10} vg (n=9). Data were analyzed using Kruskal-Wallis test corrected with the Dunn's multiple comparisons.

Sup. Fig. 4

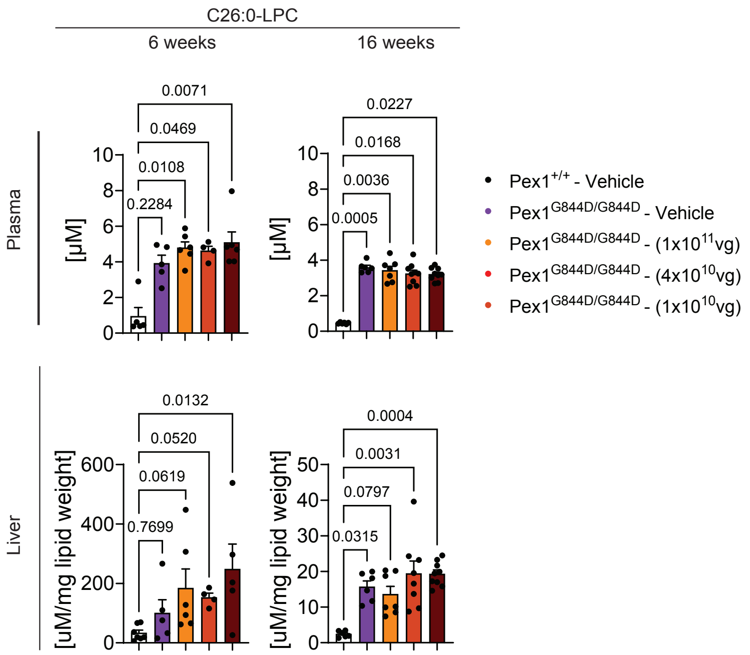

In addition to the VLCFA and BFCA data, we also examined the levels of plasmalogens, ether-phospholipids reliant on peroxisome biosynthesis, in red blood cells (RBCs). In the box plots shown in Sup. Fig. 5, we quantified the abundance of the C16:0 dimethylacetyl (DMA) and C18:0 DMA derivatives of the chemical moieties present in the *sn*-1 position of plasmalogens relative to C16:0 fatty acid methyl esters (FAME) and C18:0 (FAME). Results are reported as DMA/FAME ratios since the vinyl ether-linked groups and cognate fatty acids are converted to DMA and FAME derivatives, respectively, after RBC sample processing. In panel a, C16:0 DMA and C18:0 DMA levels did not differ among RBCs from wild type and *Pex1*$^{G844D/G844D}$ mice. In panel b, RBC C16:0 FAME levels were slightly elevated in RBCs from *Pex1*$^{G844D/G844D}$ mice relative to controls while C18:0 levels were unchanged. In panel c, we observed a significant reduction in 16:0 DMA/FAME ratios in RBCS from *Pex1*$^{G844D/G844D}$ mice. This effect was not rescued by ABE treatment, possibly because RBC membrane lipids are derived from the hematopoietic compartment which would not be expected to be substantively corrected by AAV9 transduction. C18:0 DMA/FAME ratios in RBCS from *Pex1*$^{G844D/G844D}$ mice did not differ from controls. For plasmalogen (C16:0 and C18) analyses, samples included wild-type mice (n=10), *Pex1*$^{G844D/G844D}$ mice treated with vehicle (n=10), and *Pex1*$^{G844D/G844D}$ mice treated with AAV at a dose of 1×10^{11} vg (n=9). Data were analyzed using two-way ANOVA corrected with the Tukey multiple comparisons.

Sup. Fig. 5

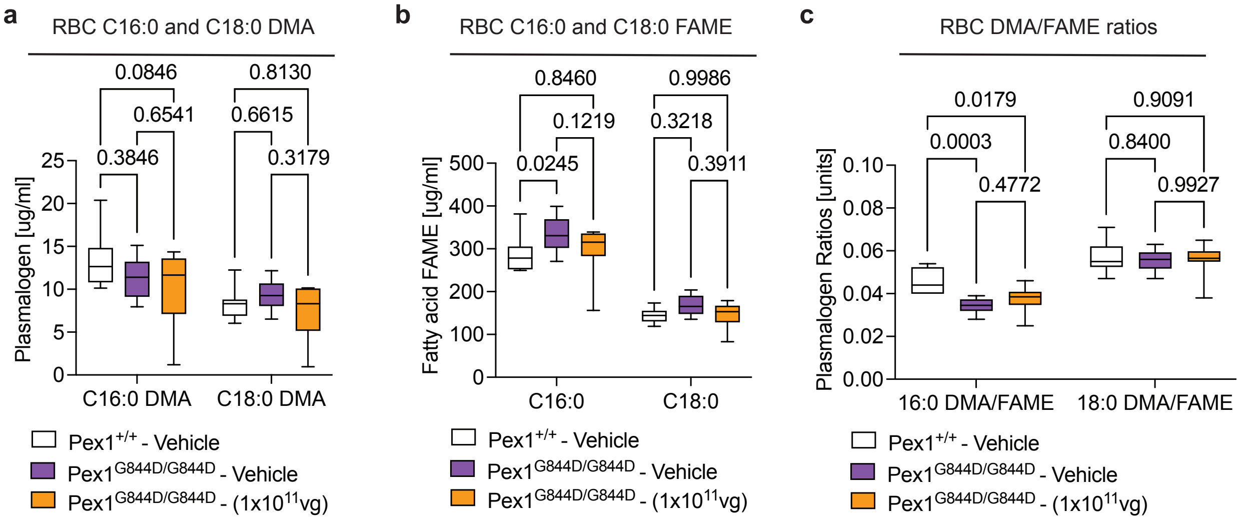
a
RBC C16:0 and C18:0 DMA
0.0846
0.8130
0.6541
0.6615
0.3846
0.3179
Plasmalogen [ug/ml]
25
20
15
10
5
0
C16:0 DMA
C18:0 DMA
b
RBC C16:0 and C18:0 FAME
0.8460
0.9986
0.1219
0.3218
0.0245
0.3911
Fatty acid FAME [ug/ml]
500
400
300
200
100
0
C16:0
C18:0
c
RBC DMA/FAME ratios
0.0179
0.9091
0.0003
0.8400
0.4772
0.9927
Plasmalogen Ratios [units]
0.10
0.08
0.06
0.04
0.02
0.00
16:0 DMA/FAME
18:0 DMA/FAME
Pex1+/+ - Vehicle
Pex1G844D/G844D - Vehicle
Pex1G844D/G844D - (1x1011vg)

Supplementary Note 3 | Mature bile acids analysis of ABE-AAV9-treated 4-week-old mice. Twelve weeks post-injection, liver bile acids were extracted from 4-week-old mice treated with ABE-AAV9 or vehicle control. Mature C24 bile acids were analyzed using chromatographic separation and mass spectrometry. Vehicle-treated WT *Pex1*$^{+/+}$ mice (n=5), vehicle-treated *Pex1*$^{G844D/G844D}$ mice (n=5), and AAV9-ABE8e-V106W-treated *Pex1*$^{G844D/G844D}$ mice (n=6) were analyzed (shown in the Sup. Fig. 6 below). Numbers above pairs of bars indicate p-values calculated using the statistical methods Brown-Forsythe and Welch ANOVA test corrected with the Dunnett comparison . Overall, mature bile acid levels in Pex1$^{G844D/G844D}$ mice treated with the ABE8e-V106W differed from vehicle-treated controls and approached levels observed in wild-type Pex1$^{+/+}$ mice. However, these differences did not reach statistical significance.

Sup. Fig. 6

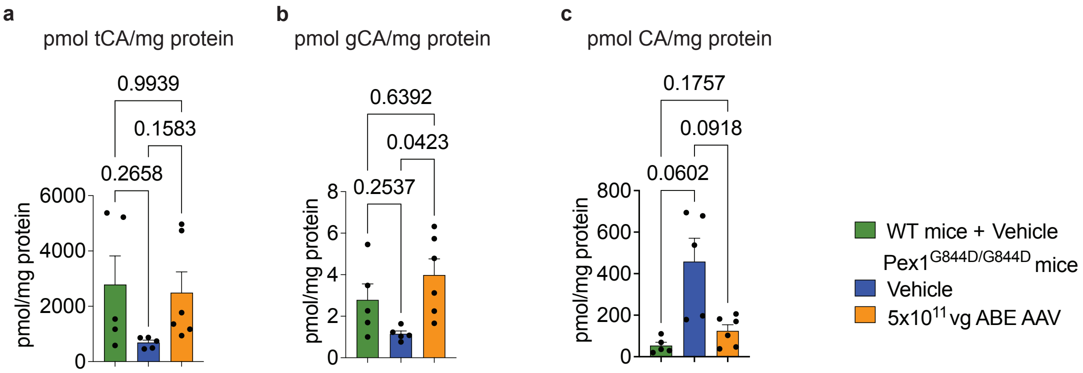

Supplementary Note 4 | Custom python script used for hierarchical cluster analysis of differentially expressed genes in RNA sequencing (related to Fig. 3).

```
import datetime
from pathlib import Path
import re

import matplotlib.pyplot as plt
import numpy as np
import pandas as pd
from pydeseq2.dds import DeseqDataSet
from pydeseq2.ds import DeseqStats
from matplotlib.colors import rgb2hex
from matplotlib import colormaps as CMAPS
from scipy.spatial.distance import pdist
import scipy.cluster.hierarchy as sch
import matplotlib.ticker as tkr
from mpl_toolkits.axes_grid1 import make_axes_locatable


GENE_RE = re.compile('>(.+?) .?\[gene=(.+?)\].+')
GENE_MAPPING = {}
GENOME = 'indexs/GRCm39_CDS.fna'

with open(GENOME, 'r') as f:
    for line in f:
        if line[0] == '>':
            m = GENE_RE.match(line.strip())
            GENE_MAPPING[m.group(1)] = m.group(2)

CCDS = 'CCDS.current.txt'
ccds_df = pd.read_csv(CCDS, sep='\t')
CCDS_GENES = set(ccds_df['gene'].tolist())

SAMPLE_RE = re.compile('(\d+)_(\d+wks)_(dose[A-E])(mut|wt)?_(.+)')

SAMPLES = list(Path('kallisto_out').iterdir())
SAMPLES = [(x, SAMPLE_RE.match(x.name)) for x in SAMPLES]
SAMPLES = [(x, int(m.group(1)), m.group(2), m.group(3), m.group(4), m.group(5)) for x, m in SAMPLES]
SAMPLES.sort(key=lambda x: x[1])

counts = {}
tpms = {}
names = []
times = []
doses = []
groups = []
ids = []
for p, i, time, dose, group, id_ in SAMPLES:
    name = f'{i}_{id_}'
    df = pd.read_csv(p / 'abundance.tsv', sep='\t', index_col=0)
    counts[name] = df['est_counts']
    tpms[p.name] = df['tpm']
    names.append(name)
```

```
    times.append(time)
    doses.append(dose)
    groups.append(group if group is not None else 'mut')
    ids.append(id_)

count_df = pd.DataFrame.from_dict(counts)
count_df['gene'] = count_df.index.map(lambda x: GENE_MAPPING[x])
count_df = count_df.groupby('gene').sum().apply(lambda x: round(x, 0)).astype(np.int64)
count_df = count_df.transpose()
passing_count = count_df.sum(axis=1) > 500000
count_df = count_df[[x for x in count_df.columns if x in CCDS_GENES]].copy()
count_df = count_df.loc[passing_count]
meta_df = pd.DataFrame({'name': names, 'time': times, 'dose': doses, 'group': groups, 'id':
ids}).set_index('name')
meta_df = meta_df.loc[passing_count]
tpm_df = pd.DataFrame.from_dict(tpms)
tpm_df['gene'] = tpm_df.index.map(lambda x: GENE_MAPPING[x])
tpm_df = tpm_df.groupby('gene').sum()
tpm_df.to_csv('DE_out/TPM_estimates.csv')
# Load sex data
sex_df = pd.read_csv('sex_info_240201.csv')
sex_df['id'] = sex_df['SampleName'].str.split('_', n=2, expand=True)[2]
sex_df = sex_df.set_index('id', drop=True)
sex_df = sex_df[['sex']]
# Add sex data to meta_df
meta_df = meta_df.join(sex_df, on='id')
meta_df['group-dose'] = meta_df['group'] + '-' + meta_df['dose']
meta_df.head()

manual_df = pd.read_csv('manual_genes.csv')
wk6_strict = manual_df['6wk-strict'].dropna()
wk16_strict = manual_df['16wk-strict'].dropna()
wk6_loose = manual_df['6wk-large'].dropna()
wk16_loose = manual_df['16wk-large'].dropna()

from scipy.spatial.distance import pdist
import scipy.cluster.hierarchy as sch
import matplotlib.ticker as tkr
from mpl_toolkits.axes_grid1 import make_axes_locatable

def make_linkage(x, pdists):
    n = x.shape[0]
    lnk = np.zeros((n - 1, 4))
    clusters = [[i] for i in range(n)]
    cluster_idxs = [i for i in range(n)]
    for iteration in range(n - 1):
        dists = np.zeros(len(clusters) - 1)
        for idx in range(len(clusters) - 1):
            max_dist = 0
            c_i, c_j = clusters[idx], clusters[idx + 1]
            for i in c_i:
                for j in c_j:
                    pd_idx = n * i + j - ((i + 2) * (i + 1)) // 2
                    max_dist = max(max_dist, pdists[pd_idx])
            dists[idx] = max_dist
        min_idx = np.argmin(dists)
```

```
        new_cluster = clusters[min_idx] + clusters[min_idx + 1]
        lnk[iteration, :] = [cluster_idxs[min_idx], cluster_idxs[min_idx + 1], dists[min_idx], len(new_cluster)]
        if min_idx + 2 < len(clusters):
            clusters = clusters[:min_idx] + [new_cluster] + clusters[min_idx + 2:]
            cluster_idxs = cluster_idxs[:min_idx] + [n + iteration] + cluster_idxs[min_idx + 2:]
        else:
            clusters = clusters[:-2] + [new_cluster]
            cluster_idxs = cluster_idxs[:-2] + [n + iteration]
    return lnk

# Compute Z-scores
meta_df = meta_df.reset_index()
meta_df[['i', 'id']] = meta_df['name'].str.split('_', n=1, expand=True)
meta_df = meta_df.set_index('name', drop=True)
meta_df['groupE'] = ''
meta_df['groupE'] = meta_df['groupE'].where(meta_df['dose'] != 'doseE', meta_df['group'])
meta_df['tpm_col'] = meta_df['i'] + '_' + meta_df['time'] + '_' + meta_df['dose'] + meta_df['groupE'] + '_' +
meta_df['id']
meta_df['label'] = meta_df['time'] + ' ' + meta_df['dose'] + meta_df['groupE'] + ' (' +
meta_df['sex'].str.slice(0, 1) + ')'

meta_6 = meta_df[meta_df['time'] == '6wks']
meta_16 = meta_df[meta_df['time'] == '16wks']

x = tpm_df

x_6 = x[meta_6['tpm_col']]
mu_6 = x_6.mean(axis=1)
std_6 = x_6.std(axis=1)
x_6 = x_6.sub(mu_6, axis=0).div(std_6, axis=0)

x_16 = x[meta_16['tpm_col']]
mu_16 = x_16.mean(axis=1)
std_16 = x_16.std(axis=1)
x_16 = x_16.sub(mu_16, axis=0).div(std_16, axis=0)

def plot_hierarchical(x, meta, gene_list, title):
    x = x.loc[gene_list]
    mu = x.mean(axis=1)
    std = x.std(axis=1)
    x = x.sub(mu, axis=0).div(std, axis=0)
    # Order genes hierarchically
    pdists = pdist(x.values, 'euclidean')
    gene_lnk = sch.linkage(pdists)
    gene_lnk = sch.optimal_leaf_ordering(gene_lnk, pdists)
    gene_idxs = sch.leaves_list(gene_lnk)
    x = x.iloc[gene_idxs]
    # Sort in order we want
    sorted = (meta.replace('wt', '0wt')
              .sort_values(['time', 'group', 'dose', 'sex'])
              .replace('0wt', 'wt'))
    # Hierarchical "clustering"
    x = x.transpose()
    x = x.reset_index()
    x[['i', 'time', 'dose', 'id']] = x['index'].str.split('_', n=3, expand=True)
    x = x.set_index(x['i'] + '_' + x['id'], drop=True)
```

```
    x = x.drop(columns=['index', 'i', 'time', 'dose', 'id'])
    x = x.loc[sorted.index]
    x = x.values
    pdists = pdist(x, 'euclidean')
    lnk = make_linkage(x, pdist(x))
    # Plotting
    fig, (ax1, ax2) = plt.subplots(2, figsize=(6, 8), dpi=300, height_ratios=[1, 3])
    sch.dendrogram(lnk, ax=ax1, no_labels=True, color_threshold=0, above_threshold_color='k')
    im = ax2.imshow(x.transpose(), aspect='auto', cmap='bwr')
    im.set_clim(vmin=-3, vmax=3)
    ax1.xaxis.set_major_locator(tkr.NullLocator())
    ax1.yaxis.set_major_locator(tkr.NullLocator())
    ax2.yaxis.set_major_locator(tkr.NullLocator())
    ax2.set_xticks(np.arange(len(sorted)), sorted['label'], rotation='vertical')
    plt.tight_layout()
    plt.savefig(f'hier_{title}.pdf')
    plt.show()

plot_hierarchical(x_6, meta_6, wk6_strict, 'wk6_strict')
plot_hierarchical(x_16, meta_16, wk16_strict, 'wk16_strict')
plot_hierarchical(x_6, meta_6, wk6_loose, 'wk6_loose')
plot_hierarchical(x_16, meta_16, wk16_loose, 'wk16_loose')

# Order genes hierarchically
pdists = pdist(x_6.values, 'euclidean')
gene_lnk = sch.linkage(pdists)
gene_lnk = sch.optimal_leaf_ordering(gene_lnk, pdists)
gene_idxs = sch.leaves_list(gene_lnk)
x_6 = x_6.iloc[gene_idxs]
# Sort in order we want
sorted_6 = (meta_6.replace('wt', '0wt')
            .sort_values(['time', 'group', 'dose', 'sex'])
            .replace('0wt', 'wt'))
# Hierarchical "clustering"
x_6 = x_6.transpose()
x_6 = x_6.reset_index()
x_6[['i', 'time', 'dose', 'id']] = x_6['index'].str.split('_', n=3, expand=True)
x_6 = x_6.set_index(x_6['i'] + '_' + x_6['id'], drop=True)
x_6 = x_6.drop(columns=['index', 'i', 'time', 'dose', 'id'])
x_6 = x_6.loc[sorted_6.index]
x_6 = x_6.values
pdists = pdist(x_6, 'euclidean')
lnk = make_linkage(x_6, pdist(x_6))

fig, (ax1, ax2) = plt.subplots(2, figsize=(6, 8), dpi=100, height_ratios=[1, 3])
# fig.subplots_adjust(0, 0, 1, 1, 0, 0)
# div = make_axes_locatable(ax1)
# cax = div.append_axes('top', size='5%', pad=0.05)
sch.dendrogram(lnk, ax=ax1, no_labels=True, color_threshold=0, above_threshold_color='k')
im = ax2.imshow(x_6.transpose(), aspect='auto', cmap='bwr')
im.set_clim(vmin=-3, vmax=3)
ax1.xaxis.set_major_locator(tkr.NullLocator())
ax1.yaxis.set_major_locator(tkr.NullLocator())
# ax1.set_ylim(2, 13)
# ax2.xaxis.set_major_locator(tkr.NullLocator())
ax2.yaxis.set_major_locator(tkr.NullLocator())
```

```
ax2.set_xticks(np.arange(len(sorted_6)), sorted_6['label'], rotation='vertical')
# ax2.axhline(69)
# plt.colorbar(im, cax=cax, orientation='horizontal')
plt.tight_layout()
# plt.savefig(f'hierarchical_{now}.pdf')
plt.show()
```

Supplementary Note 5 | Custom python script used for lipids analysis

R-script option for generating volcano plots in Fig. 2 and Extended Data Fig. 3d (if not using PRISM).

```
##############
#!!!The below information requires user input!!!:
##############
#Specify the age of the samples:
age.id <- "6wk"

#Specify the tissue type:
tissue.id <- "plasma"

#Read in data file (this should be a csv file that is processed (specifically formatted) from the original
Excel file!)
data.in <- read.table("C:\Users\piecpa\Desktop\6_weeks_329-Plasma_CleanData.csv", sep=",", head=T,
fill=T, check.names = F, comment.char = ";", na.strings = "")

#Specify the directory where files are to be saved:
dir.out <- "C:\Users\piecpa\Desktop\Volcano_plots"

##############
##############

##############
#The below information does NOT require user input:
##############
#Required libraries
library(PMCMRplus)
library(PMCMR)
library(rstatix)
library(dunn.test)
library(ff)
library(plotrix)
library(ggplot2)
library(ggrepel)

#Optional libraries (for troubleshooting):
#library(reshape2)
#library(tidyr)
#library(tidyverse)
#library(ggpubr)
#library(plyr)
#library(FSA)
#library(data.table)
#library(readxl)
#library(dplyr)
#library(hrbrthemes)
#library(RColorBrewer)


#Turn off scientific notation
options(scipen=999)
```

```
#Remove any row where the second column has no data
data.str <- data.in[!(is.na(data.in[,2])),]

#Assign the column information to a vector per row's data
animal.id <- data.str[1,] #Row 1 should always be the animal ID
lipid.mg  <- data.str[2,] #Row 2 should always be the lipid amount (this is currently not used for
plot/analysis)
dose.id   <- data.str[3,] #Row 3 should always be the dose (aka the sample group)
geno.id   <- data.str[4,] #Row 4 should always be the genotype

#Assign a vector to contain the first column information
row.lab <- data.frame(data.str[,1])

#Create dataset per each dose (sample group) category
B.spec <- data.str[,dose.id == "B"]
B.spec <- cbind(row.lab, B.spec)

C.spec <- data.str[,dose.id == "C"]
C.spec <- cbind(row.lab, C.spec)

D.spec <- data.str[,dose.id == "D"]
D.spec <- cbind(row.lab, D.spec)

E.wt.spec <- data.str[,((dose.id == "E") & (geno.id == "wild"))]
E.wt.spec <- cbind(row.lab, E.wt.spec)

E.mu.spec <- data.str[,((dose.id == "E") & (geno.id == "hom"))]
E.mu.spec <- cbind(row.lab, E.mu.spec)


##############
#Log2 fold Change Calculation
##############
#Calculate the average across all samples within the dose group
#-Rows 1 to 4 are label information and need to be excluded
#-Column 1 is label information and needs to be excluded
B.avg    <- round(rowMeans(sapply(B.spec[c(-1:-4),-1], as.numeric, na.rm=T)), 2)
C.avg    <- round(rowMeans(sapply(C.spec[c(-1:-4),-1], as.numeric, na.rm=T)), 2)
D.avg    <- round(rowMeans(sapply(D.spec[c(-1:-4),-1], as.numeric, na.rm=T)), 2)
E.wt.avg <- round(rowMeans(sapply(E.wt.spec[c(-1:-4),-1], as.numeric, na.rm=T)), 2) #This sample is the
control
E.mu.avg <- round(rowMeans(sapply(E.mu.spec[c(-1:-4),-1], as.numeric, na.rm=T)), 2)

#Calculate the fold change between an experimental dose and the control dose
B.fc    <- round(log2(B.avg/E.wt.avg), 2)
C.fc    <- round(log2(C.avg/E.wt.avg), 2)
D.fc    <- round(log2(D.avg/E.wt.avg), 2)
E.wt.fc <- round(log2(E.wt.avg/E.wt.avg), 2) #Added for tracking control sample
E.mu.fc <- round(log2(E.mu.avg/E.wt.avg), 2)

#Create label information for each average vector
B.lab    <- c("ALL_B",    "ALL_B",    "B", "wild")
C.lab    <- c("ALL_C",    "ALL_C",    "C", "wild")
D.lab    <- c("ALL_D",    "ALL_D",    "D", "wild")
E.wt.lab <- c("ALL_E_wt", "ALL_E_wt", "E", "wild")
E.mu.lab <- c("ALL_E_mu", "ALL_E_mu", "E", "hom")
```

```
#Create a vector of dose average with the labels
B.avg.vec    <- c(B.lab,    B.avg)
C.avg.vec    <- c(C.lab,    C.avg)
D.avg.vec    <- c(D.lab,    D.avg)
E.wt.avg.vec <- c(E.wt.lab, E.wt.avg)
E.mu.avg.vec <- c(E.mu.lab, E.mu.avg)

#Create a vector of the fold change with the labels
B.fc.vec    <- c(B.lab,    B.fc)
C.fc.vec    <- c(C.lab,    C.fc)
D.fc.vec    <- c(D.lab,    D.fc)
E.wt.fc.vec <- c(E.wt.lab, E.wt.fc) #Added for tracking control sample
E.mu.fc.vec <- c(E.mu.lab, E.mu.fc)

#Add the average and fold change data to the dose subset dataframe
B.df    <- cbind(B.spec,    data.frame(B.avg.vec, B.fc.vec))
C.df    <- cbind(C.spec,    data.frame(C.avg.vec, C.fc.vec))
D.df    <- cbind(D.spec,    data.frame(D.avg.vec, D.fc.vec))
E.wt.df <- cbind(E.wt.spec, data.frame(E.wt.avg.vec, E.wt.fc.vec)) #Added for tracking control sample
E.mu.df <- cbind(E.mu.spec, data.frame(E.mu.avg.vec, E.mu.fc.vec))

##############
#Create a dataframe where lipids are the columns and samples are rows
##############
#Assign the dataframe labels to be applied to the transposed dataframe
df.lab <- data.str$`Sample Name`

#Transpose the lipid dataframe
data.t <- as.data.frame(t(data.str[,-1]))
colnames(data.t) <- df.lab

#Create a dose category which is dose + genotype
data.t$DG <- paste(data.t$Dose, "_", data.t$Genotype, sep="")
data.t$DG <- as.factor(data.t$DG)

#Rearrange the factor levels so that E_wild (the control) is listed first
#The "ManyToOne" Dunnett test uses the first listed factor as the control for which all other groups are
compared against.
data.t$DG <- factor(data.t$DG, levels = c("E_wild", "B_hom", "C_hom", "D_hom", "E_hom"))

#Run loop for each lipid and create a dataframe that stores all the p-values for the four comparisons
i <- 1

for(i in 1:(ncol(data.t)-5)){
  #####
  #Obtain the chemical and traditional name of the lipid from the column label
  #-If a traditional name isn't present, the chemical name will be assigned as the traditional name
  iter.id <- names(data.t)[(i+4)]

  split.id <- strsplit(iter.id, " - ", fixed=F)

  chem.id <- split.id[[1]][1]
  name.id <- split.id[[1]][2]

  if(is.na(name.id)){
```

```
    name.id <- chem.id
  }
  #####

  dunn.res <- kwManyOneDunnTest(as.numeric(data.t[,(i+4)]), data.t$DG, p.adjust.method = "bonferroni")

  #Append the test results to the labels
  dunn.df <- data.frame(iter.id, chem.id, name.id, t(dunn.res$p.value))
  row.names(dunn.df) <- NULL

  #Concatenate all results into one master results dataframe
  if(i == 1){
    dunn.res.df <- dunn.df
  }else{
    dunn.res.df <- rbind(dunn.res.df, dunn.df)
  }
}

names(dunn.res.df) <- c("Lipid_ID", "Chem_ID", "Name_ID", "B_PVAL", "C_PVAL", "D_PVAL",
"E_PVAL")

#Merge the average and fold change data to the p-values
B.df.sub    <- B.df[c(5:nrow(B.df)),       c(1, (ncol(B.df)-1):(ncol(B.df)))]
names(B.df.sub) <- c("Lipid_ID", "B_AVG", "B_FC")

C.df.sub    <- C.df[c(5:nrow(C.df)),       c(1, (ncol(C.df)-1):(ncol(C.df)))]
names(C.df.sub) <- c("Lipid_ID", "C_AVG", "C_FC")

D.df.sub    <- D.df[c(5:nrow(D.df)),       c(1, (ncol(D.df)-1):(ncol(D.df)))]
names(D.df.sub) <- c("Lipid_ID", "D_AVG", "D_FC")

E.wt.df.sub <- E.wt.df[c(5:nrow(E.wt.df)), c(1, (ncol(E.wt.df)-1):(ncol(E.wt.df)))]
names(E.wt.df.sub) <- c("Lipid_ID", "E_WT_AVG", "E_WT_FC")

E.mu.df.sub <- E.mu.df[c(5:nrow(E.mu.df)), c(1, (ncol(E.mu.df)-1):(ncol(E.mu.df)))]
names(E.mu.df.sub) <- c("Lipid_ID", "E_MU_AVG", "E_MU_FC")

merge.1 <- merge(B.df.sub, C.df.sub,    by.x = "Lipid_ID", by.y = "Lipid_ID", all=T)
merge.2 <- merge(merge.1,  D.df.sub,    by.x = "Lipid_ID", by.y = "Lipid_ID", all=T)
merge.3 <- merge(merge.2,  E.wt.df.sub, by.x = "Lipid_ID", by.y = "Lipid_ID", all=T)
merge.4 <- merge(merge.3,  E.mu.df.sub, by.x = "Lipid_ID", by.y = "Lipid_ID", all=T)

merge.all <- merge(merge.4, dunn.res.df, by.x = "Lipid_ID", by.y = "Lipid_ID")

#Rearrange the columns in merge.all so that the results are listed by group in alphabetical order
merge.fin <- merge.all[, c("Lipid_ID", "Chem_ID", "Name_ID",
                           "B_AVG",    "B_FC",    "B_PVAL",
                           "C_AVG",    "C_FC",    "C_PVAL",
                           "D_AVG",    "D_FC",    "D_PVAL",
                           "E_MU_AVG", "E_MU_FC", "E_PVAL",
                           "E_WT_AVG", "E_WT_FC")]

#Replace NaN, Inf, and -Inf values with zeroes
#-This occurs when a lipid has a 0 reading, as it will produce a NaN/Inf fold change
merge.fin.1 <- lapply(merge.fin,   function(x) as.character(gsub("-Inf", "0", x)))
```

```
merge.fin.2 <- lapply(merge.fin.1, function(x) as.character(gsub("Inf", "0", x)))

merge.fin.3 <- lapply(merge.fin.2, function(x) as.character(gsub("NaN", "0", x)))

merge.fin.df <- as.data.frame(merge.fin.3)

merge.fin <- merge.fin.df

#Save the merged final results to the output directory
write.table(merge.fin, paste(dir.out, "/", tissue.id, "_", age.id, "_AVG_FC_PVAL.txt", sep=""), sep="\t",
row=F, quote=F)

#Identify the min and max fold change to ensure the axis range is the same across all plots
x.max <- ceiling(max(as.numeric(c(merge.fin$B_FC, merge.fin$C_FC, merge.fin$D_FC,
merge.fin$E_MU_FC)), na.rm=T)) + 1
x.min <- floor(min(as.numeric(c(merge.fin$B_FC, merge.fin$C_FC, merge.fin$D_FC,
merge.fin$E_MU_FC)), na.rm=T))

y.max <- ceiling(-log10(min(as.numeric(c(merge.fin$B_PVAL, merge.fin$C_PVAL, merge.fin$D_PVAL,
merge.fin$E_PVAL)), na.rm=T))) + 1
y.min <- 0

##############
#Volcano plots
##############
#Create a volcano plot for each pairwise comparison (e.g., B vs. E_wt, etc.) that corresponds to:
#1-Only the Lipid_IDs that are NOT "total..." or "sum..."
#2-Only the Lipid_IDs that are "total..." or "sum..."

i <- 1
for(i in 1:4){
  if(i == 1){
    plot.data <- merge.fin[, c("Lipid_ID", "Chem_ID", "Name_ID", "B_AVG", "B_FC", "B_PVAL")]
    group.id <- "B"
    file.id <- group.id
  }else if(i == 2){
    plot.data <- merge.fin[, c("Lipid_ID", "Chem_ID", "Name_ID", "C_AVG", "C_FC", "C_PVAL")]
    group.id <- "C"
    file.id <- group.id
  }else if(i == 3){
    plot.data <- merge.fin[, c("Lipid_ID", "Chem_ID", "Name_ID", "D_AVG", "D_FC", "D_PVAL")]
    group.id <- "D"
    file.id <- group.id
  }else if(i == 4){
    plot.data <- merge.fin[, c("Lipid_ID", "Chem_ID", "Name_ID", "E_MU_AVG", "E_MU_FC", "E_PVAL")]
    group.id <- "E mut"
    file.id  <- "E_mut"
  }

  names(plot.data) <- c("Lipid_ID", "Chem_ID", "Name_ID", "AVG", "FC", "PVAL")

  #Set types
  plot.data$AVG  <- as.numeric(plot.data$AVG)
  plot.data$FC   <- as.numeric(plot.data$FC)
  plot.data$PVAL <- as.numeric(plot.data$PVAL)
```

```
 j <- 1
 for(j in 1:2){
   #Subset the lipid ID (j == 1: exclude "total..." and "sum"; j == 2: include only "total..." and "sum")
   if(j == 1){
     plot.rem <- c(-grep("sum", plot.data$Lipid_ID), -grep("total", plot.data$Lipid_ID))
     plot.final <- plot.data[plot.rem,]
     data.id <- "all_lipids"
   }else if(j == 2){
     plot.rem <- c(-grep("sum", plot.data$Lipid_ID), -grep("total", plot.data$Lipid_ID))
     plot.final <- plot.data[-plot.rem,]
     data.id <- "total_lipids"
   }

   #Invert the p-values so that higher numbers correspond to higher significance
   plot.final$logPVAL <- -log10(plot.final$PVAL)
   plot.final$logPVAL  <- as.numeric(plot.final$logPVAL)

   #Identify the min and max fold change for the subset data (to determine which point colors are needed)
   x.max.pvo <- ceiling(max(as.numeric(plot.final$FC)))
   x.min.pvo <- floor(min(as.numeric(plot.final$FC)))

   plot.pos <- subset(plot.final, ((plot.final$FC >= 1)  & (plot.final$logPVAL > 1.30103))) #Positive FC
subset
   plot.neg <- subset(plot.final, ((plot.final$FC <= -1) & (plot.final$logPVAL > 1.30103))) #Negative FC
subset

   #####
   #Plotting function (plot object = "pvo")
   pvo <- ggplot(plot.final)

   #Main plot function (set non-significant points (p-value >= 0.05 & FC < |1|) to "gray60")
   pvo <- pvo + geom_point(data = plot.final,  aes(x=FC, y=logPVAL), color = "gray60", size = 3.0)

   #Plot points and colors
   if((nrow(plot.pos) > 0) & (nrow(plot.neg) > 0)){ #FC range spans both positive and negative numbers
     pvo <- pvo + geom_point(data = plot.pos, aes(x=FC, y=logPVAL, color = "red"),    size = 3.0)
     pvo <- pvo + geom_point(data = plot.neg, aes(x=FC, y=logPVAL, color = "green3"), size = 3.0)
     pvo <- pvo + scale_color_identity(guide = "legend",
                                 name = "P < 0.05:",
                                 breaks = c("", "red", "green3"),
                                 labels = c("", "FC > 1", "FC < -1"))

     pvo <- pvo + geom_text_repel(data = plot.pos, aes(x = FC, y = logPVAL, label = Chem_ID), size = 5,
max.overlaps = Inf)
     pvo <- pvo + geom_text_repel(data = plot.neg, aes(x = FC, y = logPVAL, label = Chem_ID), size = 5,
max.overlaps = Inf)

   }else if((nrow(plot.pos) == 0) & (nrow(plot.neg) > 0)){ #FC spans only negative numbers
     pvo <- pvo + geom_point(data = plot.neg, aes(x=FC, y=logPVAL, color = "green3"), size = 3.0)
     pvo <- pvo + scale_color_identity(guide = "legend",
                                 name = "P < 0.05:",
                                 breaks = c("", "green3"),
                                 labels = c("", "FC < -1"))

     pvo <- pvo + geom_text_repel(data = plot.neg, aes(x = FC, y = logPVAL, label = Chem_ID), size = 5,
max.overlaps = Inf)
```

```
}else if((nrow(plot.pos) > 0) & (nrow(plot.neg) == 0)){ #FC spans only positive numbers
  pvo <- pvo + geom_point(data = plot.pos, aes(x=FC, y=logPVAL, color = "red"), size = 3.0)
  pvo <- pvo + scale_color_identity(guide = "legend",
                                    name = "P < 0.05:",
                                    breaks = c("", "red"),
                                    labels = c("", "FC > 1"))

  pvo <- pvo + geom_text_repel(data = plot.pos, aes(x = FC, y = logPVAL, label = Chem_ID), size = 5,
max.overlaps = Inf)
}

#Plot main theme and titles
pvo <- pvo + theme_bw()
pvo <- pvo + labs(x = "Log(2) Fold Change", y = "-Log(10) P value")
pvo <- pvo + ggtitle(paste(group.id, " vs. E wt, ", tissue.id, ", ", age.id, sep=""))
pvo <- pvo + theme(plot.title = element_text(hjust = 0.25))

#Black, 1-point, solid lines demarking the Y and X axes:
pvo <- pvo + geom_hline(yintercept = 0,       col = "black",  linetype = "solid",  linewidth = 1.0, alpha =
1.00) #Y-axis divider
pvo <- pvo + geom_vline(xintercept = 0,       col = "black",  linetype = "solid",  linewidth = 1.0, alpha =
1.00) #X-axis divider

#Additional lines demarking thresholds of interest:
pvo <- pvo + geom_vline(xintercept = 1,       col = "red",    linetype = "dashed", linewidth = 1.5, alpha =
0.75) #FC divider
pvo <- pvo + geom_vline(xintercept = -1,      col = "green3", linetype = "dashed", linewidth = 1.5, alpha
= 0.75) #FC divider
pvo <- pvo + geom_hline(yintercept = 1.30103, col = "black",  linetype = "dashed", linewidth = 1.5,
alpha = 0.75) #logPVAL divider

#Set the x and y axis limits
#pvo <- pvo + xlim(round(x.min/2)*2, round(x.max/2)*2)
#pvo <- pvo + ylim(0, round(y.max/2)*2)

#Set axis tick marks to appear every 2 units
pvo <- pvo + scale_x_continuous(breaks = seq(round(x.min/2)*2, round(x.max/2)*2, by = 2),
                                #n.breaks = length(seq(round(x.min/2)*2, round(x.max/2)*2, by = 2)),
                                #labels = seq(round(x.min/2)*2, round(x.max/2)*2, by = 2),
                                limits = c(round(x.min/2)*2, round(x.max/2)*2))

pvo <- pvo + scale_y_continuous(breaks = seq(y.min, round(y.max/2)*2, by = 2),
                                #n.breaks = length(seq(y.min, round(y.max/2)*2, by = 2)),
                                #labels = seq(y.min, round(y.max/2)*2, by = 2),
                                limits = c(0, round(y.max/2)*2))

#Theme settings part 1 (borders)
pvo <- pvo + theme(panel.border = element_blank(),
                   #panel.grid.major = element_blank(), panel.grid.minor = element_blank(),
                   panel.background = element_blank(), axis.line = element_line(colour = "black"))

#Theme settings part 2 (legend format)
pvo <- pvo + theme(legend.position = "bottom", legend.justification = c(0,0),
                   legend.key = element_rect(fill = alpha("white", 100)),
                   legend.background = element_rect(fill=alpha("white", 0.0)),
```

```
                    legend.box.background = element_rect(color = "black", fill=NA),
                    legend.text  = element_text(size=12),
                    legend.title = element_text(size=12))

    #Theme settings part 3 (axes text settings)
    pvo <- pvo + theme(axis.text=element_text(size=16), axis.title=element_text(size=18))

    #Theme settings part 4 (center main title, set to 18 font size)
    pvo <- pvo + theme(plot.title = element_text(hjust = 0.5, size = 18))

    #Guide settings
    pvo <- pvo + guides(colour = guide_legend(title.hjust = 0.5, override.aes = list(size = 2)))

    #Run volcano plot object to be saved by ggsave
    pvo

    #Save the volcano plot
    ggsave(paste(dir.out, "/", file.id, "_vs_E_wt_", tissue.id, "_", age.id, "_", data.id,".pdf", sep=""), width=7,
height=7)
  }
}
```

R-script used to generate Volcano Plot Figure 4, Extended Data Fig. 1 and 8 (if not using PRISM).

```
##############
#!!!The below information requires user input!!!:
##############
#Specify the age of the samples:
age.id <- "16wk"

#Specify the tissue type:
tissue.id <- "liver"

#Read in data file (this should be a csv file that is processed (specifically formatted) from the original
Excel file!)
data.in <- read.table("C:\Users\piecpa\Desktop\319_liver_TFLA_16wks.csv", sep=",", head=T, fill=T,
check.names = F, comment.char = ";", na.strings = "")

#Specify the directory where files are to be saved:
dir.out <- "C:\Users\piecpa\Desktop\Volcano_plots"

##############
##############

##############
#The below information does NOT require user input:
##############
#Required libraries
library(PMCMRplus)
library(PMCMR)
library(rstatix)
library(dunn.test)
library(ff)
library(plotrix)
library(ggplot2)
library(ggrepel)

#Optional libraries (for troubleshooting):
#library(reshape2)
#library(tidyr)
#library(tidyverse)
#library(ggpubr)
#library(plyr)
#library(FSA)
#library(data.table)
#library(readxl)
#library(dplyr)
#library(hrbrthemes)
#library(RColorBrewer)


#Turn off scientific notation
options(scipen=999)

#Remove any row where the second column has no data
data.str <- data.in[!(is.na(data.in[,2])),]
```

```
#Assign the column information to a vector per row's data
animal.id <- data.str[1,] #Row 1 should always be the animal ID
lipid.mg  <- data.str[2,] #Row 2 should always be the lipid amount (this is currently not used for
plot/analysis)
dose.id   <- data.str[3,] #Row 3 should always be the dose (aka the sample group)
geno.id   <- data.str[4,] #Row 4 should always be the genotype

#Assign a vector to contain the first column information
row.lab <- data.frame(data.str[,1])

#Create dataset per each dose (sample group) category
B.spec <- data.str[,dose.id == "B"]
B.spec <- cbind(row.lab, B.spec)

#group_omit C.spec <- data.str[,dose.id == "C"]
#group_omit C.spec <- cbind(row.lab, C.spec)

#group_omit D.spec <- data.str[,dose.id == "D"]
#group_omit D.spec <- cbind(row.lab, D.spec)

E.wt.spec <- data.str[,((dose.id == "E") & (geno.id == "wild"))]
E.wt.spec <- cbind(row.lab, E.wt.spec)

E.mu.spec <- data.str[,((dose.id == "E") & (geno.id == "hom"))]
E.mu.spec <- cbind(row.lab, E.mu.spec)


##############
#Log2 fold Change Calculation
##############
#Calculate the average across all samples within the dose group
#-Rows 1 to 4 are label information and need to be excluded
#-Column 1 is label information and needs to be excluded
B.avg    <- round(rowMeans(sapply(B.spec[c(-1:-4),-1], as.numeric, na.rm=T)), 2)
#group_omit C.avg    <- round(rowMeans(sapply(C.spec[c(-1:-4),-1], as.numeric, na.rm=T)), 2)
#group_omit D.avg    <- round(rowMeans(sapply(D.spec[c(-1:-4),-1], as.numeric, na.rm=T)), 2)
E.wt.avg <- round(rowMeans(sapply(E.wt.spec[c(-1:-4),-1], as.numeric, na.rm=T)), 2) #This sample is the
control
E.mu.avg <- round(rowMeans(sapply(E.mu.spec[c(-1:-4),-1], as.numeric, na.rm=T)), 2)

#Calculate the fold change between an experimental dose and the control dose
B.fc    <- round(log2(B.avg/E.wt.avg), 2)
#group_omit C.fc    <- round(log2(C.avg/E.wt.avg), 2)
#group_omit D.fc    <- round(log2(D.avg/E.wt.avg), 2)
E.mu.fc <- round(log2(E.mu.avg/E.wt.avg), 2)
E.wt.fc <- round(log2(E.wt.avg/E.wt.avg), 2) #Added for tracking control sample

#Create label information for each average vector
B.lab    <- c("ALL_B",    "ALL_B",    "B", "wild")
#group_omit C.lab    <- c("ALL_C",    "ALL_C",    "C", "wild")
#group_omit D.lab    <- c("ALL_D",    "ALL_D",    "D", "wild")
E.wt.lab <- c("ALL_E_wt", "ALL_E_wt", "E", "wild")
E.mu.lab <- c("ALL_E_mu", "ALL_E_mu", "E", "hom")

#Create a vector of dose average with the labels
B.avg.vec    <- c(B.lab,    B.avg)
```

```
#group_omit C.avg.vec    <- c(C.lab,    C.avg)
#group_omit D.avg.vec    <- c(D.lab,    D.avg)
E.wt.avg.vec <- c(E.wt.lab, E.wt.avg)
E.mu.avg.vec <- c(E.mu.lab, E.mu.avg)

#Create a vector of the fold change with the labels
B.fc.vec    <- c(B.lab,    B.fc)
#group_omit C.fc.vec    <- c(C.lab,    C.fc)
#group_omit D.fc.vec    <- c(D.lab,    D.fc)
E.wt.fc.vec <- c(E.wt.lab, E.wt.fc) #Added for tracking control sample
E.mu.fc.vec <- c(E.mu.lab, E.mu.fc)

#Add the average and fold change data to the dose subset dataframe
B.df    <- cbind(B.spec,    data.frame(B.avg.vec, B.fc.vec))
#group_omit C.df    <- cbind(C.spec,    data.frame(C.avg.vec, C.fc.vec))
#group_omit D.df    <- cbind(D.spec,    data.frame(D.avg.vec, D.fc.vec))
E.wt.df <- cbind(E.wt.spec, data.frame(E.wt.avg.vec, E.wt.fc.vec)) #Added for tracking control sample
E.mu.df <- cbind(E.mu.spec, data.frame(E.mu.avg.vec, E.mu.fc.vec))

##############
#Create a dataframe where lipids are the columns and samples are rows
##############
#Assign the dataframe labels to be applied to the transposed dataframe
df.lab <- data.str$`Sample Name`

#Transpose the lipid dataframe
data.t <- as.data.frame(t(data.str[,-1]))
colnames(data.t) <- df.lab

#Create a dose category which is dose + genotype
data.t$DG <- paste(data.t$Dose, "_", data.t$Genotype, sep="")
data.t$DG <- as.factor(data.t$DG)

#Rearrange the factor levels so that E_wild (the control) is listed first
#The "ManyToOne" Dunnett test uses the first listed factor as the control for which all other groups are
compared against.
#group_omit data.t$DG <- factor(data.t$DG, levels = c("E_wild", "B_hom", "C_hom", "D_hom", "E_hom"))
data.t$DG <- factor(data.t$DG, levels = c("E_wild", "B_hom", "E_hom")) #group_add


#Run loop for each lipid and create a dataframe that stores all the p-values for the four comparisons
i <- 1

for(i in 1:(ncol(data.t)-5)){
  #####
  #Obtain the chemical and traditional name of the lipid from the column label
  #-If a traditional name isn't present, the chemical name will be assigned as the traditional name
  iter.id <- names(data.t)[(i+4)]

  split.id <- strsplit(iter.id, " - ", fixed=F)

  chem.id <- split.id[[1]][1]
  name.id <- split.id[[1]][2]

  if(is.na(name.id)){
    name.id <- chem.id
```

```
  }
  #####

  dunn.res <- kwManyOneDunnTest(as.numeric(data.t[,(i+4)]), data.t$DG, p.adjust.method = "bonferroni")

  #Append the test results to the labels
  dunn.df <- data.frame(iter.id, chem.id, name.id, t(dunn.res$p.value))
  row.names(dunn.df) <- NULL

  #Concatenate all results into one master results dataframe
  if(i == 1){
    dunn.res.df <- dunn.df
  }else{
    dunn.res.df <- rbind(dunn.res.df, dunn.df)
  }
}

#group_omit names(dunn.res.df) <- c("Lipid_ID", "Chem_ID", "Name_ID", "B_PVAL", "C_PVAL",
"D_PVAL", "E_PVAL")
names(dunn.res.df) <- c("Lipid_ID", "Chem_ID", "Name_ID", "B_PVAL", "E_PVAL") #group_add

#Merge the average and fold change data to the p-values
B.df.sub    <- B.df[c(5:nrow(B.df)),       c(1, (ncol(B.df)-1):(ncol(B.df)))]
names(B.df.sub) <- c("Lipid_ID", "B_AVG", "B_FC")

#group_omitC.df.sub    <- C.df[c(5:nrow(C.df)),       c(1, (ncol(C.df)-1):(ncol(C.df)))]
#group_omitnames(C.df.sub) <- c("Lipid_ID", "C_AVG", "C_FC")

#group_omitD.df.sub    <- D.df[c(5:nrow(D.df)),       c(1, (ncol(D.df)-1):(ncol(D.df)))]
#group_omitnames(D.df.sub) <- c("Lipid_ID", "D_AVG", "D_FC")

E.wt.df.sub <- E.wt.df[c(5:nrow(E.wt.df)), c(1, (ncol(E.wt.df)-1):(ncol(E.wt.df)))]
names(E.wt.df.sub) <- c("Lipid_ID", "E_WT_AVG", "E_WT_FC")

E.mu.df.sub <- E.mu.df[c(5:nrow(E.mu.df)), c(1, (ncol(E.mu.df)-1):(ncol(E.mu.df)))]
names(E.mu.df.sub) <- c("Lipid_ID", "E_MU_AVG", "E_MU_FC")

#group_omit merge.1 <- merge(B.df.sub, C.df.sub,    by.x = "Lipid_ID", by.y = "Lipid_ID", all=T)
#group_omit merge.2 <- merge(merge.1,  D.df.sub,    by.x = "Lipid_ID", by.y = "Lipid_ID", all=T)
#group_omit merge.3 <- merge(merge.2,  E.wt.df.sub, by.x = "Lipid_ID", by.y = "Lipid_ID", all=T)
#group_omit merge.4 <- merge(merge.3,  E.mu.df.sub, by.x = "Lipid_ID", by.y = "Lipid_ID", all=T)

merge.1 <- merge(B.df.sub, E.wt.df.sub,   by.x = "Lipid_ID", by.y = "Lipid_ID", all=T) #group_add
merge.4 <- merge(merge.1,  E.mu.df.sub,    by.x = "Lipid_ID", by.y = "Lipid_ID", all=T) #group_add

merge.all <- merge(merge.4, dunn.res.df, by.x = "Lipid_ID", by.y = "Lipid_ID")

#Rearrange the columns in merge.all so that the results are listed by group in alphabetical order
merge.fin <- merge.all[, c("Lipid_ID", "Chem_ID", "Name_ID",
                           "B_AVG",    "B_FC",    "B_PVAL",
#group_omit                "C_AVG",    "C_FC",    "C_PVAL",
#group_omit                "D_AVG",    "D_FC",    "D_PVAL",
                           "E_MU_AVG", "E_MU_FC", "E_PVAL",
                           "E_WT_AVG", "E_WT_FC")]

#Replace NaN, Inf, and -Inf values with zeroes
```

```
#-This occurs when a lipid has a 0 reading, as it will produce a NaN/Inf fold change
merge.fin.1 <- lapply(merge.fin,   function(x) as.character(gsub("-Inf", "0", x)))

merge.fin.2 <- lapply(merge.fin.1, function(x) as.character(gsub("Inf", "0", x)))

merge.fin.3 <- lapply(merge.fin.2, function(x) as.character(gsub("NaN", "0", x)))

merge.fin.df <- as.data.frame(merge.fin.3)

merge.fin <- merge.fin.df

#Save the merged final results to the output directory
write.table(merge.fin, paste(dir.out, "/", tissue.id, "_", age.id, "_AVG_FC_PVAL.txt", sep=""), sep="\t",
row=F, quote=F)

#Identify the min and max fold change to ensure the axis range is the same across all plots
x.max <- ceiling(max(as.numeric(c(merge.fin$B_FC, merge.fin$C_FC, merge.fin$D_FC,
merge.fin$E_MU_FC)), na.rm=T)) + 1
x.min <- floor(min(as.numeric(c(merge.fin$B_FC, merge.fin$C_FC, merge.fin$D_FC,
merge.fin$E_MU_FC)), na.rm=T))

y.max <- ceiling(-log10(min(as.numeric(c(merge.fin$B_PVAL, merge.fin$C_PVAL, merge.fin$D_PVAL,
merge.fin$E_PVAL)), na.rm=T))) + 1
y.min <- 0

##############
#Volcano plots
##############
#Create a volcano plot for each pairwise comparison (e.g., B vs. E_wt, etc.) that corresponds to:
#1-Only the Lipid_IDs that are NOT "total..." or "sum..."
#2-Only the Lipid_IDs that are "total..." or "sum..."

i <- 1
#group_omit for(i in 1:4){
for(i in 1:2){ #group_add
  if(i == 1){
    plot.data <- merge.fin[, c("Lipid_ID", "Chem_ID", "Name_ID", "B_AVG", "B_FC", "B_PVAL")]
    group.id <- "B"
    file.id <- group.id
  }else if(i == 2){
    #group_omit plot.data <- merge.fin[, c("Lipid_ID", "Chem_ID", "Name_ID", "C_AVG", "C_FC",
"C_PVAL")]
    #group_omit group.id <- "C"
    #group_omit file.id <- group.id
    plot.data <- merge.fin[, c("Lipid_ID", "Chem_ID", "Name_ID", "E_MU_AVG", "E_MU_FC", "E_PVAL")]
#group_add
    group.id <- "E mut" #group_add
    file.id  <- "E_mut" #group_add
  }#group_omitelse if(i == 3){
  #group_omit  plot.data <- merge.fin[, c("Lipid_ID", "Chem_ID", "Name_ID", "D_AVG", "D_FC",
"D_PVAL")]
  #group_omit  group.id <- "D"
  #group_omit  file.id <- group.id
  #group_omit}else if(i == 4){
  #group_omit  plot.data <- merge.fin[, c("Lipid_ID", "Chem_ID", "Name_ID", "E_MU_AVG", "E_MU_FC",
"E_PVAL")]
```

```
  #group_omit  group.id <- "E mut"
  #group_omit  file.id  <- "E_mut"
  #group_omit}

  names(plot.data) <- c("Lipid_ID", "Chem_ID", "Name_ID", "AVG", "FC", "PVAL")

  #Set types
  plot.data$AVG  <- as.numeric(plot.data$AVG)
  plot.data$FC   <- as.numeric(plot.data$FC)
  plot.data$PVAL <- as.numeric(plot.data$PVAL)

  j <- 1
  for(j in 1:2){
    #Subset the lipid ID (j == 1: exclude "total..." and "sum"; j == 2: include only "total..." and "sum")
    if(j == 1){
      plot.rem <- c(-grep("sum", plot.data$Lipid_ID), -grep("total", plot.data$Lipid_ID))
      plot.final <- plot.data[plot.rem,]
      data.id <- "all_lipids"
    }else if(j == 2){
      plot.rem <- c(-grep("sum", plot.data$Lipid_ID), -grep("total", plot.data$Lipid_ID))
      plot.final <- plot.data[-plot.rem,]
      data.id <- "total_lipids"
    }

    #Invert the p-values so that higher numbers correspond to higher significance
    plot.final$logPVAL <- -log10(plot.final$PVAL)
    plot.final$logPVAL  <- as.numeric(plot.final$logPVAL)

    #Identify the min and max fold change for the subset data (to determine which point colors are needed)
    x.max.pvo <- ceiling(max(as.numeric(plot.final$FC)))
    x.min.pvo <- floor(min(as.numeric(plot.final$FC)))

    plot.pos <- subset(plot.final, ((plot.final$FC >= 1)  & (plot.final$logPVAL > 1.30103))) #Positive FC
subset
    plot.neg <- subset(plot.final, ((plot.final$FC <= -1) & (plot.final$logPVAL > 1.30103))) #Negative FC
subset

    #####
    #Plotting function (plot object = "pvo")
    pvo <- ggplot(plot.final)

    #Main plot function (set non-significant points (p-value >= 0.05 & FC < |1|) to "gray60")
    pvo <- pvo + geom_point(data = plot.final,  aes(x=FC, y=logPVAL), color = "gray60", size = 3.0)

    #Plot points and colors
    if((nrow(plot.pos) > 0) & (nrow(plot.neg) > 0)){ #FC range spans both positive and negative numbers
      pvo <- pvo + geom_point(data = plot.pos, aes(x=FC, y=logPVAL, color = "red"),    size = 3.0)
      pvo <- pvo + geom_point(data = plot.neg, aes(x=FC, y=logPVAL, color = "green3"), size = 3.0)
      pvo <- pvo + scale_color_identity(guide = "legend",
                                name = "P < 0.05:",
                                breaks = c("", "red", "green3"),
                                labels = c("", "FC > 1", "FC < -1"))

      pvo <- pvo + geom_text_repel(data = plot.pos, aes(x = FC, y = logPVAL, label = Chem_ID), size = 5,
max.overlaps = Inf)
```

```
    pvo <- pvo + geom_text_repel(data = plot.neg, aes(x = FC, y = logPVAL, label = Chem_ID), size = 5,
max.overlaps = Inf)

  }else if((nrow(plot.pos) == 0) & (nrow(plot.neg) > 0)){ #FC spans only negative numbers
    pvo <- pvo + geom_point(data = plot.neg, aes(x=FC, y=logPVAL, color = "green3"), size = 3.0)
    pvo <- pvo + scale_color_identity(guide = "legend",
                                      name = "P < 0.05:",
                                      breaks = c("", "green3"),
                                      labels = c("", "FC < -1"))

    pvo <- pvo + geom_text_repel(data = plot.neg, aes(x = FC, y = logPVAL, label = Chem_ID), size = 5,
max.overlaps = Inf)

  }else if((nrow(plot.pos) > 0) & (nrow(plot.neg) == 0)){ #FC spans only positive numbers
    pvo <- pvo + geom_point(data = plot.pos, aes(x=FC, y=logPVAL, color = "red"), size = 3.0)
    pvo <- pvo + scale_color_identity(guide = "legend",
                                      name = "P < 0.05:",
                                      breaks = c("", "red"),
                                      labels = c("", "FC > 1"))

    pvo <- pvo + geom_text_repel(data = plot.pos, aes(x = FC, y = logPVAL, label = Chem_ID), size = 5,
max.overlaps = Inf)
  }

  #Plot main theme and titles
  pvo <- pvo + theme_bw()
  pvo <- pvo + labs(x = "Log(2) Fold Change", y = "-Log(10) P value")
  pvo <- pvo + ggtitle(paste(group.id, " vs. E wt, ", tissue.id, ", ", age.id, sep=""))
  pvo <- pvo + theme(plot.title = element_text(hjust = 0.25))

  #Black, 1-point, solid lines demarking the Y and X axes:
  pvo <- pvo + geom_hline(yintercept = 0,       col = "black",  linetype = "solid",  linewidth = 1.0, alpha =
1.00) #Y-axis divider
  pvo <- pvo + geom_vline(xintercept = 0,       col = "black",  linetype = "solid",  linewidth = 1.0, alpha =
1.00) #X-axis divider

  #Additional lines demarking thresholds of interest:
  pvo <- pvo + geom_vline(xintercept = 1,       col = "red",    linetype = "dashed", linewidth = 1.5, alpha =
0.75) #FC divider
  pvo <- pvo + geom_vline(xintercept = -1,      col = "green3", linetype = "dashed", linewidth = 1.5, alpha
= 0.75) #FC divider
  pvo <- pvo + geom_hline(yintercept = 1.30103, col = "black",  linetype = "dashed", linewidth = 1.5,
alpha = 0.75) #logPVAL divider

  #Set the x and y axis limits
  #pvo <- pvo + xlim(round(x.min/2)*2, round(x.max/2)*2)
  #pvo <- pvo + ylim(0, round(y.max/2)*2)

  #Set axis tick marks to appear every 2 units
  pvo <- pvo + scale_x_continuous(breaks = seq(round(x.min/2)*2, round(x.max/2)*2, by = 2),
                                  #n.breaks = length(seq(round(x.min/2)*2, round(x.max/2)*2, by = 2)),
                                  #labels = seq(round(x.min/2)*2, round(x.max/2)*2, by = 2),
                                  limits = c(round(x.min/2)*2, round(x.max/2)*2))

  pvo <- pvo + scale_y_continuous(breaks = seq(y.min, round(y.max/2)*2, by = 2),
                                  #n.breaks = length(seq(y.min, round(y.max/2)*2, by = 2)),
```

```
                                  #labels = seq(y.min, round(y.max/2)*2, by = 2),
                                  limits = c(0, round(y.max/2)*2))

    #Theme settings part 1 (borders)
    pvo <- pvo + theme(panel.border = element_blank(),
                    #panel.grid.major = element_blank(), panel.grid.minor = element_blank(),
                    panel.background = element_blank(), axis.line = element_line(colour = "black"))

    #Theme settings part 2 (legend format)
    pvo <- pvo + theme(legend.position = "bottom", legend.justification = c(0,0),
                    legend.key = element_rect(fill = alpha("white", 100)),
                    legend.background = element_rect(fill=alpha("white", 0.0)),
                    legend.box.background = element_rect(color = "black", fill=NA),
                    legend.text  = element_text(size=12),
                    legend.title = element_text(size=12))

    #Theme settings part 3 (axes text settings)
    pvo <- pvo + theme(axis.text=element_text(size=16), axis.title=element_text(size=18))

    #Theme settings part 4 (center main title, set to 18 font size)
    pvo <- pvo + theme(plot.title = element_text(hjust = 0.5, size = 18))

    #Guide settings
    pvo <- pvo + guides(colour = guide_legend(title.hjust = 0.5, override.aes = list(size = 2)))

    #Run volcano plot object to be saved by ggsave
    pvo

    #Save the volcano plot
    ggsave(paste(dir.out, "/", file.id, "_vs_E_wt_", tissue.id, "_", age.id, "_", data.id,".pdf", sep=""), width=7,
height=7)
  }
}
```

Supplementary Note 6 | Custom python script used for transcriptome off-target analysis, related to Extended Data Fig. 9a.

```
#!/usr/bin/env python
from pathlib import Path
import re

import numpy as np
import pandas as pd
from tqdm.notebook import tqdm
import matplotlib.pyplot as plt
from matplotlib import colormaps as cm
from matplotlib.colors import rgb_to_hsv, hsv_to_rgb
from matplotlib.backends.backend_pdf import PdfPages


CHR_ORDER = ['chr' + str(x) for x in range(1, 20)] + ['chrX'] + ['chrY']
CHR_SORTKEY = {x: i for i, x in enumerate(CHR_ORDER)}

REDI_OUT = Path('./')

for p in REDI_OUT.glob('*-temp'):
    print(p)
    df = pd.read_csv(p / 'intervals.txt', sep='\t', skiprows=1, names=['chr', 'start', 'end', '?', 'width', 'reason'])
    df['chr_key'] = df['chr'].apply(lambda x: CHR_SORTKEY.get(x, 1000))
    df = (df.sort_values(['chr_key', 'start'])
          .drop(columns='chr_key')
          .reset_index(drop=True))
    tot_cov_ct = 0
    a_to_g_ct = 0
    all_ots = []
    for _, row in tqdm(df.iterrows(), total=len(df)):
        chrom, start, end = row['chr'], row['start'], row['end']
        sub_p = p / f'{chrom}#{start}#{end}.gz'
        if not sub_p.exists():
            continue
        sub_df = pd.read_csv(sub_p,
                             sep='\t',
                             names=['chr', 'pos', 'ref', 'strand', 'cov-q30', 'meanQ', 'mapped'],
                             usecols=list(range(7)))
        sub_df['g_ct'] = sub_df['mapped'].apply(lambda x: eval(x)[2])
        filter_df = sub_df[(sub_df['ref'] == 'A') &
                           (sub_df['cov-q30'] >= 10)]
        tot_cov_ct += filter_df['cov-q30'].sum()
        a_to_g_ct += filter_df['g_ct'].sum()
        if filter_df['g_ct'].sum() > 0:
            all_ots.append(filter_df[filter_df['g_ct'] > 0])
    all_ots = pd.concat(all_ots, ignore_index=True)
    all_ots.to_csv(p / 'A-to-G_concat.txt.gz', sep='\t', compression='gzip', index=False)
    with (p / 'result.txt').open('w+') as f:
        f.write(f'Total coverage: {tot_cov_ct}\n')
        f.write(f'Converted to G: {a_to_g_ct}\n')
        f.write(f'Average A-to-I editing: {a_to_g_ct / tot_cov_ct}')
```

```
meta_df = pd.read_csv('../metadata.csv', index_col=0)

CHR_ORDER = ['chr' + str(x) for x in range(1, 20)] + ['chrX'] + ['chrY']
CHR_SORTKEY = {x: i for i, x in enumerate(CHR_ORDER)}

NAME_RE = re.compile(r'(\d+)_.+_(\d+)_(\d+)-temp')

REDI_OUT = Path('./')
meta_paths = {}
for p in REDI_OUT.glob('*-temp'):
    idx, id1, id2 = NAME_RE.match(p.name).groups()
    name = f'{idx}_{id1}_{id2}'
    meta_paths[name] = p.name
meta_df['path'] = meta_paths

chrom_df =
pd.read_csv('/broad/liulabdata/Alvin_Hsu/0_Tools/genomes/GRCm39.primary_assembly.genome.fa.fai',
                    skiprows=0, names=['chr', 'len', 'offset'], usecols=[0, 1, 2], sep='\t', index_col=0)
chrom_df['mod2'] = [x % 2 for x in range(len(chrom_df))]

M_6WK_DOSEB = ['1_6wks_doseB_329_195-temp', '2_6wks_doseB_329_196-temp']
F_6WK_DOSEB = ['6_6wks_doseB_329_285-temp', '7_6wks_doseB_329_286-temp']
NEGS = meta_df[meta_df['dose'] == 'doseE']['path'].tolist()

def proc_dir(folder):
    df = pd.read_csv(f'{folder}/A-to-G_concat.txt.gz', sep='\t', compression='gzip')
    df = df.join(chrom_df, on='chr')
    df['x'] = df['offset'] + df['pos']
    df['y'] = df['g_ct'] / df['cov-q30']
    df = df[df['x'] < 2768805296]
    df = df.set_index('x')
    return df

m1 = set(proc_dir(M_6WK_DOSEB[0]).index)
m2 = set(proc_dir(M_6WK_DOSEB[1]).index)
f1 = set(proc_dir(F_6WK_DOSEB[0]).index)
f2 = set(proc_dir(F_6WK_DOSEB[1]).index)
negs = [(x, set(proc_dir(x).index)) for x in NEGS]
pair_negs = [s1 & s2 for i, (x1, s1) in enumerate(negs) for x2, s2 in negs[i + 1:]]
NEG_INDEX = set()
for s in pair_negs:
    NEG_INDEX |= s

POS_INDEX = (m1 & m2) | (f1 & f2)

OT_IDXS = POS_INDEX - NEG_INDEX

def darken(rgb, frac):
    h, s, v = rgb_to_hsv(rgb[0:3])
    return tuple(hsv_to_rgb((h, s, frac*v))) + (1.0,)

cmap = cm.get_cmap('viridis')
color = {'doseB': cmap(0.8), 'doseC': cmap(0.5), 'doseD': cmap(0.3), 'doseE': cmap(0.1)}
darks = {'doseB': darken(color['doseB'], 0.75),
         'doseC': darken(color['doseC'], 0.65),
```

```
        'doseD': darken(color['doseD'], 0.6),
        'doseE': darken(color['doseE'], 0.5)}

def jitter(x, y, c='b', ax=None):
    if ax is None:
        ax = plt
    x = 0.8 * np.random.random(size=y.size) + x - 0.9
    plt.scatter(x, y, color=c, s=0.1, alpha=0.75)


def proc_dir(folder):
    df = pd.read_csv(f'{folder}/A-to-G_concat.txt.gz', sep='\t', compression='gzip')
    df = df.join(chrom_df, on='chr')
    df['x'] = df['offset'] + df['pos']
    df['y'] = df['g_ct'] / df['cov-q30']
    df = df[df['x'].isin(OT_IDXS)]
    return df

# 6 weeks
f = plt.figure(figsize=(9, 6.5), dpi=300)
ax = plt.subplot(111)
for i, (_, row) in enumerate(meta_df[(meta_df['group'] == 'mut') &
                                   (meta_df['time'] == '6wks')].sort_values(['time', 'dose', 'sex']).iterrows(), 1):
    df = proc_dir(row['path'])
    jitter(i, 100*df['y'], color[row['dose']] if row['sex'] == 'Female' else darks[row['dose']], ax=ax)
plt.xlim(0, i)
plt.ylim(-1, 101)
for dose in ['doseB', 'doseC', 'doseD', 'doseE']:
    plt.scatter([-1], [-1], color=color[dose], s=1, alpha=0.75, label=f'{dose} Female')
    plt.scatter([-1], [-1], color=darks[dose], s=1, alpha=0.75, label=f'{dose} Male')
for x in [4, 8, 15]:
    plt.axvline(x, c='k', alpha=0.5, linewidth=1)
plt.xticks([])
ax.legend(loc='center left', bbox_to_anchor=(1, 0.5))
plt.title('Transcriptome-wide A-to-I editing (6 weeks)')
plt.ylabel('% of transcriptome reads containing\nA-to-I editing at specified positions')
plt.tight_layout()
plt.savefig('../250203_Manhattan/Jitter_6wks.pdf')
plt.close()


# 16 weeks
f = plt.figure(figsize=(9, 6.5), dpi=300)
ax = plt.subplot(111)
for i, (_, row) in enumerate(meta_df[(meta_df['group'] == 'mut') &
                                   (meta_df['time'] == '16wks')].sort_values(['time', 'dose', 'sex']).iterrows(), 1):
    df = proc_dir(row['path'])
    jitter(i, 100*df['y'], color[row['dose']] if row['sex'] == 'Female' else darks[row['dose']], ax=ax)
plt.xlim(0, i)
plt.ylim(-1, 101)
for dose in ['doseB', 'doseC', 'doseD', 'doseE']:
    plt.scatter([-1], [-1], color=color[dose], s=1, label=f'{dose} Female')
    plt.scatter([-1], [-1], color=darks[dose], s=1, label=f'{dose} Male')
for x in [4, 8, 12]:
    plt.axvline(x, c='k', alpha=0.5, linewidth=1)
plt.xticks([])
```

```
ax.legend(loc='center left', bbox_to_anchor=(1, 0.5))
plt.title('Transcriptome-wide A-to-I editing (16 weeks)')
plt.ylabel('% of transcriptome reads containing\nA-to-I editing at specified positions')
plt.tight_layout()
plt.savefig('../250203_Manhattan/Jitter_16wks.pdf')
plt.close()


def manhattan(row):
    df = proc_dir(row['path'])
    df['c'] = df['mod2'].apply(lambda x: color[row['dose']] if x else darks[row['dose']])
    plt.scatter(df['x'], 100*df['y'], s=1, c=df['c'])
    plt.xlim(0, 2768805296)
    plt.ylim(-1, 101)
    plt.title(f'Transcriptome-wide A-to-I editing\n{row["dose"]} {row["sex"]}, {row["time"].replace("wks", "
weeks")} [{row.name}]')
    plt.xticks([])
    xticks = []
    xlabels = []
    for i, (_, row) in enumerate(chrom_df.iterrows(), 1):
        if row['offset'] < 2768805296:
            plt.axvline(row['offset'], c='k', alpha=0.1, linewidth=1)
        if i in [1, 5, 10, 15, 20, 21]:
            middle = row['offset'] + 0.5*row['len']
            xticks.append(middle)
            xlabels.append(row.name[3:])
    plt.xticks(xticks, xlabels)
    plt.ylabel('% of transcriptome reads mapped to\nposition containing A-to-I editing')
    plt.tight_layout()

with PdfPages('../250203_Manhattan/Combined.pdf') as pdf:
    for i, row in meta_df.iterrows():
        plt.figure(figsize=(9, 6.5))
        manhattan(row)
        plt.savefig(f'../250203_Manhattan/{row.name}.svg')
        pdf.savefig()
        plt.close()
```

Supplementary Methods

Liquid chromatography-mass spectrometry (LC-MS/MS)

C26:0-lysophosphatidylcholine (C26:0-LPC) and plasmalogen species were extracted and quantified by liquid chromatography tandem mass spectrometry (LC–MS/MS) as previously described[2,3].

Supplementary References

1. Wunderling, K., Zurkovic, J., Zink, F., Kuerschner, L. & Thiele, C. Triglyceride cycling enables modification of stored fatty acids. *Nat. Metab.* **5**, 699–709 (2023).

2. Yoshioka, N. & Dowdy, S. F. Enhanced generation of iPSCs from older adult human cells by a synthetic five-factor self-replicative RNA. *PLoS ONE* **12**, e0182018 (2017).

3. Moser, A. B. *et al.* Plasma very long chain fatty acids in 3,000 peroxisome disease patients and 29,000 controls. *Ann. Neurol.* **45**, 100–110 (1999).
