# Supplementary material for: In vivo base editing rescues liver pathophysiology and peroxisome dysfunction in a mouse model of Zellweger spectrum disorder
Source: Nat Biomed Eng. Author manuscript; Available in PMC 2026 Jun 12. (PMC13262281; doi:10.1038/s41551-026-01651-5)
Supplement: Reporting summary [file NIHMS2176319-supplement-Reporting_summary.pdf]

nature portfolio

Corresponding author(s): David Liu, Cathleen Lutz

Last updated by author(s): 2026/01/21

Reporting Summary

Nature Portfolio wishes to improve the reproducibility of the work that we publish. This form provides structure for consistency and transparency in reporting. For further information on Nature Portfolio policies, see our Editorial Policies and the Editorial Policy Checklist.

Statistics

For all statistical analyses, confirm that the following items are present in the figure legend, table legend, main text, or Methods section.

n/a	Confirmed	
☐	☒	The exact sample size (*n*) for each experimental group/condition, given as a discrete number and unit of measurement
☐	☒	A statement on whether measurements were taken from distinct samples or whether the same sample was measured repeatedly
☐	☒	The statistical test(s) used AND whether they are one- or two-sided<br>*Only common tests should be described solely by name; describe more complex techniques in the Methods section.*
☐	☒	A description of all covariates tested
☐	☒	A description of any assumptions or corrections, such as tests of normality and adjustment for multiple comparisons
☐	☒	A full description of the statistical parameters including central tendency (e.g. means) or other basic estimates (e.g. regression coefficient) AND variation (e.g. standard deviation) or associated estimates of uncertainty (e.g. confidence intervals)
☐	☒	For null hypothesis testing, the test statistic (e.g. *F*, *t*, *r*) with confidence intervals, effect sizes, degrees of freedom and *P* value noted<br>*Give P values as exact values whenever suitable.*
☒	☐	For Bayesian analysis, information on the choice of priors and Markov chain Monte Carlo settings
☒	☐	For hierarchical and complex designs, identification of the appropriate level for tests and full reporting of outcomes
☐	☒	Estimates of effect sizes (e.g. Cohen's *d*, Pearson's *r*), indicating how they were calculated

Our web collection on statistics for biologists contains articles on many of the points above.

Software and code

Policy information about availability of computer code

Data collection	Illumina Miseq Control software was used on the Illumina Miseq sequencers to collect the high-throughput sequencing data. Droplet Digital PCR data was collected using the Bio-Rad QX ONE platform. Protein expression of ABCD3 was analyzed using the Compass for simple wes.
Data analysis	CRISPResso2 was used to analyze Miseq data for quantifying %edit and %indels at genomic loci. Droplet digital PCR data was analyzed using QX ONE software 1.3 Standard Edition. AmpUMI was used to deduplicate samples when noted. Code used for processing and analyzing high-throughput sequencing data are available at https://github.com/pinellolab/CRISPResso2. Codes for analyzing RNA-seq and r-script (RStudio:version 2024.12.1_563) for analyzing and plotting lipids volcano plots are available in the supplementary notes. Histological analysis for the expression of periodic acid-Schiff and Oil-Red-O was performed using Cellprofiler software (version 4.2.4), pipeline are available in the supplementary information. Compass for Simple Western (version 6.2.0) was used to analyze the ABCD3 expression.

For manuscripts utilizing custom algorithms or software that are central to the research but not yet described in published literature, software must be made available to editors and reviewers. We strongly encourage code deposition in a community repository (e.g. GitHub). See the Nature Portfolio guidelines for submitting code & software for further information.

Data

Policy information about availability of data

All manuscripts must include a data availability statement. This statement should provide the following information, where applicable:

- Accession codes, unique identifiers, or web links for publicly available datasets
- A description of any restrictions on data availability
- For clinical datasets or third party data, please ensure that the statement adheres to our policy

The main data supporting the results in this study are available within the paper and its Supplementary Information. High-throughput DNA sequencing FASTQ files are available from the National Center of Biotechnology's Information Sequence Read Archive under BioProject (PRJNA1162752). AAV9 and lentiviral plasmids encoding editors will be available through Addgene. Other materials and data are available from the corresponding authors upon reasonable request.

Research involving human participants, their data, or biological material

Policy information about studies with human participants or human data. See also policy information about sex, gender (identity/presentation), and sexual orientation and race, ethnicity and racism.

Reporting on sex and gender	The sex of the human patient data from the registry was reported in the supplementary tables.
Reporting on race, ethnicity, or other socially relevant groupings	Race, ethnicity and other socially relevant grouping were not reported in this study.
Population characteristics	Patients were recruited internationally: 87% of reported patients were from North America, 2.7% from Australia, 1.3% from Europe and 0.7% from South America.
Recruitment	The recruitment criteria were described in ClinicalTrials.gov (NCT01668186). Participants were enrolled with consent, and did not received compensation.
Ethics oversight	This study (ClinicalTrial.goc id: NCT01668186) was approved by The Health Canada and Public Health Agency of Canada (PHAC) Research Ethics Board (research ethics approval #11-090-PED).

Note that full information on the approval of the study protocol must also be provided in the manuscript.

Field-specific reporting

Please select the one below that is the best fit for your research. If you are not sure, read the appropriate sections before making your selection.

☒ Life sciences ☐ Behavioural & social sciences ☐ Ecological, evolutionary & environmental sciences

For a reference copy of the document with all sections, see nature.com/documents/nr-reporting-summary-flat.pdf

Life sciences study design

All studies must disclose on these points even when the disclosure is negative.

Sample size	Sample sizes were determined based on literature precedence for genome editing experiments (e.g. Anzalone et al., Nature 2019). For all molecular, biochemistry and histology data a "n" of at least 3-6 animals was used. Sample size is indicated in the figure and/or figure legend.
Data exclusions	ABE-AAV-treated mice were excluded from the study when next generation DNA sequencing confirmed 0% DNA modification in Pex1 locus, and the viral copy assessment assay indicated no viral genomes in the liver tissues, indicating it is injection-related issues.
Replication	All experiments in main text and extended data figures were performed with three replicates (expect two replicates performed in Figure 5D and E) and all attempts at replication were successful. The number of mice for each experiment was specified in the legends with N≥3.
Randomization	Mammalian cells used in this study were grown under identical conditions; no randomization was used. For mouse studies, animals from multiple litters were randomly assigned to each experimental arm, including at least two independent litters by arm.
Blinding	Mammalian cells used in this study were grown under identical conditions; blinding was used during microscope analysis of immunofluorescence experiment samples. The dosing, in vivo data collection as well as the biochemical and image analysis and quantification was performed in a blind fashion to the conditions of the experiment and genotype of the animals.

Reporting for specific materials, systems and methods

We require information from authors about some types of materials, experimental systems and methods used in many studies. Here, indicate whether each material, system or method listed is relevant to your study. If you are not sure if a list item applies to your research, read the appropriate section before selecting a response.

Materials & experimental systems

n/a	Involved in the study
☐	☒ Antibodies
☐	☒ Eukaryotic cell lines
☒	☐ Palaeontology and archaeology
☐	☒ Animals and other organisms
☐	☒ Clinical data
☒	☐ Dual use research of concern
☒	☐ Plants

Methods

n/a	Involved in the study
☒	☐ ChIP-seq
☒	☐ Flow cytometry
☒	☐ MRI-based neuroimaging

Antibodies

Antibodies used	Rabbit Polyclonal ABCD3 antibody (for Western Blot) from Abcam (Cat#ab85550), Catalase Recombinant Rabbit Monoclonal Antibody from Thermo Fisher Scientific (Cat#702955), Mouse Monoclonal ABCD3 antibody from Abcam (Cat#ab211533), Goat anti-Rabbit IgG (H+L) Cross-Adsorbed Secondary Antibody, Alexa Fluor™ 594 from Thermo Fisher Scientific (Cat#A-11012), Goat anti-Mouse IgG (H +L) Cross-Adsorbed Secondary Antibody, Alexa Fluor™ 488 FROM Thermo Fisher Scientific (Cat#A-11001).
Validation	The antibodies were validated by imaging or WB shown on vendor's websites.

Eukaryotic cell lines

Policy information about cell lines and Sex and Gender in Research

Cell line source(s)	HEK293T (ATCC Cat#CRL-3216), N2A (ATCC Cat#CCL-131), HEK293T clone 17 (ATCC Cat#CRL-11268), Patient-derived PEX1 G843D/I700fs fibroblasts (Coriell Institute Cat#GM16510), Healthy donor derived fibroblasts (Coriell Institute #GM03348). ZSD patient-derived homozygous PEX1 G843D/G843D fibroblasts were obtained from Peroxisomal Diseases Laboratory at Kennedy Krieger Institute as a gift.
Authentication	All cells were authenticated by the supplier using STR analysis.
Mycoplasma contamination	All cell lines tested negative for mycoplasma.
Commonly misidentified lines (See ICLAC register)	None used.

Animals and other research organisms

Policy information about studies involving animals; ARRIVE guidelines recommended for reporting animal research, and Sex and Gender in Research

Laboratory animals	All the animals used corresponds to the species Mus musculus, and the strains listed below:<br>B6.Cg-Pex1tm1.1Sjms/Mmjax (Jax stock # 25065)<br>129S6.Cg-Pex1tm1.1Sjms/Mmjax (Jax stock # 30931)
Wild animals	This study does not involve the use of wild animals
Reporting on sex	Sex was considered in this study. All animal cohorts were enrolled in a sex-balanced fashion. Data were pooled when sex was not relevant in the outcomes. Animal cohorts were separated by sex when results were sex dependent.
Field-collected samples	This study do not involve field-collected samples
Ethics oversight	All animal experiments follow OLAW guidelines, and were performed in accordance with the NIH Guidelines and approved by the Institutional Animal Care and Use Committee at The Jackson Laboratory, protocol number 20029-1.

Note that full information on the approval of the study protocol must also be provided in the manuscript.

Clinical data

Policy information about clinical studies

All manuscripts should comply with the ICMJE guidelines for publication of clinical research and a completed CONSORT checklist must be included with all submissions.

Clinical trial registration	NCT01668186

Study protocol	Patients with Peroxisome Biogenesis Disorders (PBD) diagnosis were enrolled in our longitudinal, retrospective natural history study of PBD at the Research Institute of the McGill University Health Center with consent. We requested medical records from birth to study entry, and then at yearly intervals from each participant's health care institutions after authorization from the patient or parent/legal representative. Participants also had the option to be seen in consultation at the McGill University Health Centre on a yearly basis. All medical records and images from participants are entered anonymously in a database. Bio-specimen are collected.
Data collection	Relevant chart notes, medical records, images, laboratory and pathology reports from participants with at least one PEX1 c.2528G>A (p.Gly843Asp) allele were extracted from January 2012 to January 2022.
Outcomes	Patients without any liver related abnormalities were classified as no liver disease (normal); patients reported with any of hepatomegaly, jaundice, elevation of liver enzymes in blood or coagulopathy were considered to have some hepatic dysfunction; patients diagnosed cirrhosis, portal hypertension, esophageal varices, gastrointestinal bleeding, ascites or hepatic cancer were termed as severe liver disease.

Plants

Seed stocks	N/A
Novel plant genotypes	N/A
Authentication	N/A
