# Supplementary material for: In vivo base editing rescues liver pathophysiology and peroxisome dysfunction in a mouse model of Zellweger spectrum disorder
Source: Nat Biomed Eng. Author manuscript; Available in PMC 2026 Jun 12. (PMC13262281; doi:10.1038/s41551-026-01651-5)

Extended Data Figure 7d: Pex1$^{G844D/G844D}$ mice treated with ABE LNP at P28. Automated western blot at 10 weeks post-treatment.

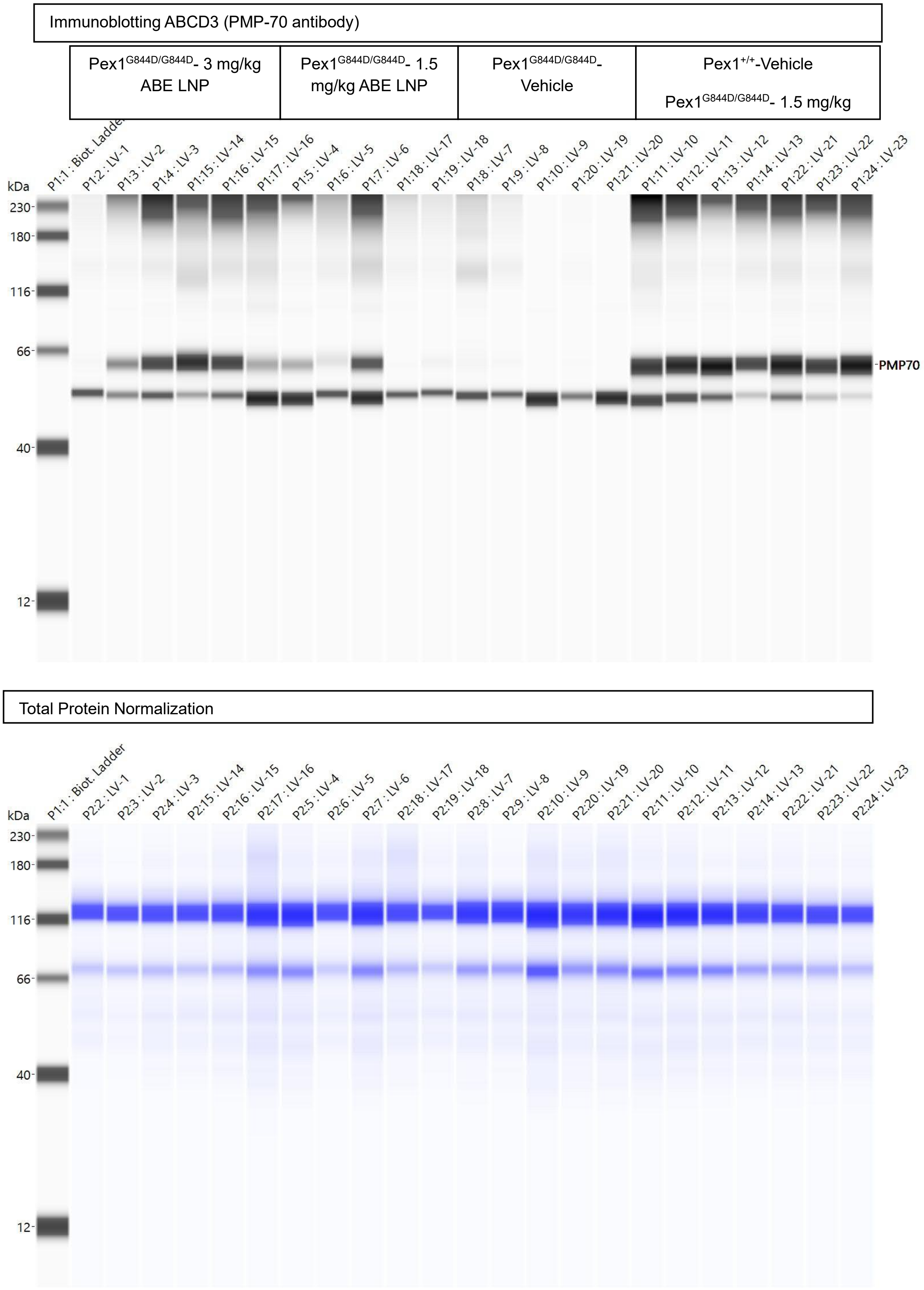

Supplement: Source data extended data [file NIHMS2176319-supplement-Source_data_extended_data.pdf]
