# Supplementary material for: In vivo base editing rescues liver pathophysiology and peroxisome dysfunction in a mouse model of Zellweger spectrum disorder
Source: Nat Biomed Eng. Author manuscript; Available in PMC 2026 Jun 12. (PMC13262281; doi:10.1038/s41551-026-01651-5)
Supplement: Source data fig 2d [file NIHMS2176319-supplement-Source_data_fig_2d.pdf]

Figure 2d: Pex1 $^{G844D/G844D}$ mice treated with ABE AAV. Automated western blot analysis at 6 weeks post-treatment.

Immunoblotting ABCD3 (PMP70 antibody)

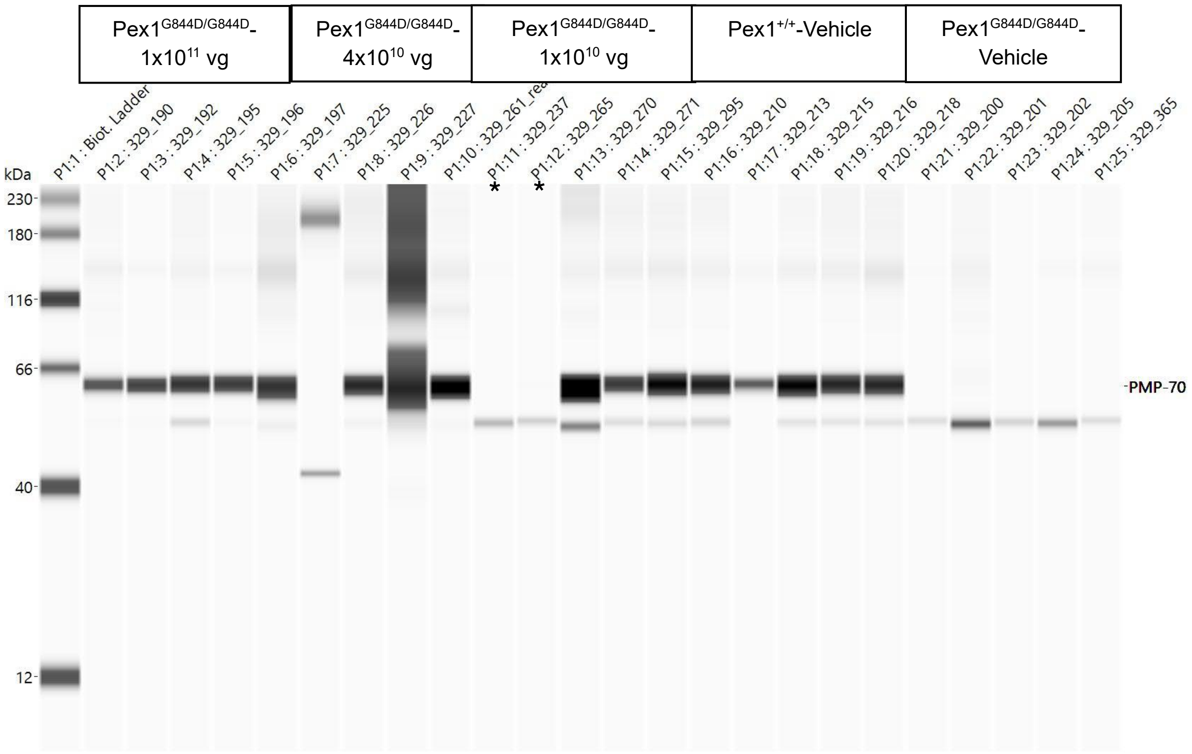

Total Protein Normalization

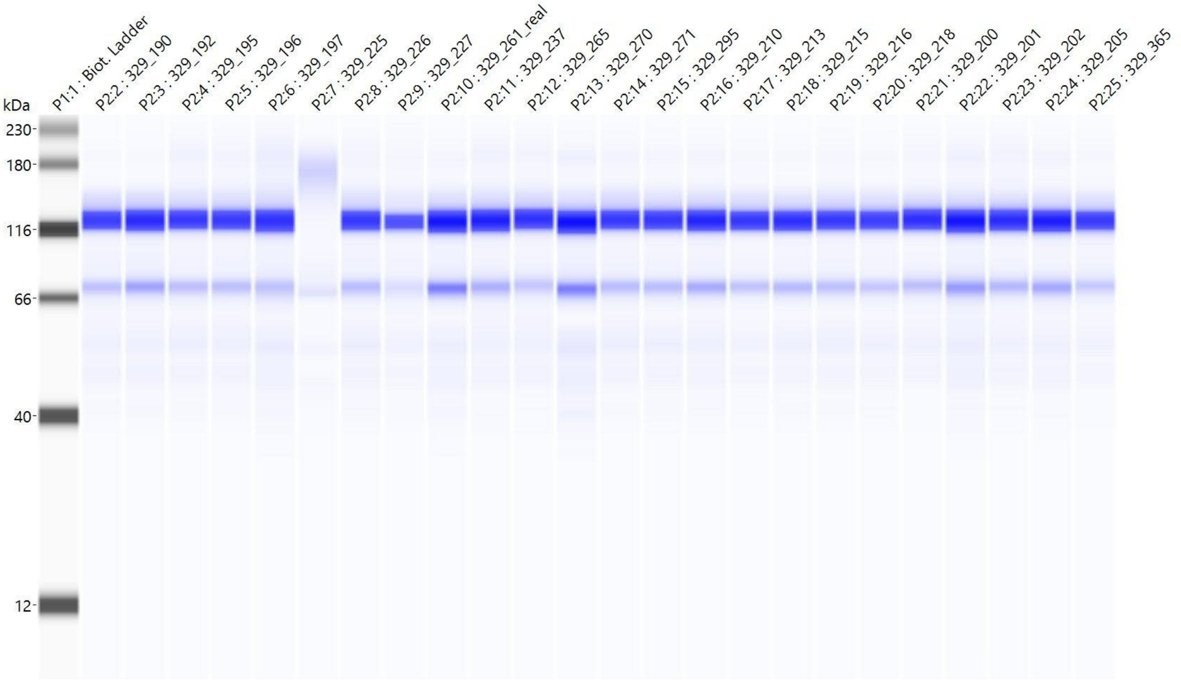

*Samples 329_237 and 329_265 were excluded.

Figure 2d: Pex1$^{G844D/G844D}$ mice treated with ABE AAV. Automated western blot analysis at 16 weeks post-treatment.

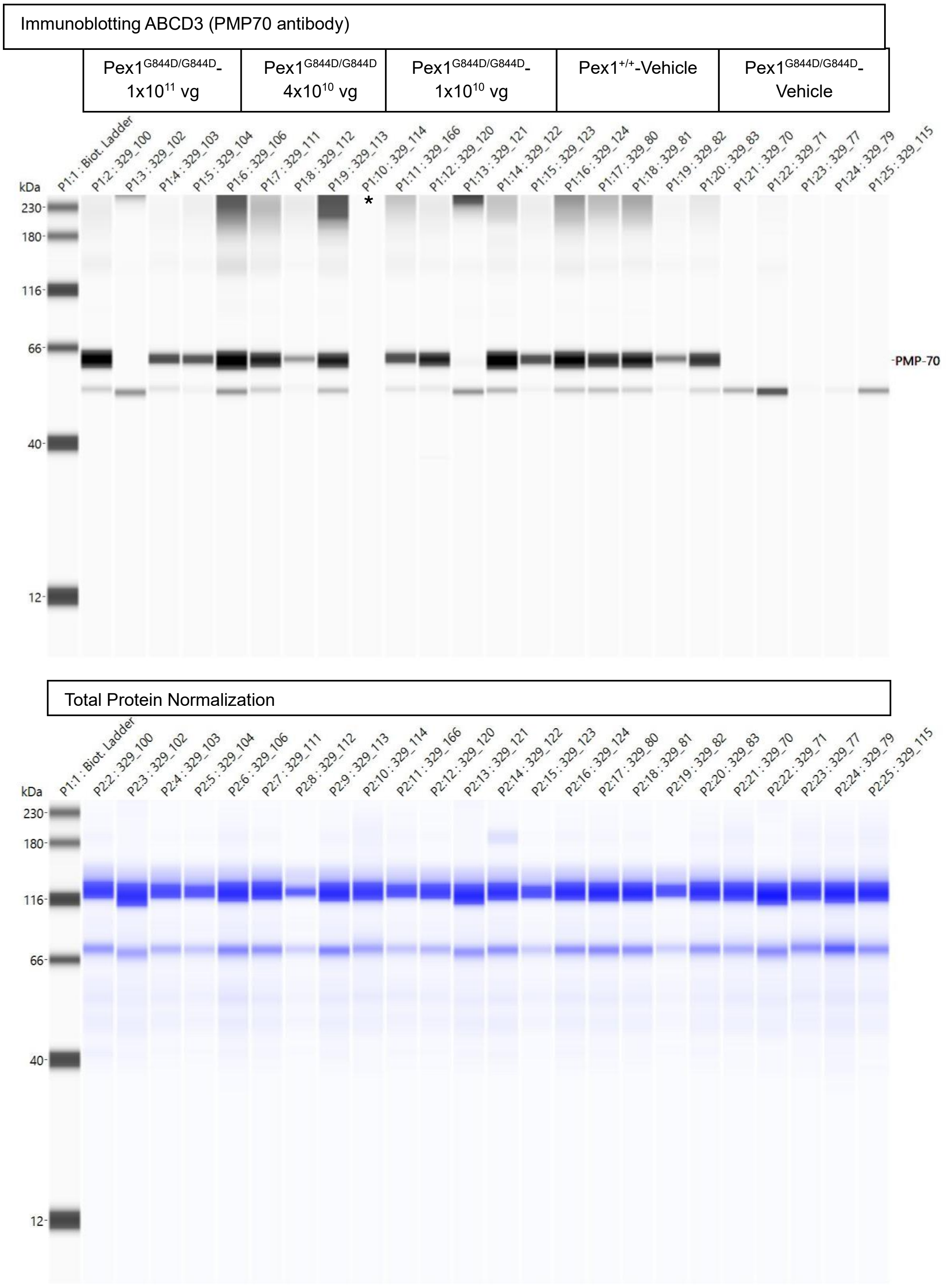

*329_114 was excluded.
