# Supplementary material for: In vivo base editing rescues liver pathophysiology and peroxisome dysfunction in a mouse model of Zellweger spectrum disorder
Source: Nat Biomed Eng. Author manuscript; Available in PMC 2026 Jun 12. (PMC13262281; doi:10.1038/s41551-026-01651-5)

Figure 4d: Pex1$^{G844D/G844D}$ mice treated with ABE AAV at P28, Automated western blot analysis at 16 weeks post-treatment.

Immunoblotting ABCD3 (PMP-70 antibody)

Pex1$^{G844D/G844D}$ 5x10^{11} vg	Pex1$^{G844D/G844D}$-Vehicle	Pex1$^{+/+}$-Vehicle

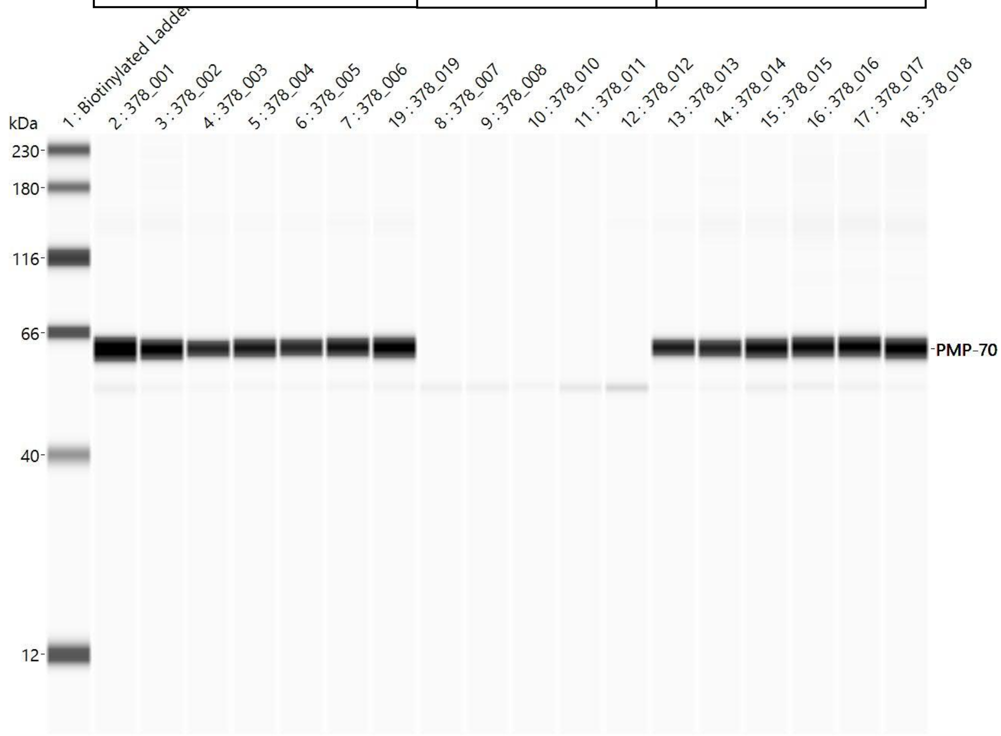

Total Protein Normalization

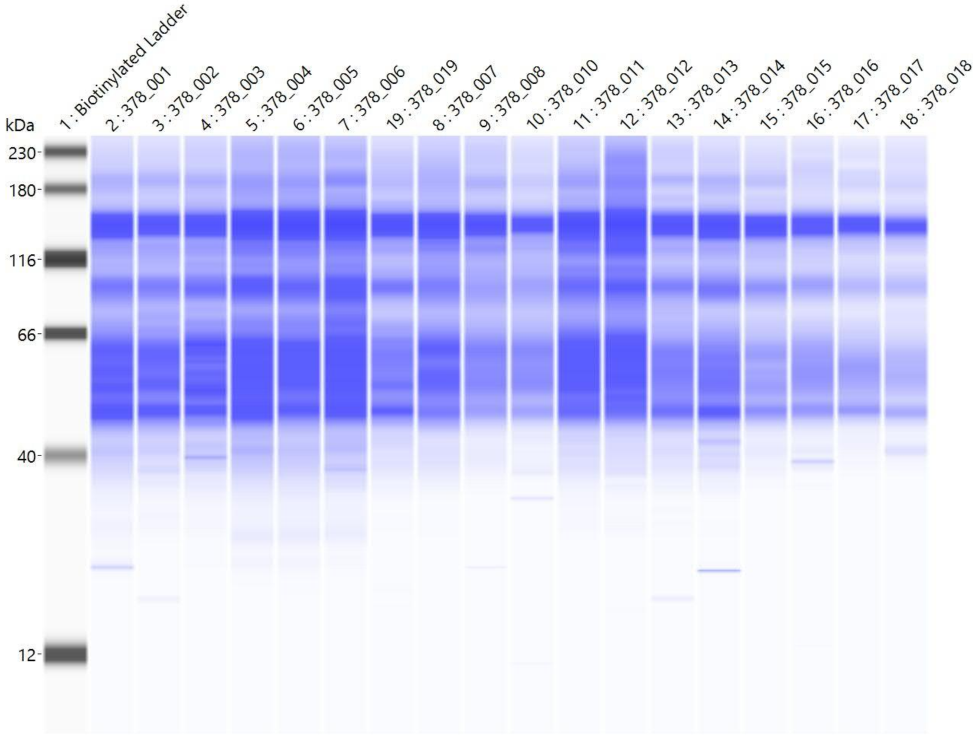

Supplement: Source data fig 4d [file NIHMS2176319-supplement-Source_data_fig_4d.pdf]
